# Borane-functionalized heteroscorpionate copper complexes as catalysts for azide–alkyne cycloaddition

**DOI:** 10.1039/d5dt01595b

**Published:** 2025-08-13

**Authors:** Tiago F. C. Cruz, Belén López-Sánchez, M. Amélia N. D. A. Lemos, Antonio Romerosa, Luísa M. D. R. S. Martins

**Affiliations:** a Centro de Química Estrutural, Departamento de Engenharia Química, Instituto Superior Técnico, Universidade de Lisboa Av. Rovisco Pais 1 1000-049 Lisboa Portugal carpinteirocruz@tecnico.ulisboa.pt luisammartins@tecnico.ulisboa.pt; b Institute of Applied Synthetic Chemistry, TU Wien Getreidemarkt 9/163-AC A-1060 Wien Austria; c Area de Química Inorgânica-CIESOL, Universidad de Almería Almería Spain; d CERENA, Departamento de Engenharia Química, Instituto Superior Técnico, Universidade de Lisboa Av. Rovisco Pais 1 1000-049 Lisboa Portugal

## Abstract

In this work, new copper(i) complexes of borane-functionalized bis(3,5-dimethylpyrazolyl)methane ligands were synthesized, characterized and used as catalysts for the cycloaddition of phenylacetylene and azides. Reaction of the allylated bis(3,5-dimethylpyrazolyl)methane proligand (La) with CuCl or [Cu(NCMe)_4_]BF_4_ gave rise to the neutral copper chloride complex [(La)CuCl] (1a) or the binuclear cationic copper complex [(La)Cu(NCMe)]_2_(BF_4_)_2_ (2a_2_), respectively. The same reactions using a borane-functionalized bis(3,5-dimethylpyrazolyl)methane proligand (Lb) led to the isolation of complexes [(Lb)CuCl] (1b) and [(Lb)Cu(NCMe)_2_]BF_4_ (2b). The new complexes were characterized by NMR and FTIR spectroscopies, elemental analysis, cyclic voltammetry and, in selected cases, single-crystal X-ray diffraction. Complexes 1a, 1b, 2a_2_ and 2b catalyzed the cycloaddition of phenylacetylene and *in situ* prepared benzyl azide to afford the respective 1,2,3-triazole products. When using complex 0.33 mol% of complex **2b** in the presence of 40 equivalents of diisopropylethylamine (DIPEA), a maximum yield of 97% with a maximum TOF value of 800 h^−1^ was obtained. Complex 2b also catalyzed the cycloaddition of phenylacetylene with 7 azides prepared *in situ* in 87%–99% yields. The borane-functionalized complexes 1b and 2b were, on average, 4-fold more active than their respective allylated analogues, and complex 2b readily led to the formation of the η^2^-phenylacetylene complex [(Lb)Cu(η^2^-PhC≡CH)]BF_4_ (4) and the presumed detection of borane-centered Lewis pairs with MesN_3_. These experimental observations, along with computational insights based on DFT calculations, indicated that the respective borane functionalities are beneficial to catalytic activity due to a substrate-directing effect.

## Abstract 2

New copper complexes with borane-functionalized heteroscorpionate bis(3,5-dimethylpyrazolyl)methane ligands were synthesized, characterized and used as precatalysts for the cycloaddition of phenylacetylene and azides.

## Graphical abstract

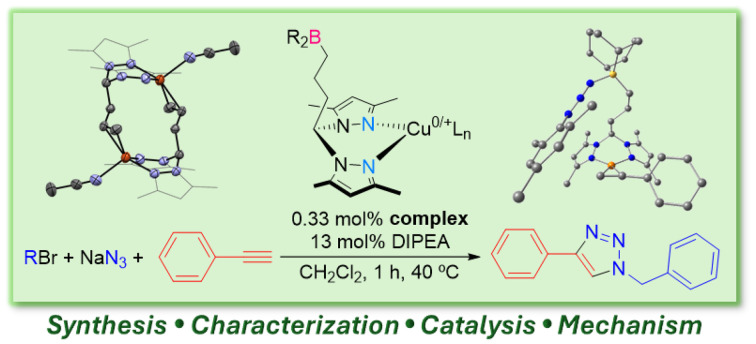

## Introduction

Copper-based systems are used as catalysts for a wide variety of organic reactions, such as C–H activation,^1^ living radical polymerization,^2,3^ oxidation^4^ and cross-coupling.^5^ Since Sharpless introduced the concept of click chemistry in 2001,^6^ copper(i) species have played an important role in catalyzing some of these reactions. In particular, regioselectivity displayed by copper(i) catalyst systems in the synthetically useful Huisgen 1,3-dipolar cycloaddition of azides and alkynes further drove their development.^7^ Therefore, this class of copper-catalyzed azide–alkyne cycloaddition reactions (CuAAC), independently and simultaneously discovered by Sharpless and Meldal,^8^ has become the subject of interest of many researchers.^9^ In general, click chemistry reactions allow for the synthesis of new materials with applications in drug development and biology.^10^ The versatile nature and applicability of CuAAC reactions were recognized by being the subject of the Nobel Prize in Chemistry in 2022.^11^

The first and simplest copper(i) sources employed in CuAAC reactions were based on CuSO_4_/sodium ascorbate^12^ or CuI/diisopropylethylamine (DIPEA),^13^ affording near quantitative yields even at catalyst loadings as low as 1 mol%. Although these catalyst systems, prepared *in situ*, proved effective, they exhibited a maximum turnover frequency (TOF) of only 27 h^−1^. By adding nitrogen-based ligands of varying denticity to the mentioned systems, a significant acceleration of the corresponding CuAAC reactions was observed.^14^ These results prompted further investigation into well-defined copper(i) molecular systems.^15^

**Chart 1:**
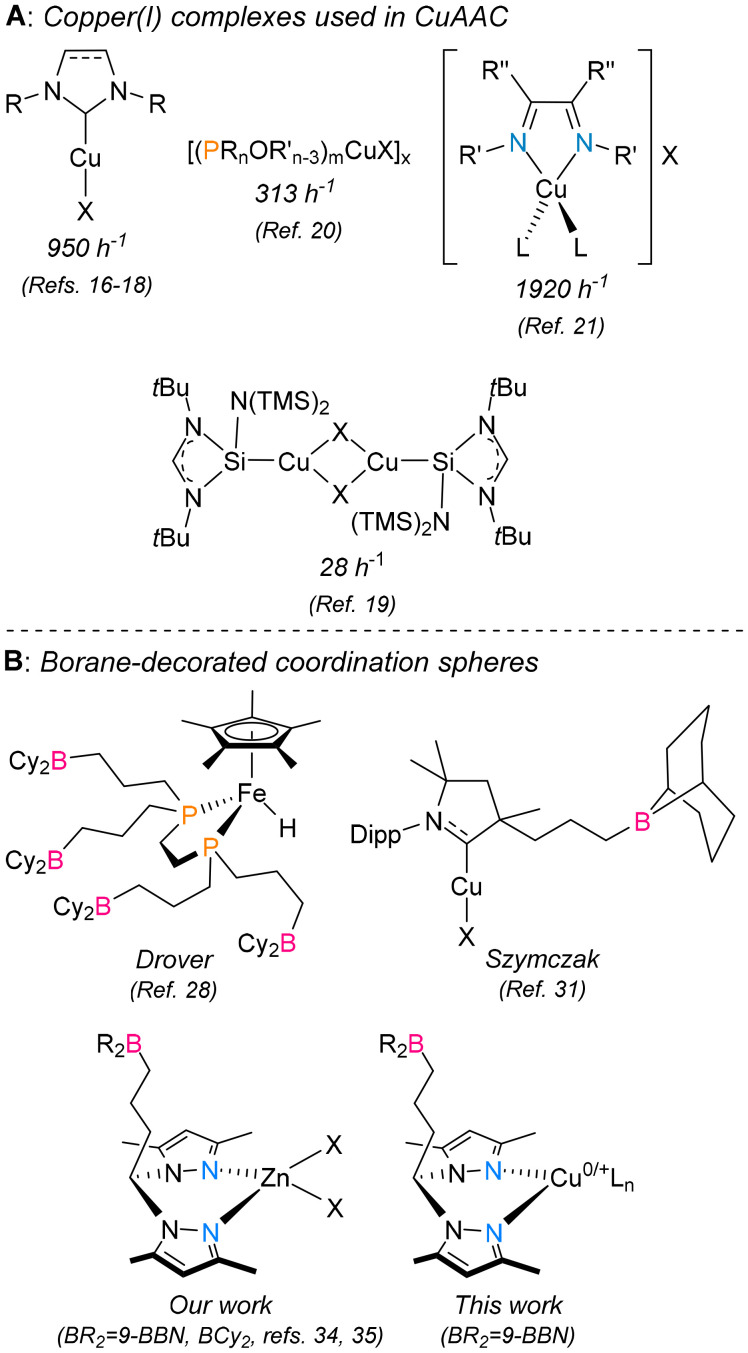
Copper(i) complexes used in CuAAC reactions (A) and examples of borane-decorated coordination spheres (B). The values denote the turnover frequencies of the catalysts.

Many well-defined copper(i) complexes have been used as catalysts in CuAAC reactions (Chart 1A). N-Heterocyclic carbenes,^16^ mesoionic carbenes,^17^ and isocyanides^18^ have been used as carbon-donor-based ligands for the preparation of copper(i) complexes, which have shown high catalytic activity, with TOF values of up to 950 h^−1^. Silylene copper(i) complexes have also been successfully used as CuAAC catalysts, achieving maximum TOF values of 28 h^−1^.^19^ Phosphines, phosphonites and phosphinites have also stabilized competent copper(i) catalysts for azide–alkyne cycloadditions (maximum TOF = 313 h^−1^).^20^ Nitrogen-based ligands have also been used to stabilize copper(i) catalysts for CuAAC reactions: for instance, diimine^21^ and polypyridine^22^ ligands were successfully employed, affording TOF values as high as 1920 h^−1^.

The manipulation of secondary coordination spheres in transition metal complexes has played important roles in the fields of metalloproteins^23^ and homogeneous catalysis.^24^ Combining Lewis acidic boranes with transition metal complexes is a way to achieve *push–pull* reactivity.^25^ This is characterized by the formation of Lewis pairs between borane and the ligands within the primary coordination sphere. The formation of such structures further activates the metal–ligand bonds or induces substrate-directing effects. The research groups of Bercaw,^26^ Klankermayer^27^ and Drover^28^ reported complexes containing mono- and bidentate borane-phosphine ligands and have detected borane-assisted small-molecule activation. Szymczak and coworkers reported stoichiometric and catalytic small-molecule activation in borane-functionalized pyridinepyrazolyl transition metal complexes,^29^ 1,4,7-triazacyclononane^30^ and cyclic (alkyl)(amino)carbene^31^ derivatives. Werlé and coworkers reported the hydrogenation of nitroarenes and the synthesis of silyl enol ethers catalyzed by borane-functionalized phosphinotriazine transition metal complexes.^32^ Nakao and coworkers also reported iridium-catalyzed *meta*-selective C–H borylation of benzamides and pyridines utilizing a borane-tethered phenanthroline ligand, in which the Lewis acid–base interactions promoted by the borane moiety induced a substrate-directing effect that amplified the selectivity of the reactions^33^ (Chart 1B).

In previous work, we reported the synthesis and characterization of zinc dichloride complexes of bis(3,5-dimethylpyrazolyl) methane ligands containing pendant borane functionalities.^34^ In that work, the new borane-functionalized complexes were active catalysts in the hydroboration of CO_2_ with pinacolborane to give the respective methyl boronic ester. In a follow-up report, bis(κ^2^-borohydride) zinc complexes utilizing the same ligands also catalyzed the hydroboration of CO_2_, isocyanates, esters and nitriles in good yields.^35^ In both studies, the zinc complexes bearing borane functionalities exhibited the best catalytic results. This effect was rationalized by the ability of the boranes to intra-/intermolecularly stabilize the hydride or oxygen/nitrogen-containing catalytic intermediates, through the reversible formation of Lewis pairs.

The synthetic simplicity and tunability of the bis(3,5-dimethylpyrazolyl) methane framework prompted us to pursue this system further. Considering the advantageous effect of using borane-functionalized bis(3,5-dimethylpyrazolyl) methane ligands to reversibly promote the formation of Lewis pairs with nitrogen-based substrates and catalytic intermediates, and given our previous experience in CuAAC reactions,^36^ we envisioned that it would be beneficial for the mediation of such reactions. To realize this goal, the present work features the synthesis and characterization of new copper(i) complexes bearing a borane-functionalized bis(3,5-dimethylpyrazolyl) methane ligand, their catalytic application to the cycloaddition of phenylacetylene and organic azides, and a rationalization of the respective roles of the appended boranes, using a combination of experimental and computational methods.

## Results and discussion

### Synthesis and characterization of the copper(i) complexes

**Scheme 1:**
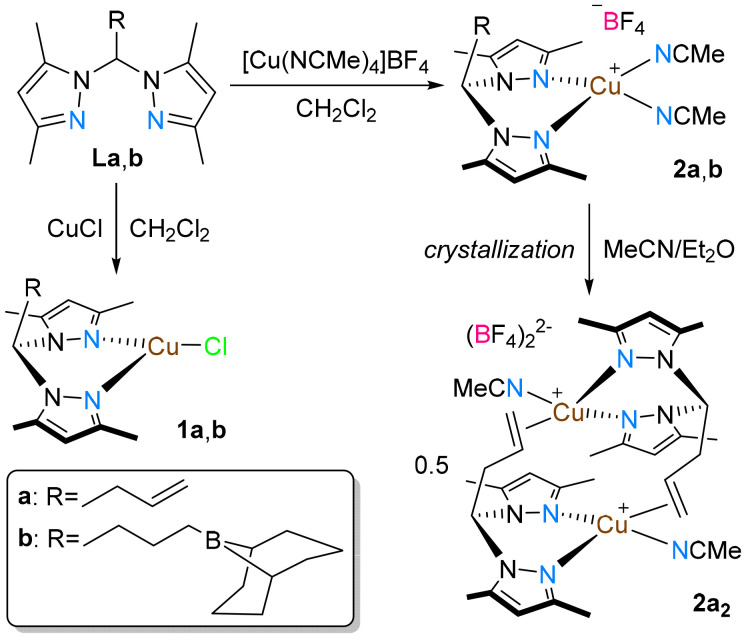
Synthesis of the copper complexes 1a, 1b, 2a/2a_2_ and 2b.

Reaction of the allylated (La, R=CH_2_CH=CH_2_) and borane-functionalized (Lb, R=CH_2_CH_2_CH_2_(9-borabicyclo[3.3.1]nonane) or CH_2_CH_2_CH_2_(9-BBN)) bis(3,5-dimethylpyrazolyl)methanes, described in a previous publication,^34^ with different copper(i) starting materials, led to the isolation of the neutral or cationic copper(i) complexes 1a, 1b, 2a_2_ and 2b (Scheme 1).

The neutral complexes [(La,b)CuCl] (1a and 1b) were prepared from the reaction of La or Lb and one equivalent of CuCl in dichloromethane. On the other hand, the reaction of La or Lb with one equivalent of [Cu(NCMe)_4_]BF_4_ in dichloromethane led to the isolation of the cationic complexes [(La)Cu(NCMe)_2_]BF_4_ (2a) and [(Lb)Cu(NCMe)_2_]BF_4_ (2b), respectively. However, after isolation of complex 2a from a dichloromethane/*n*-hexane mixture, NMR analysis of the crude product in CDCl_3_ revealed (1) the presence of a species with a stoichiometry of one La ligand to two coordinating acetonitrile ligands, and (2) that a white solid gradually precipitated from the freshly prepared solution. Analysis of this precipitate by NMR spectroscopy in CD_3_CN, in which it was fully soluble, showed the existence of a new species with a stoichiometry of one La ligand to one coordinating acetonitrile ligand, corresponding to the binuclear complex [(La)Cu(NCMe)]_2_(BF_4_)_2_ (2a_2_). The formation of the bis(acetonitrile) monomeric complex 2a was possible when an excess amount of acetonitrile was present in the reaction mixture. Upon evaporation of the volatile materials and redissolution in CDCl_3_, the excess of acetonitrile was eliminated, leading to the gradual precipitation of a solid (later identified as complex 2a_2_). Details of the experimental methodologies are provided in the SI. All new complexes were characterized by NMR and FTIR spectroscopic analysis (characterization data in Fig. S1–S21 in the SI) and elemental analysis. The propensity of complex 2a to dimerize to complex 2a_2_ under standard conditions limited its characterization to NMR spectroscopy.

The NMR spectra of the complexes present the expected resonances for the moieties in compounds La or Lb upon their coordination to copper. The 3,5-dimethylpyrazolyl CH resonances in the ligands are downfield-shifted upon coordination, going from 5.77 (La) or 5.76 (Lb) ppm to 5.87 (1a), 5.89 (1b), 5.98 (2a_2_) and 5.94 (2b) ppm in the different complexes. Likewise, the 3,5-dimethylpyrazolyl CH_3_ resonances change from singlets at 2.19 ppm in La,b to two distinct, upfield-shifted resonances at 2.25–2.46 ppm upon coordination. In the case of complexes 2a,b and 2a_2_, the NMR resonances for the coordinated acetonitrile ligands are also detected in the corresponding stoichiometries. The distinction between complexes containing ligands La or Lb is based on the detection or absence of the allylic olefin moieties in the respective NMR spectra. Complexes 1,2b present ^11^B NMR resonances at 87.5 (1b) and 86.2 (2b) ppm, characteristic of tricoordinate boranes simultaneously containing alkyl and 9-BBN/BCy_2_ fragments.^37^ The characterization of the complexes by FTIR spectroscopy evidenced the presence of 9-BBN and propylenic moieties by an increase in the intensity of the CH bond vibration modes in the range of 2750–2850 cm^−1^ in the Lb ligand in complexes 1,2b in comparison with those observed for the La ligand in complex 1a/2a_2_. The tetrafluoroborate B–F bond vibration modes were also detected in the cationic complexes 2a,b in the 1050–1100 cm^−1^ range.

**Fig. 1:**
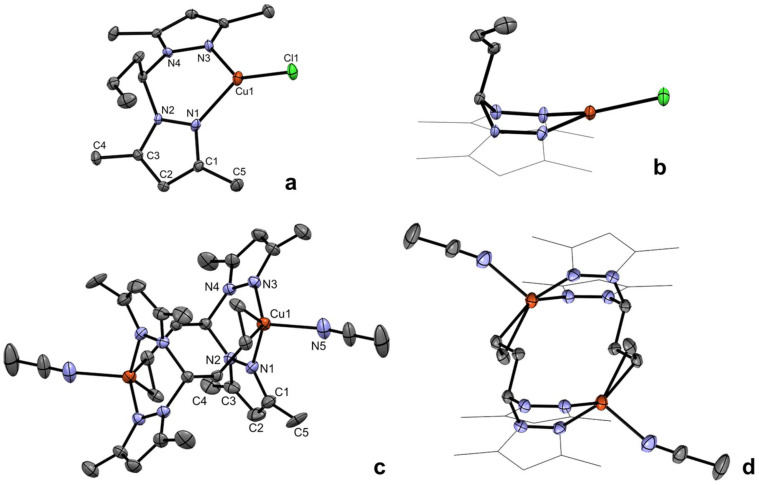
Single-crystal X-ray diffraction structures of complex 1a (a) and the cation of complex 2a_2_ (c) and their respective alternative perspective views showing the carbons of the pyrazolyl rings in a wireframe representation mode (b and d) with 30% probability ellipsoids. The hydrogen atoms and tetrafluoroborate anions are omitted for clarity.

Complexes 1a and 2a_2_ were characterized by single-crystal X-ray diffraction analysis. Complex 1a crystallized in the orthorhombic system, in the *Cc* space group, and complex 2a_2_ crystallized in the monoclinic system, in the *P*2_1_/*n* space group. A selection of bond lengths and angles, as well as the crystallographic data for the structures of complexes 1a and 2a_2_ are presented in Tables S1 and S2 in the SI. The molecular structures of complexes 1a and 2a_2_, along with the respective alternative perspective views, are presented in Fig. 1.

The structure of complex 1a displays a tricoordinate copper center containing a chloride ligand and the La ligand in a bidentate coordination mode. The N1–Cu1–Cl1, N3–Cu1–Cl1 and N1–Cu1–N3 bond angles are 129.91(12), 137.02(12) and 93.06(16)°, respectively, summing up to 359.99°. This indicates that the coordination geometry is virtually trigonal planar (sum of bond angles equal to 360°). The ring of chelation in complex 1a adopts a boat configuration, the α and β ring puckering parameters,^38^ respectively defined as the angles between the {N1, N2, N3, N4} and {C11, N2, N4} and {N1, N2, N3, N4} and {Cu1, N1, N3} planes, are 54.2(5) and 16.5(3)°, respectively. The structural features of complex 1a have previously been observed in other crystallographically characterized copper complexes bearing bis(pyrazolyl)methane ligands.^39^

The molecular structure of 2a_2_ is a binuclear dicopper complex in which each metal center has a coordinated acetonitrile ligand, a κ^2^-coordinated bis(3,5-dimethylpyrazolyl) fragment from one La ligand and an η^2^-olefnic moiety from the allyl fragment in the opposing La ligand. Each of the copper centers in complex 2a_2_ presents a distorted pseudo-tetrahedral coordination geometry, with a τ_4_ parameter^40^ equal to 0.79, when considering the centroid of the η^2^-olefnic moiety as one of the edges of the imaginary pseudo-tetrahedron. The Cu1–N bond lengths are in the range of 2.022(3)–2.117(4) Å, while the Cu1–C bond lengths are in the range of 2.118(4)–2.170(4) Å. The α and β ring puckering parameters in complex 2a_2_ are 52.9(3) and 22.1(2)°, respectively. Binding motifs such as that observed in the structure of 2a_2_ were reported by Drover and coworkers, where pending allyl moieties were present in the ligand frameworks.^28^

The copper(i) complexes 1a, 1b, 2a_2_ and 2b were also analyzed by cyclic voltammetry. They all undergo an oxidation process, with half-wave anodic potentials ranging from 0.30 V (2b) to 0.57 V (2a_2_), with the latter value reflecting the greater difficulty of oxidizing the binuclear dicationic complex, while complex 2b showed the lowest oxidation potential, undergoing a partial chemically irreversible oxidation process in the 20 mV s^−1^ to 2 V s^−1^ scan rate range. The electrochemical behavior of these complexes is described in more detail in the SI, Table S3 and Fig. S38.

### Catalytic cycloaddition of benzyl azide and phenyl acetylene mediated by the copper complexes

The catalytic activities of copper(i) complexes 1a, 1b, 2a_2_ and 2b were evaluated for the cycloaddition of benzyl azide and phenylacetylene in CH_2_Cl_2_ at room temperature and 40 °C under dinitrogen atmosphere, in the presence or absence of DIPEA as a base. DIPEA was selected due to its solubility in dichloromethane and substrates. In all tests, benzyl azide was formed *in situ* by the reaction of benzyl bromide and NaN_3_ in a 1 : 1 ratio. Experiments employing different catalyst loadings (1 or 0.33 mol%) and varying base concentrations relative to the catalysts (20 or 40 equivalents) were also conducted. Additional experiments were performed at different temperatures, specifically room temperature (RT) and 40 °C. The results for all the catalytic reactions performed in this work are summarized in Tables S4–S7 in the SI.

When 1 mol% of complex 2a_2_ was used as a catalyst, azide/alkyne ratios of 1 : 1 and 1 : 1.5 were tested for the cycloaddition of benzyl azide and phenylacetylene at 40 °C. The results showed that an azide/alkyne ratio of 1 : 1.5 provided a higher conversion rate of 55% (TON = 55, TOF_2.5 h_ = 22 h^−1^), compared to that obtained with a 1 : 1 ratio (conversion of 10%, TON = 10, TOF_2.5 h_ = 4 h^−1^). To enhance the catalytic efficiency of this complex, reactions were evaluated in the presence of an excess of base relative to complex 2a_2_. It was observed that an excess of DIPEA accelerates the reaction, with complete cycloaddition occurring when 20 equivalents of DIPEA were used at 40 °C (TON = 98, TOF_2.5 h_ = 39 h^−1^). Performing the reaction at room temperature resulted in only 9% conversion of phenylacetylene (TON = 9, TOF_2.5 h_ = 4 h^−1^).

When 1 mol% of the neutral complex 1a was used, the reaction was nearly complete in the presence of 20 equivalents of DIPEA both at room temperature and 40 °C. Subsequently, the catalyst loading was reduced to 0.33 mol%, achieving 54% conversion after 1 hour (TOF_1 h_ = 137 h^−1^). Lower conversions were obtained with 20 equivalents of DIPEA using complex 1a than with complex 2a_2_, both at 40 °C with only 3% conversion (TON = 13, TOF_2.5 h_ = 5 h^−1^), and at room temperature, with only 11% conversion even after of 2.5 h (TON = 13, TOF_2.5 h_ = 5 h^−1^). Similar to the observations with complex 2a_2_, the best results using complex 1a were obtained with 40 equivalents of DIPEA at 40 °C, with the highest conversion (87%) being reached after 2.5 h with TOF_2.5 h_ = 106 h^−1^.

The use of complex 1b led to a higher conversion at 40 °C with 40 equivalents of DIPEA, achieving 98% conversion in 2.5 h (TON = 297, TOF_2.5 h_ = 119 h^−1^). However, the catalytic activity of 1b (64% conversion, TON = 194, TOF_2.5 h_ = 78 h^−1^) at room temperature was higher compared to those with other complexes (1a: 87% conversion, TON = 264, TOF_2.5 h_ = 106 h^−1^; 2a_2_: 91% conversion, TON = 276; TOF_2.5 h_ = 110 h^−1^).

The monomeric cationic complex containing the 9-BBN borane functionalized 2b was tested, examining similar parameters such as the amount of catalyst and base, as well as the reaction temperature. As with the other complexes, a large excess of DIPEA and elevated temperature (40 °C) were necessary to activate the catalytic process. Notably, the highest TOF obtained for the cycloaddition of benzyl azide and phenylacetylene in the work (294 h^−1^) was achieved at 40 °C with complex 2b, after 1 h, using 0.33 mol% of complex, and 40 equivalents of DIPEA. Additionally, the catalytic reactions performed under aerobic conditions gave results comparable to those obtained under inert conditions (under N_2_: TOF_1 h_ = 294 h^−1^, under air: TOF_1 h_ = 276 h^−1^).

Table 1 compiles the most significant catalytic outcomes for all the complexes studied, including the best conversion, TON, and TOF values observed in the cycloaddition of benzyl azide and phenylacetylene under the optimized conditions, considering the influence of various experimental parameters.

**Table 1:** Summary of catalytic cycloaddition of benzyl azide and phenylacetylene catalyzed by the tested complexes^a^

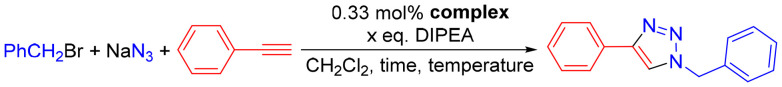							
Complex	[Cu] (mol%)	Temperature (°C)	Time (min)	Eq. DIPEA	TON	TOF (h^−1^)	Conversion (%)
1a	1	40	150	20	99	40	99
1a	1	RT	150	20	98	39	98
1a	0.33	40	60	40	164	137	54
1a	0.33	40	150	40	264	106	87
2a_2_^b^	1	40	40	20	98	39	98
2a_2_^b^	1	RT	RT	20	9	4	9
2a_2_^b^	0.33	40	60	20	46	46	15
2a_2_^b^	0.33	40	150	40	294	118	97
1b	0.33	40	60	40	270	270	89
1b	0.33	40	150	40	297	119	98
2b	0.33	40	60	40	294	294	97
2b	0.33	40	150	40	297	119	98
2b	0.33	40	0.25	40	200	800	66

a Conditions: azide/alkyne ratio of 1 : 1.5, solvent: dichloromethane.

b Value per copper center.

Table 2 shows a comparison of the catalytic activity at 0.33 mol% catalyst loading of the different complexes for the cycloaddition of benzyl azide and phenylacetylene under the optimized conditions, in the presence of DIPEA, at 40 °C and after 1 h of reaction. Table 2 shows that, under the same reaction conditions, the borane-functionalized complexes 1b and 2b outperformed their respective unfunctionalized analogues 1a and 2a_2_ by 4-fold, on average, indicating that the included borane functionality is beneficial for the present catalytic cycloaddition reactions.

**Table 2:** Comparison of the activities of the different copper(i) complexes for the catalytic cycloaddition of benzyl azide and phenylacetylene under the optimized conditions^a^

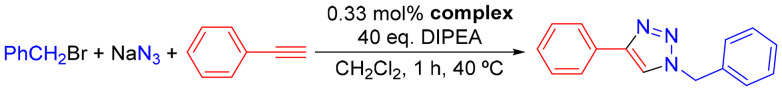		
Complex	Conversion (%)	TOF (h^−1^)
1a	54	137
1b	89	270
2a_2_^b^	15	46
2b	97	297

a Conditions: 0.33 mol% of complex, 40 eq. of DIPEA, azide/alkyne ratio of 1 : 1.5, time: 1 h, temperature: 40 °C, solvent: dichloromethane.

b Value per copper center.

Given that complex 2b showed the best catalysis results, the cycloaddition reaction between benzyl azide and phenylacetylene catalyzed by 0.33 mol% of complex 2b in the presence of 40 equivalents of DIPEA at 40 °C was monitored over time, as shown in Table S8 and Fig. S39 in the SI. This experiment achieved a maximum TOF value of 800 h^−1^ at a reaction time of 15 minutes, the time at which the reaction rate was also at its highest during monitoring. A short induction period was observed in the first 10 minutes of the reaction, likely due to the *in situ* formation of benzyl azide.

Complexes 1b and 2b, which exhibited the highest catalytic activity, showed the lowest oxidation potential. Interestingly, when the TOF is plotted against the half-wave potentials for the oxidation of the different complexes, a linear relationship is obtained, as depicted in Fig. S40 in the SI. The higher the oxidation potential, *i.e.* the harder it is to oxidize the complex, the lower the catalytic activity, as denoted by the lower TOF value.

**Scheme 2:**
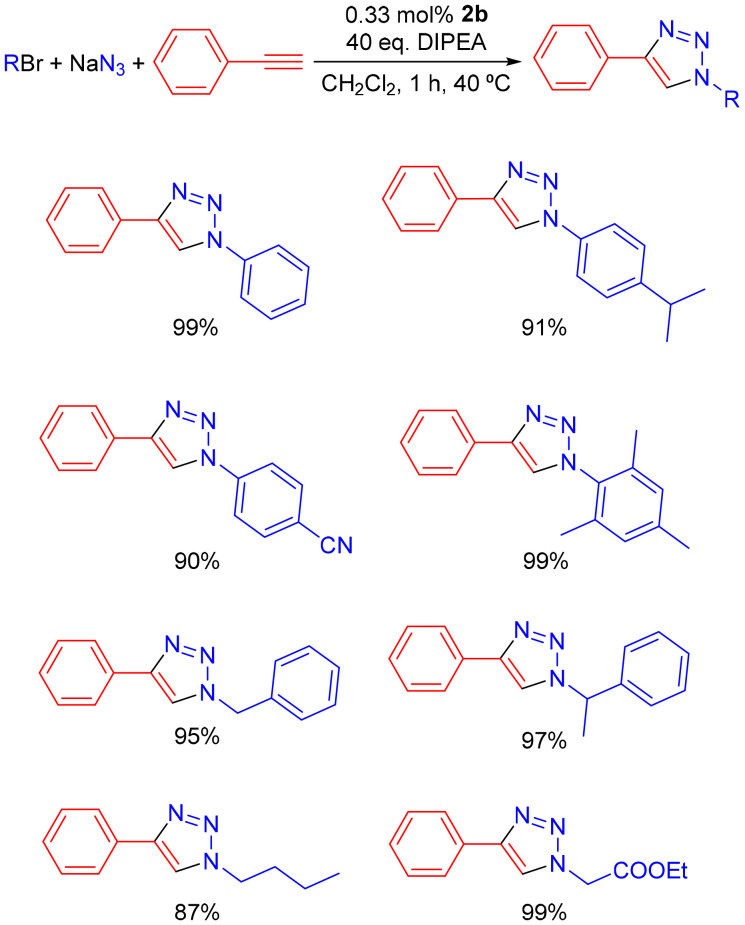
Substrate scope of the azide–alkyne cycloaddition reactions catalyzed by complex 2b in the presence of DIPEA. The percentages correspond to the isolated yields.

After the optimization of the reaction conditions, the substrate scope of the reaction was evaluated using complex 2b as the precatalyst, as shown in Scheme 2.

According to Scheme 2, complex 2b, in the presence of DIPEA, catalyzed the cycloaddition of several azides with phenylacetylene. The cycloaddition of phenylacetylene with stereochemically (mesityl) and electronically (4-isopropyl- and 4-fluorophenylazide) differentiated *in situ* prepared azides was successfully catalyzed in high yields (90–99%) to afford the respective 1,2,3-triazole products. Benzylic azides were also successfully employed, with the cycloaddition of phenylacetylene and benzyl- or methylbenzylazide achieved in yields of 95–97%. The aliphatic *n*-butylazide and a substrate containing an ester moiety (ethyl 2-azidoacetate) were also employed successfully, giving yields of 87 and 99%, respectively. Internal alkynes such as but-2-yne, prop-1-yn-1-ylbenzene and diphenylacetylene did not show any detectable activity. The ^1^H NMR spectra of the isolated triazole products are presented in Fig. S41–S48 in the SI and are consistent with data reported in the literature.^41^

The results obtained with the present system using complex 2b led to a maximum TOF value of 800 h^−1^, at concentrations of catalyst as low as 0.33 mol%. Considering this level of catalytic activity, the presented complexes are less efficient than the mononuclear copper complexes bearing N-heterocyclic carbenes (maximum TOF of 950 h^−1^)^16^ or diimine ligands (maximum TOF of 1920 h^−1^).^21^ However, complex 2b led to better catalytic activities than those achieved with copper(i) catalysts bearing phosphine/phosphonite/phosphinite ligands (maximum TOF of 313 h^−1^)^20^ and considerably better than *in situ* prepared systems (maximum TOF of 27 h^−1^).^12,13^ A selected comparison of CuAAC catalyst systems is presented in Table S9 in the SI. Although complexes 1b/2b constitute the third set of examples of copper complexes containing pendant borane functionalities,^30,31^ they are the first to have been used as catalysts in CuAAC reactions, and they all enabled an enhancement in catalytic activity.

### Considerations on the role of the appended borane functionalities

**Scheme 3:**
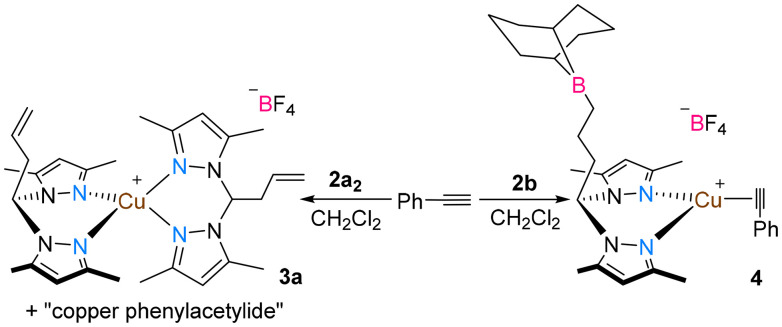
Reaction of complexes 2a_2_ and 2b with phenylacetylene: isolation of complexes 3a and 4.

To better understand the role of the borane functionalities included in the complexes, several control reactions and DFT calculations were performed. Among all complexes, the cationic complexes 2a_2_ and 2b were the ones that reacted with an excess of phenylacetylene (Scheme 3).

The reaction of complex 2a_2_ with phenylacetylene gave rise to the observation of the homoleptic complex [(La)_2_Cu]BF_4_ (3a) and a bright yellow solid that was insoluble in common organic solvents, likely corresponding to copper phenylacetylide,^42^ which is not active in CuAAC reactions.^43^ The reaction of complex 2b with phenylacetylene gave rise to the isolation of the cationic copper(i) containing a η^2^-phenylacetylene ligand [(Lb)Cu(η^2^-PhC≡CH)]BF_4_ (4). Complex 3a was rationally isolated by reaction of two equivalents of La with one equivalent of [Cu(NCMe)_4_]BF_4_ in dichloromethane. The homoleptic complex containing the borane functionalized ligand Lb[(Lb)_2_Cu]BF_4_ (3b) was also isolated by this synthetic route.

Complexes 3a, 3b and 4 were characterized by NMR and FTIR spectroscopies (Fig. S22–S36 in the SI) and elemental analysis. The 3,5-dimethylpyrazolyl methyl resonances in complex 3a are split into one sharp singlet and two broad singlets, indicating that the steric repulsion of the CH_3_ of the different ligands closer to the metal center induces fluxional behavior. This fluxional behavior, which is only observed in complex 3a, likely involves the slow inversion of the chelation rings in ligand La. The fact that the homoleptic complexes 3a and 3b present spectroscopic features that are distinct from those of complexes 2a, 2b and 2a_2_ further reinforces the heteroleptic characteristics of the latter. Although complex 3a was also analyzed by single-crystal X-ray diffraction, the poor quality of the crystal and the highly disordered nature of one of the 3,5-dimethylpyrazolyl moieties prompted us to only present its molecular structure in Fig. S37 in the SI as proof of its molecular connectivity. The ^1^H NMR spectrum of complex 4 displays resonances corresponding to one coordinated Lb ligand and phenylacetylene in a 1 : 1 stoichiometry. The ^1^H and ^13^C NMR signals corresponding to the coordinated Lb ligand in complex 4 are in line with the observations made for the heteroleptic complex 2b bearing the same ligand, while the acetylide ^1^H and ^13^C NMR signals appear at 5.29 and 81.1 ppm, respectively, significantly shifted from those of free phenylacetylene (3.06 and 77.3 ppm, respectively).^44^ The FTIR spectrum of complex 4 displays a band at 3450 cm^−1^, which is characteristic of acetylide C–H stretching.^45^ As determined by DFT calculations, the structure of the cation of complex 4 reveals a pseudo-trigonal planar coordination geometry, when considering the centroid of the η^2^-phenylacetylene ligand, which has Cu–C bond lengths in the range of 1.995–2.108 Å.

Analysis of complexes 3a and 4 by cyclic voltammetry showed for the former a reversible oxidation process at half-wave potential of 0.30 V, occurring for all scan rates from 50 mV s^−1^ to 2 V s^−1^, with a current intensity ratio between the anodic peak and the cathodic counterpart of 1, whilst the latter undergoes an irreversible oxidation at potential peak of 0.92 V, leading to the fast decomposition of the complex in solution. The electrochemical behavior of these complexes is described in more detail in the SI, Table S3 and Fig. S38.

The borane-functionalized complexes 1b and 2b reacted with mesitylazide (MesN_3_) as indicated by NMR spectroscopy (Fig. S49–S54 in the SI). The ^1^H NMR spectrum acquired from the reaction of complex 1b with MesN_3_ presented a set of new signals, while the ^11^B NMR spectrum was characterized by the disappearance of the resonance at 87.5 ppm, corresponding to the tricoordinated boron synthon in complex 1b, and the appearance of two new resonances at 0.3 and −0.3 ppm, indicative of the presence of tetracoordinate boron species (Fig. S50 in the SI). These observations point to the coordination of MesN_3_ to the 9-BBN moiety in two different modes *i.e.* terminal *vs.* internal azide nitrogen coordination,^46^ tentatively attributed to the Lewis-paired complexes [(MesN_3_)(Lb)CuCl] (1b·N_3_Mes). By its turn, the reaction of complex 2b with MesN_3_ was again accompanied by a new set of signals in the ^1^H NMR spectrum and the disappearance of the ^11^B NMR signal at 86.2 ppm, corresponding to the tricoordinated boron moiety in complex 2b, and the detection of two resonances at 0.6 and 0.0 ppm (Fig. S52 in the SI). These observations also point to the formation of borane-azide Lewis-paired complexes [(MesN_3_)(Lb)Cu(NCMe)_2_]BF_4_ (2b·N_3_Mes). When complex 2b was simultaneously reacted with one equivalent of phenylacetylene and MesN_3_, complex 4 was the only observed product. Reaction of complex 2b with phenylacetylene and MesN_3_ in the presence of excess DIPEA led to the formation of a new complex that presumably contained a boron-coordinated 1-mesityl-4-phenyl-1*H*-1,2,3-triazole, as indicated by the observation of the respective ^1^H and ^11^B NMR spectra. This points to the formation of the Lewis-paired complex [(MesN_3_)(Lb)Cu(NCMe)_2_]BF_4_ (4·Triazole). In addition, the reaction of complex 4 with DIPEA led to the formation of an unidentified new copper complex; however, there was no evidence for the formation of a Lewis pair between the appended borane and DIPEA. The latter result shows that DIPEA does not coordinate to boron at any point in the catalytic cycle. The high complexity of the product mixtures obtained in the stoichiometric reactions with MesN_3_ prevented their full characterization.

**Fig. 2:**
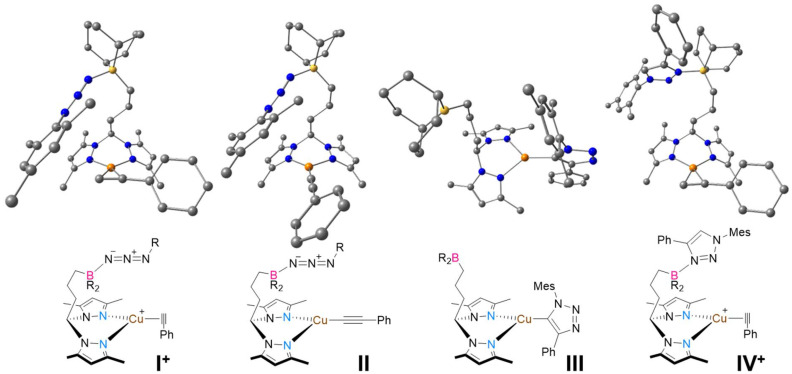
Molecular structures of complexes I^+^, II, III and IV^+^, as determined by DFT calculations. The hydrogen atoms are omitted for clarity and BR_2_ denotes 9-BBN.

DFT calculations^47^ were performed to further rationalize the role of the borane functionalities in the complexes. Details of the DFT calculations are described in the SI. The starting point for the geometry optimizations of the complexes using ligand La was the coordinates extracted from the single-crystal X-ray diffraction of complex 1a or 2a_2_. On the other hand, the starting point for the optimization of the complexes using ligand Lb was the single-crystal X-ray diffraction structure of the [(Lb)ZnCl_2_] obtained in a previous publication,^34^ due to its structural similarity to the present complexes. As shown in the control reactions, the boron centers in the complexes form Lewis pairs with MesN_3_ and with 1-mesityl-4-phenyl-1*H*-1,2,3-triazole, as indicated by the detection of complexes 1b·N_3_Mes, 2b·N_3_Mes and 4·Triazole. Geometry optimizations were therefore performed on selected structures containing the Lb ligand; namely, the Lewis pair of the cationic complex 4 with MesN_3_, [(MesN_3_)(Lb)Cu(η^2^-PhC≡CH)]^+^ (I^+^); the Lewis pair of the neutral copper phenylacetylide complex with MesN_3_, [(MesN_3_)(Lb)Cu(C≡CPh)] (II); the neutral copper 1-mesityl-4-phenyl-1*H*-1,2,3-triazolyl complex, [(Lb)Cu(Triazolyl)] (III); and the Lewis pair of the cationic complex 4 with 1-mesityl-4-phenyl-1*H*-1,2,3-triazole, [(Triazole)(Lb)Cu(η^2^-PhC≡CH)]^+^ (4·Triazole or IV^+^). The computational details and coordinates of all the optimized structures are presented in the SI. All four structures are presented in Fig. 2.

The Lewis-paired complexes I^+^, II and IV^+^, display pseudo-trigonal planar (complexes I^+^ and IV^+^) and trigonal planar (complexes II and III) coordination geometries. Complexes I^+^ and II present a boron-coordinated MesN_3_, through the terminal nitrogen atom, and complex IV^+^ presents a boron-coordinated 1-mesityl-4-phenyl-1*H*-1,2,3-triazole. Although only the terminal coordination of MesN_3_ was computationally considered, other coordination modes of MesN_3_, such as internal azide nitrogen coordination, cannot be ruled out, as attested by the multiple ^11^B NMR resonances experimentally observed in the detection of 1b·N_3_Mes and 2b·N_3_Mes. Complexes I^+^ and IV^+^, similarly to complex 4, have a η^2^-coordinated phenylacetylene ligand, with the Cu–C bond lengths being in the range of 2.005–2.098 Å. On the other hand, the neutral complex II contains a η^1^-coordinated anionic phenylacetylide ligand, displaying a Cu–C bond length of 1.880 Å. In all cases, the B–N bond lengths are in the range of 1.665–1.666 Å. The N_terminal_–N_middle_ bond lengths in MesN_3_ are, on average, *ca.* 0.01 Å longer in the Lewis-paired complexes than in the respective free azide, meaning that no bond reduction takes place upon coordination. This indicates that the Lewis pair formation is reversible. The optimized structure of complex III also features the copper center in a trigonal planar environment, with the Cu–C bond length being 1.926 Å.

**Scheme 4:**
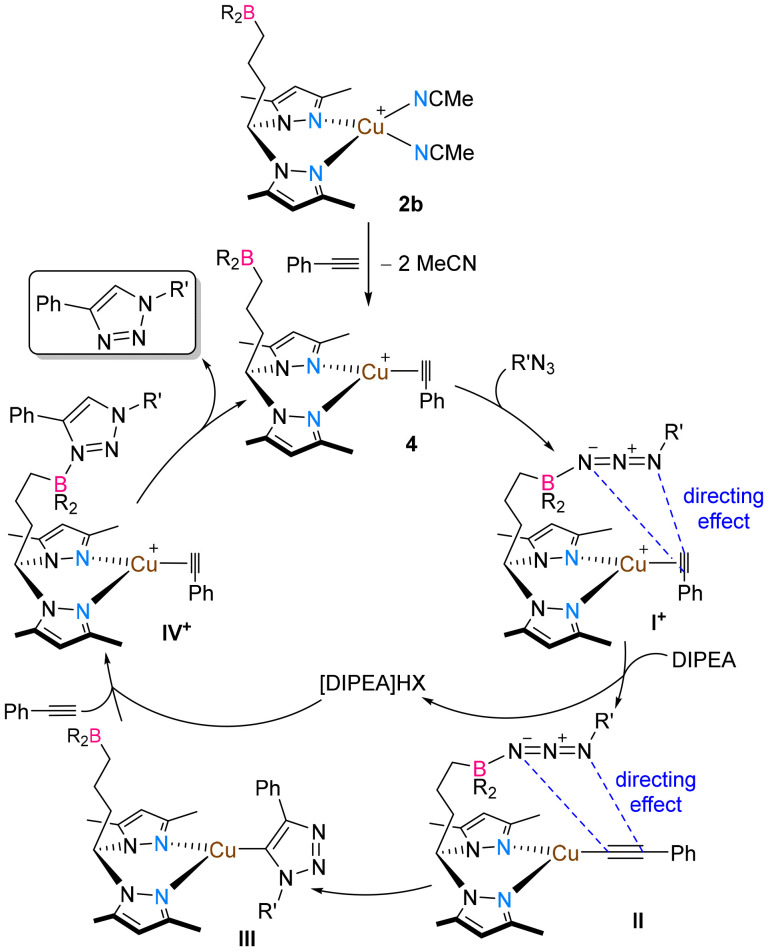
Proposed simplified mechanism for the phenylacetylene-azide cycloaddition reactions catalyzed by complex 2b. BR_2_ denotes 9-BBN and X denotes BF_4_.

Taking the experimental and computational insights into consideration, a simplified mechanism for the CuAAC reactions is presented for complex 2b in Scheme 4.

As presented in Scheme 4, the reaction of complex 2b with phenylacetylene gives rise to complex 4. Reaction of complex 4 with an azide forms the azide Lewis-paired cationic intermediates of the type I^+^, containing a η^2^-coordinated phenylacetylene ligand. Reaction of intermediates I^+^ with DIPEA likely favors the deprotonation of the acetylene ligand, giving rise to the formation of the neutral intermediates II, containing a η^1^-coordinated phenylacetylene ligand, along with the formation of [DIPEA]HBF_4_. Intermediates II, *via* cycloaddition reactions, yield the neutral triazolyl copper intermediates III. The previously formed [DIPEA]HBF_4_ protonates the triazolyl moiety in intermediates III, giving rise to the triazole Lewis-paired intermediate IV^+^, after coordination of phenylacetylene. The coordinative pressure of phenylacetylene eventually forces the dissociation of the triazole product, regenerating complex 4. The present mechanism may also be rationalized for complex 1b, in which case intermediates II are accessed by reaction with DIPEA, phenylacetylene and azide. Azide coordination to copper could not be experimentally ruled out; therefore, intermediates containing copper-coordinated azides may also be involved in the catalytic cycle. This mechanistic proposal is consistent with similar catalytic activities observed for both complexes 1b and 2b. The present mechanism has been previously reported.^9,48^

According to the structural insights obtained from the computational studies, in all complexes containing MesN_3_, it is evident that the azide is pointing toward the face of the copper center, with the azide *ipso* nitrogen being the closest to the copper center, being on average 4.340 Å away. This observation clearly indicates that the borane functionalities in the complexes promote the formation of borane-azide Lewis pairs. The formation of Lewis pairs between the intramolecular borane and the azide substrate allows the azide to be directed onto the acetylene moieties, thus greatly facilitating the subsequent cycloaddition reactions. This effect is likely responsible for the amplified catalytic activity of the borane-functionalized complexes 1b/2b in comparison to the unfunctionalized complexes 1a/2a_2_. An intramolecular borane-induced substrate-directing effect had already been reported in the literature, which was responsible for improving product selectivity in iridium-catalyzed C–H borylation of benzamides and pyridines.^33^

## Conclusions

In the present work, new copper(i) complexes bearing borane-functionalized bis(3,5-dimethylpyrazolyl)methane ligands have been synthesized, characterized and used as precatalysts for the cycloaddition of phenylacetylene and azides.

The copper(i) chloride complexes 1a and 1b were prepared by reaction of the allylated and hydroborated bis(3,5-dimethylpyrazolyl)methane ligands La and Lb, respectively, with CuCl. On the other hand, the reaction of [Cu(NCMe)_4_]BF_4_ with La and Lb, respectively, yielded the cationic copper complexes 2a and 2b. The allylic nature of the ligand in complex 2a rendered it unstable to crystallization, readily forming the binuclear complex 2a_2_. The new complexes were fully characterized by NMR and FTIR spectroscopies, elemental analysis and, in selected cases, by single-crystal X-ray diffraction.

The copper complexes 1a, 1b, 2a_2_ and 2b, in the presence of DIPEA, catalyzed the cycloaddition of phenyl acetylene and *in situ* prepared benzyl azide to the respective 1,2,3-triazole in a maximum yield of 97% and with a maximum TOF value of 800 h^−1^, in 15 minutes at 40 °C. This catalyst system also promoted the cycloaddition of phenylacetylene with 7 *in situ* prepared azides, in product yields of 87–99%. The borane-functionalized complexes were, on average, 4-fold more active than their respective allylated analogues.

Complexes 2a_2_ and 2b reacted with excess phenylacetylene, yielding the homoleptic complex 3a and the heteroleptic η^2^-phenylacetylene complex 4, respectively. The reaction of complexes 1b and 2b with MesN_3_ pointed toward the formation of the Lewis-paired complexes 1b·N_3_Mes and 2b·N_3_Mes. As also evidenced by DFT computational studies, the borane functionalities included in the complexes likely induced a directing effect of the azide substrates, facilitating the azide–alkyne cycloaddition step. As the first example of a CuAAC system that used pendant boranes as a working hypothesis, this extends the proof of concept that including such functionalities in the secondary coordination sphere of a transition metal complex is beneficial to stabilizing catalytic intermediates and amplifying the catalytic activity.

## Conflicts of interest

The authors declare no conflict of interest.

## Supplementary Material

DT-054-D5DT01595B-s001

DT-054-D5DT01595B-s002

DT-054-D5DT01595B-s003

## Data Availability

The data supporting this article have been included as part of the SI: Experimental methodologies; Characterization data for all compounds; Supplementary single-crystal X-ray diffraction data; Supplementary cyclic voltammetry data; Results of the catalytic cycloaddition reactions; Supplementary catalytic information; NMR data of the products of the catalytic reactions; Stoichiometric experiments between complexes 1a, 1b, 2a_2_ and 2b and selected substrates; Atomic coordinates of the optimized geometries. See DOI: https://doi.org/10.1039/d5dt01595b. CCDC 2468989–2468991 contain the supplementary crystallographic data for this paper.^49*a*–*c*^

## References

[cit1] Guo X.-X., Gu D.-W., Wu Z., Zhang W. (2015). Chem. Rev..

[cit2] Zerk T. J., Bernhardt P. V. (2018). Coord. Chem. Rev..

[cit3] Boyer C., Alan N., Kenward C., Diep J., Thuy-Khanh N., Adnan N. N. M., Oliver S., Shanmugam S., Yeow J. (2016). Chem. Rev..

[cit4] Hoover J. M., Stahl S. S. (2011). J. Am. Chem. Soc..

[cit5] Yang Q., Zhao Y., Ma D. (2022). Org. Process Res. Dev..

[cit6] Kolb H. C., Finn M. G., Sharpless K. B. (2001). Angew. Chem., Int. Ed..

[cit7] Haldón E., Nicasio M. C., Pérez P. J. (2015). Org. Biomol. Chem..

[cit8] Tornøe C. W., Christensen C., Meldal M. (2002). J. Org. Chem..

[cit9] Meldal M., Tornøe C. W. (2008). Chem. Rev..

[cit10] Thirumurugan P., Matosiuk D., Jozwiak K. (2013). Chem. Rev..

[cit11] The Nobel Prize in Chemistry 2022. NobelPrize.org. Nobel Prize Outreach AB 2023. https://www.nobelprize.org/prizes/chemistry/2022/summary/

[cit12] Rostovtsev V. V., Green L. G., Fokin V. V., Sharpless K. B. (2002). Angew. Chem., Int. Ed..

[cit13] Fazio F., Bryan M. C., Blixt O., Paulson J. C., Wong C.-H. (2002). J. Am. Chem. Soc..

[cit14] Hein J. E., Fokin V. V. (2010). Chem. Soc. Rev..

[cit15] Díez-González S. (2011). Catal. Sci. Technol..

[cit16] Díez-González S., Stevens E. D., Nolan S. P. (2008). Chem. Commun..

[cit17] Beerhues J., Fauché K., Cisnetti F., Sarkar B., Gautier A. (2019). Dalton Trans..

[a] Ferraro V., Sole R., Bortoluzzi M., Beghetto V., Castro J. (2021). Appl. Organomet. Chem..

[cit19] Parvin N., Hossain J., George A., Parameswaran P., Khan S. (2020). Chem. Commun..

[cit20] Lal S., McNally J., White A. J. P., Díez-González S. (2011). Organometallics.

[cit21] Li L., Lopes P. S., Rosa V., Figueira C. A., Lemos M. A. N. D. A., Duarte M. T., Avilés T., Gomes P. T. (2012). Dalton Trans..

[cit22] Ziegler M. S., Lakshmi K. V., Tilley T. D. (2017). J. Am. Chem. Soc..

[cit23] Stappen C. V., Dai H., Jose A., Tian S., Solomon E. I., Lu Y. (2023). J. Am. Chem. Soc..

[cit24] Drover M. H. (2022). Chem. Soc. Rev..

[cit25] Geri J. B., Shanahan J. P., Szymczak N. K. (2017). J. Am. Chem. Soc..

[cit26] Miller A. J. M., Labinger J. A., Bercaw J. E. (2008). J. Am. Chem. Soc..

[cit27] Ostapowicz T. G., Merkens C., Hölscher M., Klankermayer J., Leitner W. (2013). J. Am. Chem. Soc..

[cit28] Clapson M. L., Drover M. W. (2022). Nat. Synth..

[cit29] Kiernicki J. J., Zeller M., Szymczak N. K. (2017). J. Am. Chem. Soc..

[cit30] Norwine E. E., Kiernicki J. J., Zeller M., Szymczak N. K. (2022). J. Am. Chem. Soc..

[cit31] Song H., Szymczak N. K. (2024). Angew. Chem., Int. Ed..

[cit32] Chugh V., Chatterjee B., Chang W.-C., Cramer H. H., Hindemith C., Randel H., Weyhermüller T., Farès C., Werlé C. (2022). Angew. Chem., Int. Ed..

[cit33] Yang L., Uemura N., Nakao Y. (2019). J. Am. Chem. Soc..

[cit34] Cruz T. F. C., Loupy V., Veiros L. F. (2024). Inorg. Chem..

[cit35] Cruz T. F. C., Veiros L. F. (2025). Dalton Trans..

[cit36] Mahmoud A. G., Martins L. M. D. R. S., Guedes da Silva M. F. C., Pombeiro A. J. L. (2018). Inorg. Chim. Acta.

[cit37] Kramer G. W., Brown H. C. (1974). J. Organomet. Chem..

[cit38] Cremer D., Pople J. A. (1975). J. Am. Chem. Soc..

[cit39] Chou C.-C., Su C.-C., Yeh A. (2005). Inorg. Chem..

[cit40] Yang L., Powell D. R., Houser R. P. (2007). Dalton Trans..

[cit41] Campbell-Verduyn L. S., Mirfeizi L., Dierckx R. A., Elsinga P. H., Feringa B. L. (2009). Chem. Commun..

[cit42] Buckley B. R., Dann S. E., Harris D. P., Heaney H., Stubbs E. C. (2010). Chem. Commun..

[cit43] Worrell B. T., Malik J. A., Fokin V. V. (2013). Science.

[cit44] Akl D. F., Poier D., D'Angelo S., Araújo T. P., Tulus V., Safonova O. V., Mitchell S., Marti R., Guillén-Gosálbez G., Pérez-Ramírez J. (2022). Green Chem..

[cit45] Pavia D. L. , Lampman G. M., Kriz G. S. and Vyvyan J. R., Introduction to Spectroscopy, Cengage Learning, 5th edn, 2004

[cit46] Cenini S., Gallo E., Caselli A., Ragaini F., Fantauzzi S., Piangiolino C. (2006). Coord. Chem. Rev..

[cit47] Parr R. G. and Yang W., Density Functional Theory of Atoms and Molecules, Oxford University Press, New York, 1989

[cit48] Mahmoud A. G., Guedes da Silva M. F. C., Mahmudov K. T., Pombeiro A. J. L. (2019). Dalton Trans..

[cit49] (a) Cruz T. F. C. López-Sánchez B. , Lemos M. A. N. D. A., Romerosa A. and Martins L. M. D. R. S., CCDC 2468989: Experimental Crystal Structure Determination, 2025, 10.5517/ccdc.csd.cc2nw5tk

