# Supplementary material for: Borane-functionalized heteroscorpionate copper complexes as catalysts for azide–alkyne cycloaddition
Source: Dalton Trans. 2025 Aug 13;54(36):13472–82. doi: 10.1039/d5dt01595b (PMC12394796; doi:10.1039/d5dt01595b)
Supplement: DT-054-D5DT01595B-s001 [file DT-054-D5DT01595B-s001.pdf]

Supplementary Information (SI) for Dalton Transactions.
This journal is © The Royal Society of Chemistry 2025

Supplementary Information for

Borane-functionalized heteroscorpionate copper complexes as catalysts for azide-alkyne cycloaddition

Tiago F. C. Cruz,[1,2,*] Belén López-Sánchez,[1,3] M. Amélia N. D. A. Lemos,[4] Antonio Romerosa[3] and Luísa M. D. R. S. Martins[1,*]

[1]*Centro de Química Estrutural, Departamento de Engenharia Química, Instituto Superior Técnico, Universidade de Lisboa, Av. Rovisco Pais 1, 1000-049 Lisboa, Portugal.*
[2]*Institute of Applied Synthetic Chemistry, TU Wien, Getreidemarkt 9/163-AC, A-1060 Wien, Austria.*
[3]*Area de Química Inorgánica-CIESOL, Universidad de Almería, Almería, Spain.*
[4]*CERENA, Departamento de Engenharia Química, Instituto Superior Técnico, Universidade de Lisboa, Av. Rovisco Pais 1, 1000-049 Lisboa, Portugal.*

*Corresponding Authors; e-mails: carpinteirocruz@tecnico.ulisboa.pt
luisammartins@tecnico.ulisboa.pt

Table of contents

Table of contents S1
Experimental methodologies S2
Characterization data for all compounds S10
Supplementary single-crystal X-ray diffraction data S31
Supplementary cyclic voltammetry data S34
Results of the catalytic cycloaddition reactions S35
Supplementary catalytic information S38
NMR data of the products of the catalytic reactions S39
Stoichiometric experiments between complexes **1a**, **1b**, **2a$_2$** and **2b** and selected substrates S43
Atomic coordinates of the optimized geometries S47
References S56

Experimental methodologies

General procedures: All operations were performed under dry dinitrogen atmosphere using standard glovebox and Schlenk techniques unless otherwise noted. Dinitrogen gas used for all operations (purity: <1 ppm O_2 and H_2O) was supplied by Air Liquide and further purified by passage through 4 Å molecular sieves. Solvents were pre-dried over activated 4 Å molecular sieves and distilled by heating under dinitrogen over suitable drying agents (sodium/benzophenone for diethyl ether; CaH_2 for *n*-hexane and dichloromethane). Deuterated solvents used were dried over activated 4 Å molecular sieves and degassed by the freeze-pump-thaw technique. Solvents and solutions were transferred using positive pressure of dinitrogen through stainless steel cannulae and mixtures were filtered in a similar way using modified cannulae that could be fitted with glass fiber filter disks. Elemental analyses were obtained from the elemental analysis service of Instituto Superior Técnico (IST) on a Fisons Instrument Mod EA-1108. Phenylacetylene and diisopropylethylamine (DIPEA) were dried over CaH_2 and distilled under reduced pressure. The remaining reagents were used as received from commercial sources (*e.g.* Acros, Alfa Aesar). Mesitylazide,[1] $[Cu(NCMe)_4]BF_4$,[2] 1,1'-(but-3-ene-1,1-diyl)*bis*(3,5-dimethylpyrazolyl) (**La**)[3] and (9-borabicyclo[3.3.1]nonan-9-yl)butane-1,1-diyl)*bis*(3,5-dimethylpyrazolyl) (**Lb**)[3] were prepared as described in the literature.

NMR spectroscopy measurements: NMR spectra were recorded on a *Bruker "AVANCE III"* 300 MHz spectrometer at 299.995 MHz (^{1}H), 75.4296 MHz (^{13}C), 96.2712 MHz (^{11}B), and 282.404 (^{19}F). The spectra were referenced internally using the residual protio-resonances (^{1}H) and the solvent carbon (^{13}C) resonances of the corresponding solvents[4] to tetramethylsilane ($\delta = 0$), and referenced externally using 15% $BF_3{\cdot}OEt_2$ ($\delta = 0$), for ^{11}B, and $CFCl_3$ ($\delta = 0$) for ^{19}F. All solution samples, excluding those involving organic molecules devoid of boranes, were prepared inside a glovebox and transferred to screw-capped or J. Young NMR tubes. All chemical shifts are quoted in δ (ppm) and coupling constants (*J*) in Hz with multiplicities abbreviated as br (broad), s (singlet), d (doublet), t (triplet), q (quartet), h (heptet) and m (multiplet).

X-ray diffraction: Crystallographic and experimental details of crystal structure determinations are listed in Tables S1 and S2 of the ESI. The crystals were selected under an inert atmosphere, covered with polyfluoroether oil and mounted on a nylon loop.

Crystallographic data were collected using graphite monochromated Mo-Kα radiation (λ = 0.71073 Å) on a Bruker AXS-KAPPA APEX II diffractometer equipped with an Oxford Cryosystem open-flow dinitrogen cryostat, at 150 K. Cell parameters were retrieved using Bruker SMART[5] software and refined using Bruker SAINT[6] on all observed reflections. Absorption corrections were applied using SADABS.[7] Structure solution and refinement were performed using direct methods with the programs SIR2014[8] and SHELXL[9] included in the package of programs WINGX-Version 2014.1.[10] All non-hydrogen atoms were refined anisotropically, and the hydrogen atoms were inserted in idealized positions and allowed to refine riding on the parent carbon atom.

The CIF file corresponding to the molecular structure of complex **2a$_2$** presented one B-level alert, which was associated with a moderate R_{int} value (0.205), likely due to the poor diffracting power/lack of unique data of the crystal. The CIF file corresponding to the molecular structure of complex **3a** presented several A-level alerts, likely associated with crystal centering or poor crystal quality. Despite recollection attempts, obtaining better quality data was not possible. Nevertheless, it was possible to undoubtedly solve their molecular structure. For this reason, the molecular structure of complex **3a** was only presented as proof of its molecular connectivity in Figure S37. The tetrafluoroborate anions in the structures of **2a$_2$** and **3a** and half of a 3,5-dimethylpyrazolyl ligand in **3a** presented positional disorder. Even though the inclusion of disorder models was attempted, no satisfactory results were obtained, prompting us to not include them in the final structure solution.

Graphic presentations were prepared with Mercury 2022.3.0.[11] Data was deposited in CCDC under the codes 2468989 for **1a**, 2468990 for **2a$_2$** and 2468991 for **3a**.

Cyclic voltammetry measurements: Cyclic voltammetry experiments on *ca.* 2 to 4 mM solutions of the complexes **1a**, **1b**, **2a$_2$**, **2b**, **3a** and **4** in dichloromethane, using $[N(n\text{-Bu})_4]BF_4$ (0.2 M) as a supporting electrolyte, were performed at a scanning rate of 200 mV s^{-1} (unless otherwise specified) with a three compartment electrochemical cell, under dinitrogen atmosphere, at room temperature, using a Pt disc working electrode and a Pt wire counter electrode, with a Ag pseudo-reference electrode connected to the main compartment by a Luggin capillary. The redox potentials were calculated using the reference potential of the ferrocene/ferrocenium couple measured under the same experimental conditions for each of the complexes.

Computational details: All calculations were performed using the Gaussian 09 software package[12] and the PBE0 functional. This functional uses a hybrid generalized gradient approximation (GGA), including 25% mixture of Hartree-Fock[13] exchange with DFT exchange-correlation, given by Perdew, Burke and Ernzerhof functional (PBE),[14] and has proven suitable for closed-shell systems, as shown in a previous publication using zinc as the metal and the same ligand systems.[3] The geometry optimizations were accomplished without symmetry constraints using a standard 6-31G** basis set[15] for all atoms except for copper, that used the SDD[16] basis set with a *f*-polarization function[17] for Cu (basis b1).

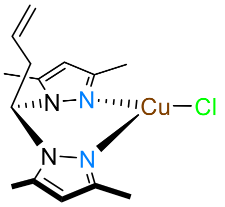

Synthesis of [(La)CuCl] (1a): Dichloromethane was added to a solid mixture of **La** (1.5 mmol, 0.37 g) and CuCl (1.6 mmol, 0.16 g) and the resulting cloudy solution was stirred at room temperature for 16 h. All volatiles were removed under reduced pressure, the residue redissolved in minimal dichloromethane and the solution was filtered onto three-fold *n*-hexane under magnetic stirring, thus precipitating a white powder. The supernatant was filtered off and the white powder was dried under vacuum. Crystals suitable for X-ray diffraction were obtained from slow evaporation of a saturated dichloromethane solution into *n*-hexane. Yield: 0.49 g (93%). Anal. Calc. for $C_{14}H_{20}ClCuN_4$, obtained (calculated): C 48.81 (48.98), H 5.78 (5.87), N 16.04 (16.32). ^{1}H NMR (300 MHz, $CDCl_3$): δ 6.00 (1H, t, NC*H*N, $^{3}J_{HH}$ = 9.0 Hz), 5.87 (2H, s, C$H_{pyrazolyl}$), 5.30 (1H, m, =CH_{allyl}), 5.08 (2H, m, =CH_{2allyl}), 3.28 (2H, t, CH_{2allyl}, $^{3}J_{HH}$ = 9.0 Hz), 2.39 (6H, s, C$H_{3pyrazolyl}$), 2.35 (6H, s, C$H_{3pyrazolyl}$). ^{13}C{^{1}H} NMR (75 MHz, $CDCl_3$): δ 151.6 (*C*$Me_{pyrazolyl}$), 139.8 (*C*$Me_{pyrazolyl}$), 129.3 (=CH_{allyl}), 120.3 (=CH_{2allyl}), 106.3 ($CH_{pyrazolyl}$), 66.2 (N*C*HN), 40.7 (CH_{2allyl}), 14.0 (C$H_{3pyrazolyl}$), 11.3 (C$H_{3pyrazolyl}$).

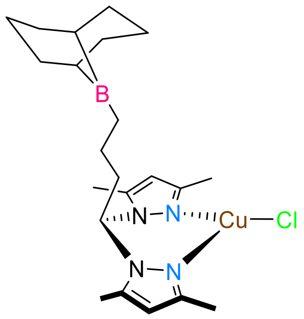

Synthesis of [(Lb)CuCl] (1b): Dichloromethane was added to a solid mixture of **Lb** (0.46 mmol, 0.17 g) and CuCl (0.5 mmol, 0.05 g) and the resulting solution was stirred at room temperature for 16 h. All volatiles were removed under reduced pressure, the residue redissolved in minimal dichloromethane and the solution was filtered onto three-fold *n*-hexane under magnetic stirring, thus precipitating a white powder. The supernatant was filtered off and the white powder was dried under vacuum. Yield: 0.17 g (80%). Anal. Calc. for $C_{22}H_{35}BClCuN_4$•$0.33CH_2Cl_2$, obtained (calculated): C 54.02 (54.36), H 7.85 (7.29), N 11.14 (11.36). ^{1}H NMR (300 MHz, $CDCl_3$): δ 5.99 (1H, t, NC*H*N, $^3J_{HH}$ = 9.0 Hz), 5.89 (2H, s, C$H_{pyrazolyl}$), 2.58 (2H, m, B-$CH_2CH_2CH_2$-C), 2.42 (6H, s, C$H_{3pyrazolyl}$), 2.36 (6H, s, C$H_{3pyrazolyl}$), 1.88-1.74 (6H, m, C$H_{2,9\text{-BBN}}$), 1.66-1.53 (6H, m, C$H_{2,9\text{-BBN}}$), 1.40-1.17 (6H, m, C$H_{9\text{-BBN}}$ + B-C$H_2CH_2CH_2$-C + B-$CH_2CH_2CH_2$-C). ^{13}C{^{1}H} NMR (75 MHz, $CDCl_3$): δ 151.6 ($CMe_{pyrazolyl}$), 139.3 ($CMe_{pyrazolyl}$), 106.3 ($CH_{pyrazolyl}$), 66.5 (NCHN), 39.8 ($CH_{2,9\text{-BBN}}$), 33.3 ($CH_{2,9\text{-BBN}}$), 31.1 ($CH_{9\text{-BBN}}$), 23.2 ($CH_{2,9\text{-BBN}}$), 20.3 ($CH_{2,9\text{-BBN}}$), 14.1 ($CH_{3pyrazolyl}$), 11.2 ($CH_{3pyrazolyl}$). ^{11}B NMR (96 MHz, $CDCl_3$): δ 87.5 (br, 9-BBN).

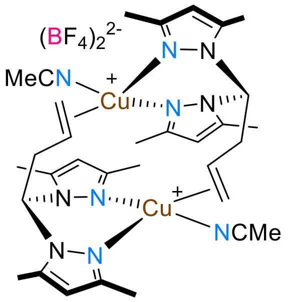

Synthesis of [(La)Cu(NCMe)]$_2$(BF$_4$)$_2$ (2a$_2$): Dichloromethane was added to a solid mixture of **La** (0.5 mmol, 0.12 g) and [Cu(NCMe)$_4$]BF$_4$ (0.5 mmol, 0.16 g) and the resulting solution was stirred at room temperature for 16 h. All volatiles were removed under reduced pressure, the residue redissolved in minimal dichloromethane and the solution was filtered onto three-fold *n*-hexane under magnetic stirring, thus precipitating a white powder. The supernatant was filtered off and the white powder was dried under vacuum. Crystals suitable for X-ray diffraction were obtained from vapor diffusion of diethyl ether into a concentrated acetonitrile

solution. Yield: 0.16 g (73%). Anal. Calc. for $C_{16}H_{23}BCuF_4N_5$, obtained (calculated): C 44.47 (44.10), H 5.12 (5.32), N 15.23 (16.07). ^{1}H NMR (300 MHz, CD_3CN): δ 6.24 (2H, br, NC*H*N), 5.98 (4H, s, C*H*$_{pyrazolyl}$), 5.47 (2H, m, =C*H*$_{allyl}$), 5.02 (4H, m, =C*H*$_{2allyl}$), 3.18 (4H, br, C*H*$_{2allyl}$), 2.36 (12H, s, C*H*$_{3pyrazolyl}$), 2.25 (12H, s, C*H*$_{3pyrazolyl}$), 1.96 (6H, s, C*H*$_3$CN). ^{13}C{^{1}H} NMR (75 MHz, CD_3CN): δ 151.4 (*C*Me$_{pyrazolyl}$), 142.6 (*C*Me$_{pyrazolyl}$), 130.8 (=C*H*$_{allyl}$), 120.1 (=*C*H$_{2allyl}$), 106.6 (*C*H$_{pyrazolyl}$), 66.7 (N*C*HN), 40.1 (C*H*$_{2allyl}$), 14.0 (C*H*$_{3pyrazolyl}$), 11.3 (C*H*$_{3pyrazolyl}$), 1.9 (*C*H$_3$CN). CH$_3$*C*N resonance absent. ^{11}B NMR (96 MHz, CD_3CN): δ -1.2 (*B*F$_4^-$). ^{19}F{^{1}H} (282 MHz, CD_3CN): δ -151.7 (B*F*$_4^-$).

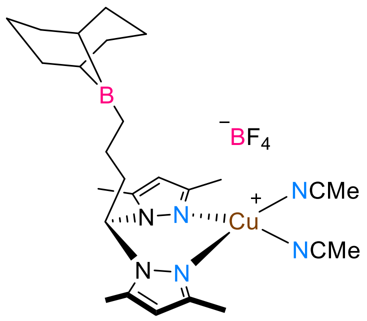

Synthesis of [(Lb)Cu(NCMe)$_2$]BF$_4$ (2b): Dichloromethane was added to a solid mixture of **Lb** (0.5 mmol, 0.17 g) and [Cu(NCMe)$_4$]BF$_4$ (0.5 mmol, 0.16 g) and the resulting solution was stirred at room temperature for 16 h. All volatiles were removed under reduced pressure, the residue redissolved in minimal dichloromethane and the solution was filtered onto three-fold *n*-hexane under magnetic stirring, thus precipitating a white powder. The supernatant was filtered off and the white powder was dried under vacuum. Yield: 0.17 g (57%). Anal. Calc. for $C_{26}H_{41}B_2CuF_4N_6$•0.75CH_2Cl_2, obtained (calculated): C 48.82 (48.50), H 6.93 (6.47), N 11.75 (12.69). ^{1}H NMR (300 MHz, $CDCl_3$): δ 6.23 (1H, t, NC*H*N, $^3J_{HH}$ = 6.0 Hz), 5.94 (2H, s, C*H*$_{pyrazolyl}$), 2.47-2.23 (20H, m, B-CH$_2$C*H*$_2$CH$_2$-C + C*H*$_{3pyrazolyl}$ + C*H*$_3$CN), 1.88-1.74 (6H, m, C*H*$_{2,9\text{-BBN}}$), 1.67-1.50 (6H, m, C*H*$_{2,9\text{-BBN}}$), 1.32 (2H, m, B-C*H*$_2$CH$_2$CH$_2$-C), 1.26-1.13 (2H, m, C*H*$_{9\text{-BBN}}$ + B-CH$_2$CH$_2$C*H*$_2$-C). ^{13}C{^{1}H} NMR (75 MHz, $CDCl_3$): δ 151.4 (*C*Me$_{pyrazolyl}$), 142.0 (*C*Me$_{pyrazolyl}$), 118.03 (CH$_3$*C*N), 106.3 (*C*H$_{pyrazolyl}$), 66.6 (N*C*HN), 40.4 (*C*H$_{2,9\text{-BBN}}$), 33.1 (*C*H$_{2,9\text{-BBN}}$), 30.6 (*C*H$_{9\text{-BBN}}$), 23.2 (*C*H$_{2,9\text{-BBN}}$), 19.7 (*C*H$_{2,9\text{-BBN}}$), 13.9 (C*H*$_{3pyrazolyl}$), 11.0 (C*H*$_{3pyrazolyl}$), 2.50 (*C*H$_3$CN). ^{11}B NMR (96 MHz, $CDCl_3$): δ 86.2 (br, 9-BBN), -0.9 (*B*F$_4^-$). ^{19}F{^{1}H} (282 MHz, $CDCl_3$): δ -153.2 (B*F*$_4^-$).

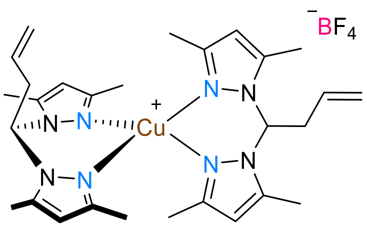

Synthesis of $[(La)_2Cu]BF_4$ (3a): Phenylacetylene (2.94 mmol, 0.30 g) was added to a suspension of complex **$2a_2$** (0.11 mmol, 0.10 g) in dichloromethane and the mixture was stirred for 16 h. All volatile materials were evaporated to dryness and the white powder was dried under vacuum. Yield: 0.065 g (46%), based on Cu. *Alternative method*: Dichloromethane was added to a solid mixture of **La** (1.0 mmol, 0.24 g) and $[Cu(NCMe)_4]BF_4$ (0.5 mmol, 0.16 g) and the resulting solution was stirred at room temperature for 16 h. All volatiles were removed under reduced pressure, the residue redissolved in minimal dichloromethane and the solution was filtered onto three-fold *n*-hexane under magnetic stirring, thus precipitating a white powder. The supernatant was filtered off and the white powder was dried under vacuum. Yield: 0.27 g (85%). Crystals suitable for X-ray diffraction were obtained from vapor diffusion of diethyl ether into a concentrated acetonitrile solution. Anal. Calc. for $C_{28}H_{40}BCuF_4N_8$, obtained (calculated): C 52.40 (52.63), H 6.53 (6.31), N 17.58 (17.54). ^{1}H NMR (300 MHz, $CDCl_3$): δ 6.30 (2H, t, NC*H*N, $^3J_{HH}$ = 6.0 Hz), 5.83 (4H, s, C$H_{pyrazolyl}$), 5.47 (2H, m, =CH_{allyl}), 5.07 (4H, m, =CH_{2allyl}), 3.42 (4H, br, CH_{2allyl}), 2.43 (12H, s, C$H_{3pyrazolyl}$), 2.07 (6H, vbr s, C$H_{3pyrazolyl}$), 1.21 (6H, vbr s, C$H_{3pyrazolyl}$). ^{13}C{^{1}H} NMR (75 MHz, $CDCl_3$): δ 150.4 (*C*$Me_{pyrazolyl}$), 141.0 (*C*$Me_{pyrazolyl}$), 130.9 (=CH_{allyl}), 120.5 (=*C*H_{2allyl}), 106.3 (*C*$H_{pyrazolyl}$), 66.4 (N*C*HN), 40.0 (CH_{2allyl}), 11.3 (C$H_{3pyrazolyl}$). ^{11}B NMR (96 MHz, $CDCl_3$): δ -0.8 (*B*F_4^-). ^{19}F{^{1}H} (282 MHz, $CDCl_3$): δ -153.1 (BF_4^-).

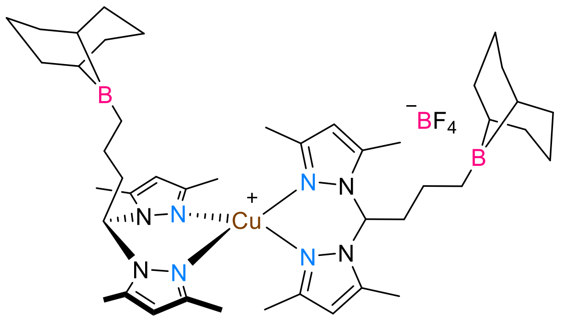

Synthesis of $[(Lb)_2Cu]BF_4$ (3b): Dichloromethane was added to a solid mixture of **Lb** (0.64 mmol, 0.23 g) and $[Cu(NCMe)_4]BF_4$ (0.32 mmol, 0.10 g) and the resulting solution was stirred at room temperature for 16 h. All volatiles were removed under reduced pressure, the residue redissolved in minimal dichloromethane and the solution was filtered onto three-fold *n*-hexane under magnetic stirring, thus precipitating a white powder. The supernatant was filtered off and

the white powder was dried under vacuum. Yield: 0.24 g (86%). Anal. Calc. for $C_{44}H_{70}B_3CuF_4N_8$•$2.5CH_2Cl_2$, obtained (calculated): C 51.29 (50.99), H 6.46 (6.90), N 10.68 (10.23). ^{1}H NMR (300 MHz, $CDCl_3$): δ 6.26 (2H, t, NC*H*N, $^{3}J_{HH}$ = 9.0 Hz), 5.85 (4H, s, C*H*$_{pyrazolyl}$), 2.72 (4H, m, B-CH_2C*H*$_2$$CH_2$-C), 2.45 (12H, s, C*H*$_{3pyrazolyl}$), 2.16 (6H, v br s, C*H*$_{3pyrazolyl}$), 2.07 (6H, s, C*H*$_{3pyrazolyl}$), 1.90-1.20 (36H, m, C*H*$_{2,9\text{-BBN}}$ + C*H*$_{9\text{-BBN}}$ + B-C*H*$_2$$CH_2$$CH_2$-C + B-$CH_2$$CH_2$C*H*$_2$-C). ^{13}C{^{1}H} NMR (75 MHz, $CDCl_3$): δ 149.9 (*C*Me$_{pyrazolyl}$), 140.6 (*C*Me$_{pyrazolyl}$), 106.3 (*C*H$_{pyrazolyl}$), 66.8 (N*C*HN), 39.1 (*C*H$_{2,9\text{-BBN}}$), 33.1 (*C*H$_{2,9\text{-BBN}}$), 30.5 (*C*H$_{9\text{-BBN}}$), 23.4 (*C*H$_{2,9\text{-BBN}}$), 20.1 (*C*H$_{2,9\text{-BBN}}$). ^{11}B NMR (96 MHz, $CDCl_3$): δ 80.6 (br, 9-BBN), -0.9 (*B*F_4^-). ^{19}F{^{1}H} (282 MHz, $CDCl_3$): δ -153.6 (B*F*$_4^-$).

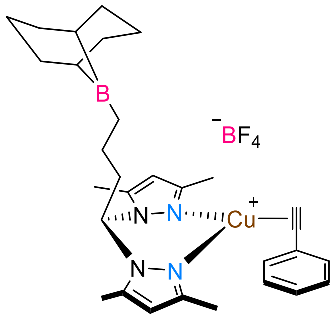

Synthesis of [(Lb)Cu(η^2-PhC≡CH)]BF_4 (4): Phenylacetylene (2.94 mmol, 0.30 g) was added to a solution of complex **2b** (0.17 mmol, 0.10 g) in dichloromethane and the mixture was stirred for 16 h. All volatile materials were evaporated to dryness, the residue redissolved in minimal dichloromethane, the solution filtered onto three-fold *n*-hexane under magnetic stirring, thus precipitating a tan powder, which was dried under vacuum. Yield: 0.095 g (90%). Anal. Calc. for $C_{30}H_{41}B_2CuF_4N_4$•$0.25CH_2Cl_2$, obtained (calculated): C 56.92 (56.76), H 7.23 (6.54), N 8.49 (8.75). ^{1}H NMR (300 MHz, $CDCl_3$): δ 7.55-7.31 (5H, m, *Ph*C≡CH), 6.65 (1H, t, NC*H*N, $^{3}J_{HH}$ = 9.0 Hz), 5.96 (2H, s, C*H*$_{pyrazolyl}$), 5.29 (1H, s, PhC≡C*H*), 2.73 (2H, q, B-CH_2C*H*$_2$$CH_2$-C, $^{3}J_{HH}$ = 6.0 Hz), 2.52 (6H, s, C*H*$_{3pyrazolyl}$), 2.02 (6H, s, C*H*$_{3pyrazolyl}$), 1.84-1.38 (16H, m, C*H*$_{2,9\text{-BBN}}$ + C*H*$_{9\text{-BBN}}$ B-C*H*$_2$$CH_2$$CH_2$-C), 1.15 (2H, m, B-$CH_2$$CH_2$C*H*$_2$-C). ^{13}C{^{1}H} NMR (75 MHz, $CDCl_3$): δ 151.7 (*C*Me$_{pyrazolyl}$), 143.6 (*C*Me$_{pyrazolyl}$), 131.7 (*Ph*C≡CH), 130.1 (*Ph*C≡CH), 129.0 (*Ph*C≡CH), 107.2 (*C*H$_{pyrazolyl}$), 94.9 (Ph*C*≡CH), 81.1 (PhC≡*C*H), 40.8 (*C*H$_{2,9\text{-BBN}}$), 33.2 (*C*H$_{2,9\text{-BBN}}$), 30.9 (*C*H$_{9\text{-BBN}}$), 23.3 (*C*H$_{2,9\text{-BBN}}$), 19.7 (*C*H$_{2,9\text{-BBN}}$), 13.9 (C*H*$_{3pyrazolyl}$), 11.3 (C*H*$_{3pyrazolyl}$). ^{11}B NMR (96 MHz, $CDCl_3$): δ 87.8 (br, 9-BBN), -0.8 (*B*F_4^-). ^{19}F{^{1}H} (282 MHz, $CDCl_3$): δ -152.2 (B*F*$_4^-$).

General procedure for the catalytic cycloaddition of azides and phenylacetylene in several conditions: A mixture of the desired benzyl or aryl bromide (0.33 mmol), NaN_3 (0.33 mmol) and the required amount of phenylacetylene (0.33 mmol, 0.4125 mmol or 0.495 mmol) was prepared in dichloromethane (0.4 mL). To this mixture, the required amount of complex (0.0033 mmol, 0.004125 mmol or 0.00495 mmol) and diisopropylethylamine (DIPEA) was added. The solution was stirred at the specified temperature and time. After the designated time, reactions cooled to room temperature, if required, and filtered the volatile materials were carefully evaporated under vacuum. The products were extracted with $CDCl_3$ (3×2 mL). The resulting product was analyzed by ^1H NMR spectroscopy, thereby allowing the determination of the respecting conversion rates. The identity of the resulting triazole compounds was assessed by comparison with literature data.[18] The optimization data for the reactions catalyzed by complexes **1a,b**, **2a$_2$** and **2b** are presented in Tables S4-S7.

Characterization data for all compounds

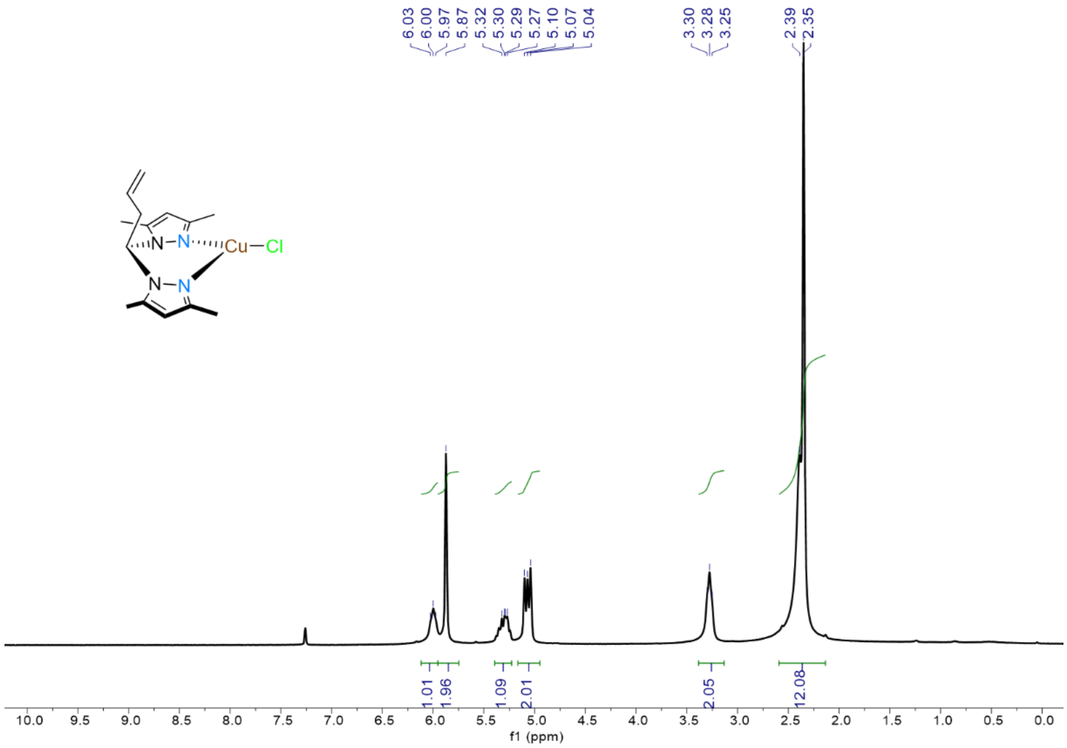

Figure S1 ^{1}H NMR spectrum (300 MHz, $CDCl_3$) of complex **1a**.

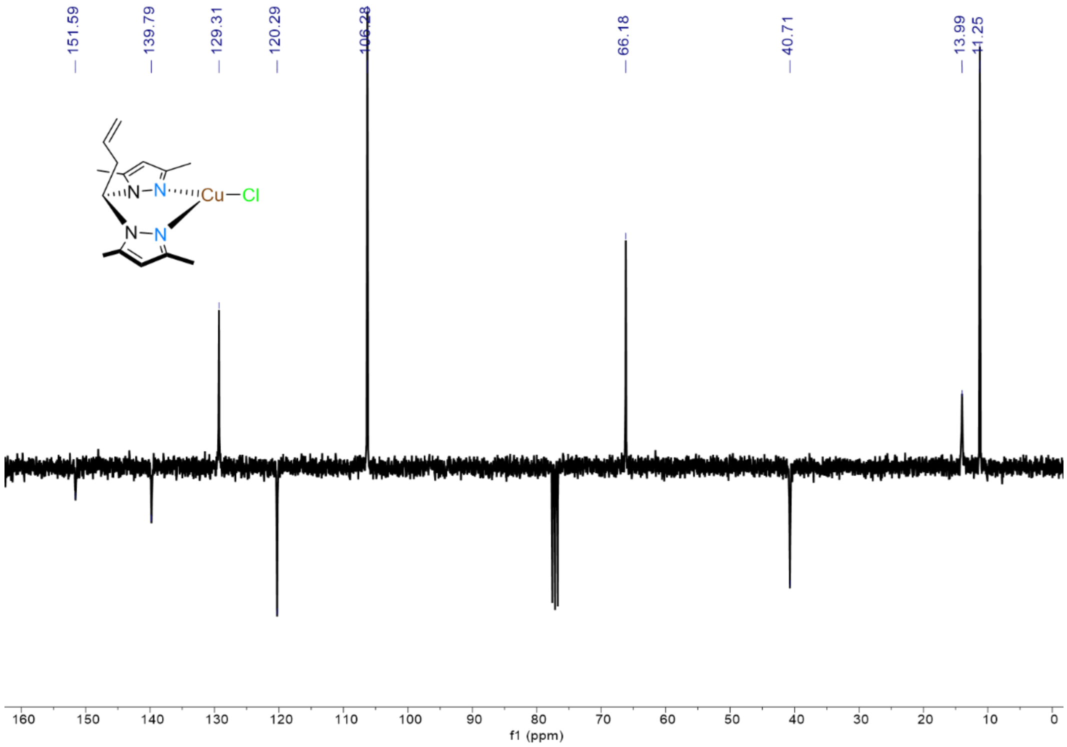

Figure S2 ^{13}C APT NMR spectrum (75 MHz, $CDCl_3$) of complex **1a**.

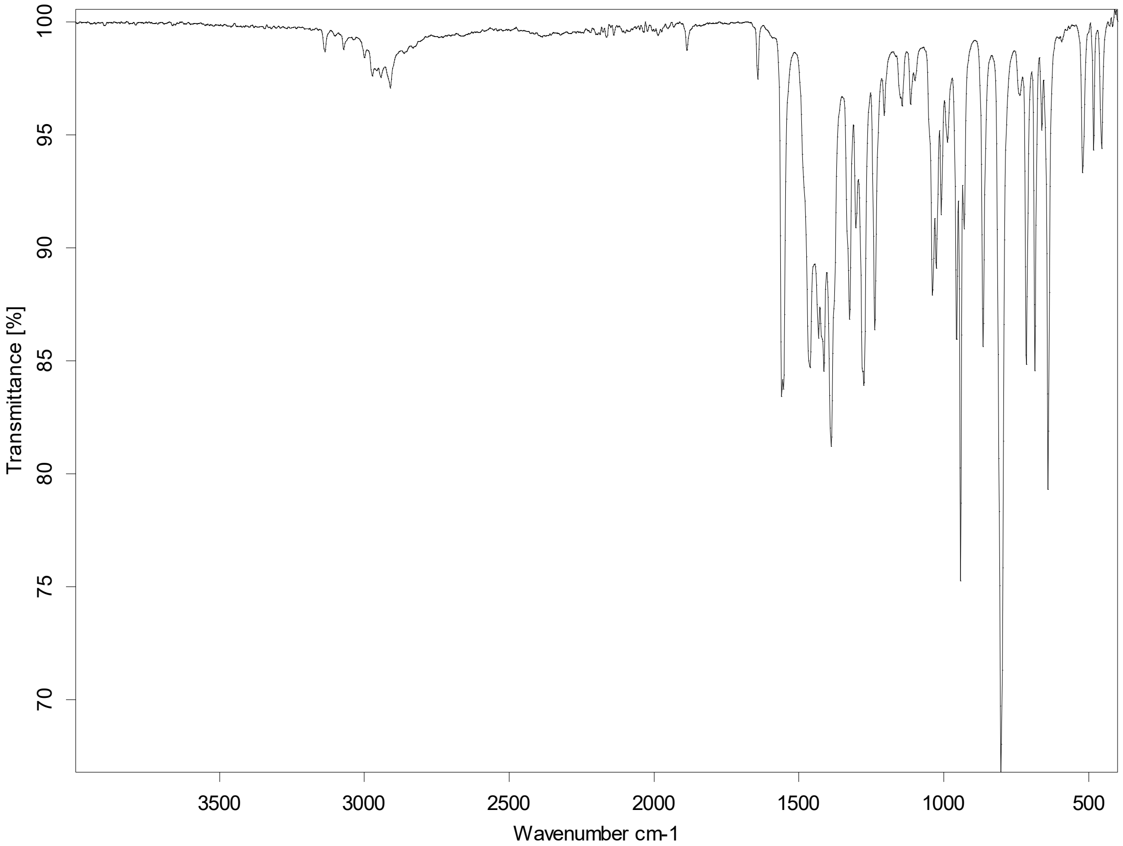

Figure S3 ATR-FTIR spectrum of complex **1a**.

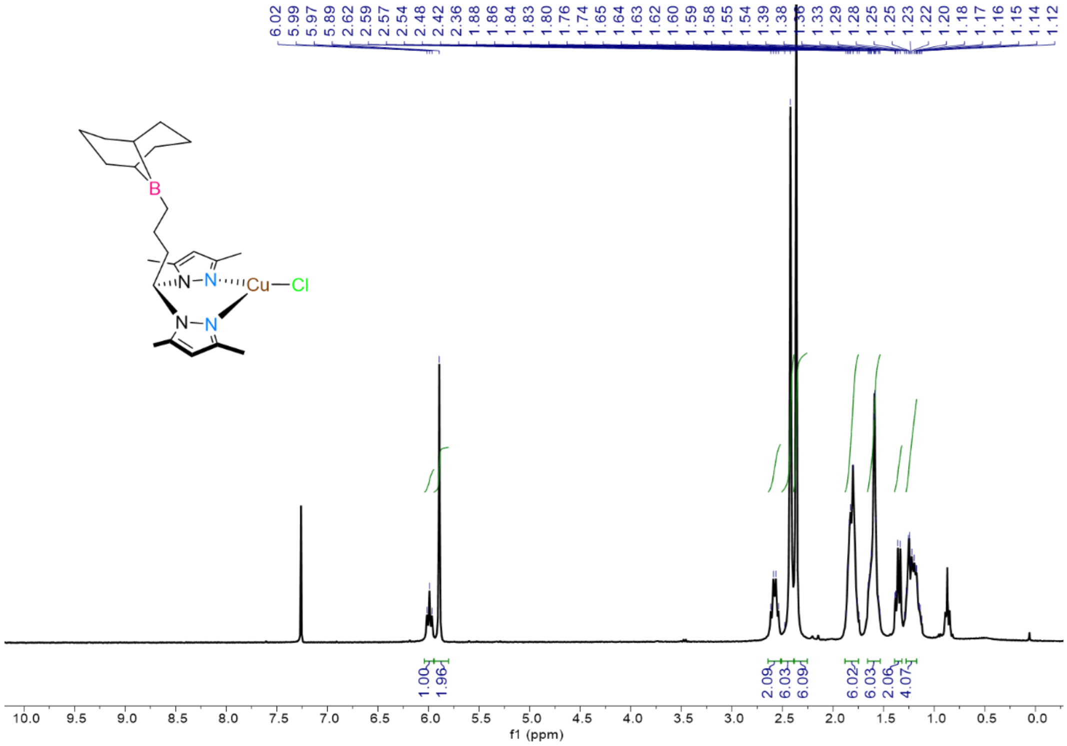

Figure S4 ^{1}H NMR spectrum (300 MHz, $CDCl_3$) of complex **1b**.

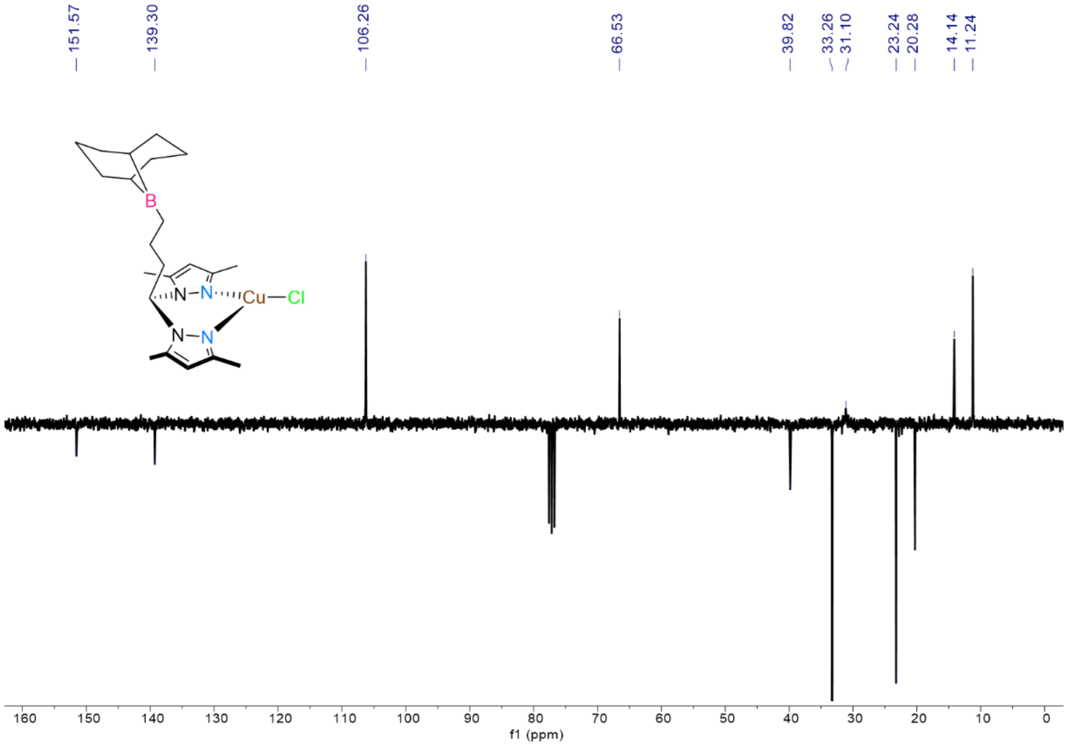

Figure S5 ^{13}C APT NMR spectrum (75 MHz, $CDCl_3$) of complex **1b**.

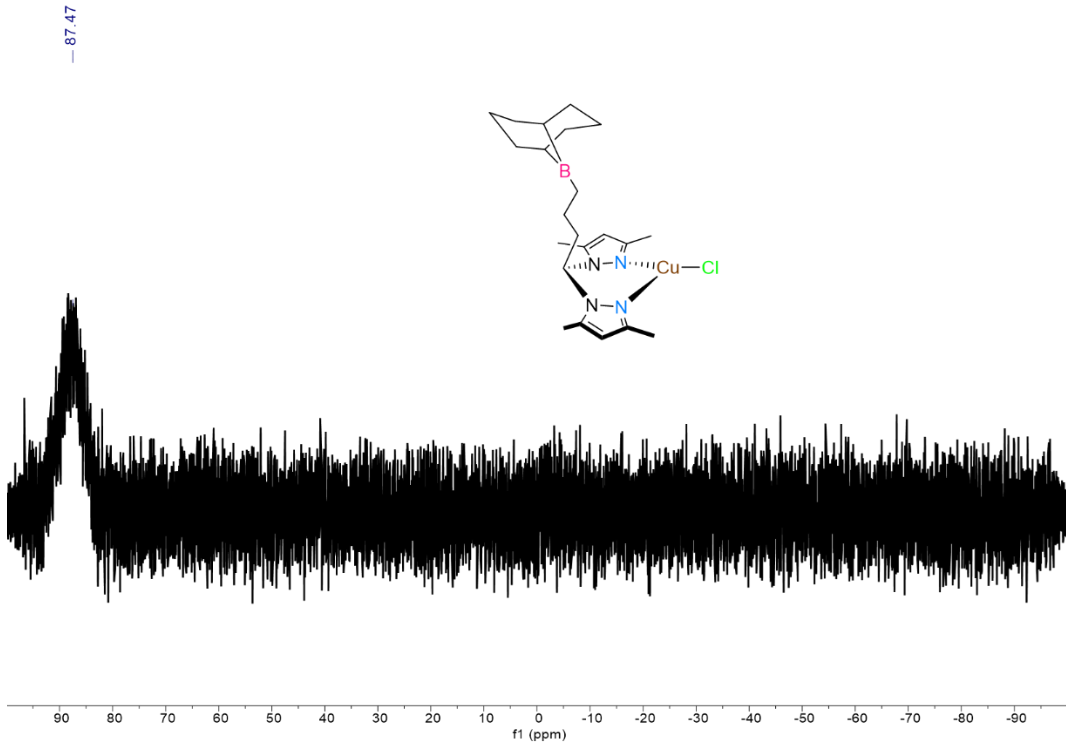

Figure S6 ^{11}B NMR spectrum (96 MHz, $CDCl_3$) of complex **1b**.

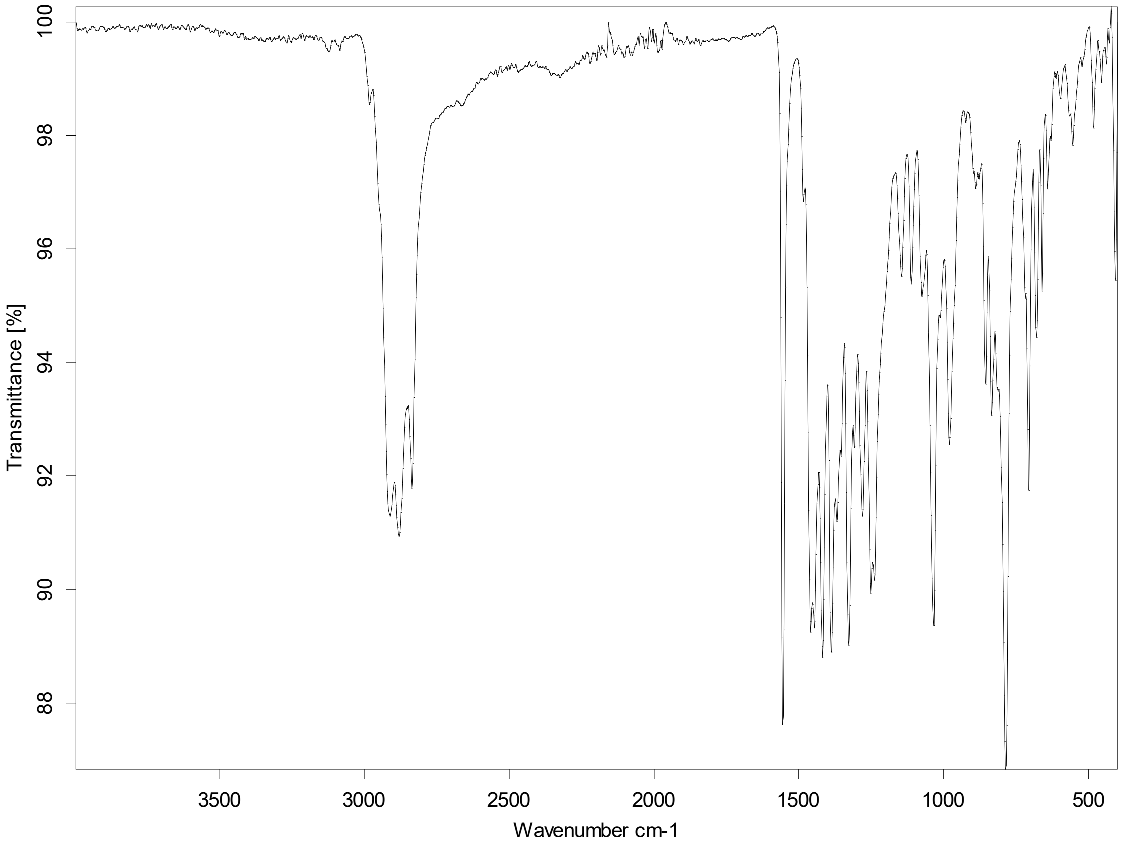

Figure S7 ATR-FTIR spectrum of complex **1b**.

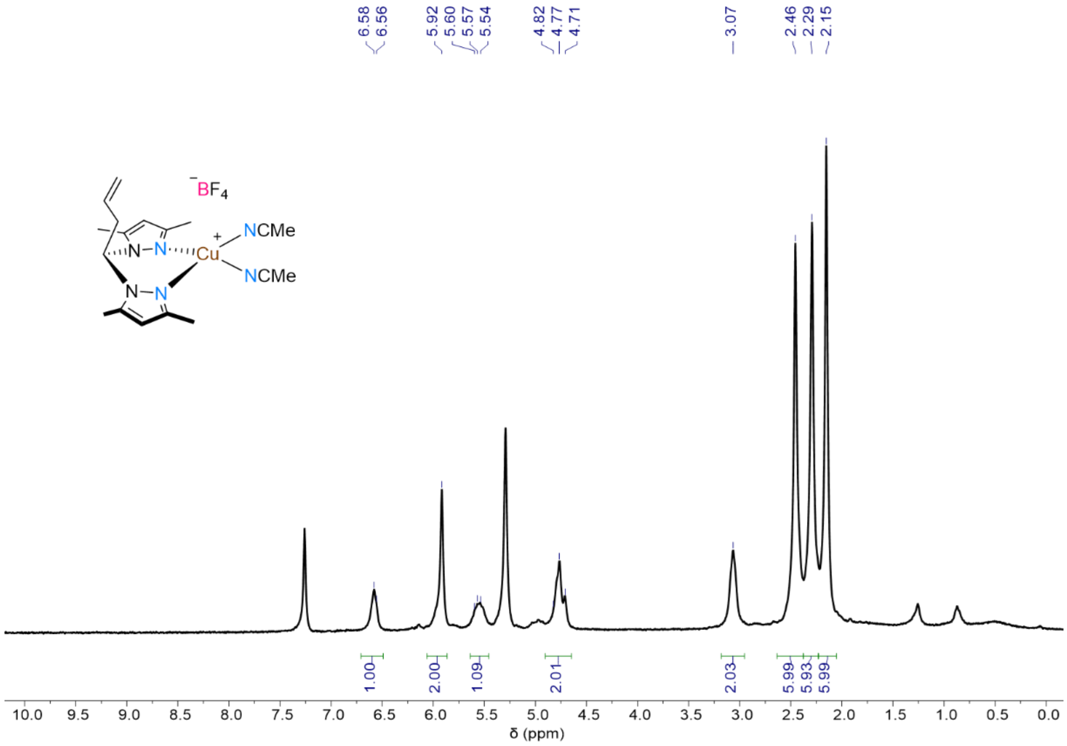

Figure S8 ^{1}H NMR spectrum (300 MHz, $CDCl_3$) of complex **2a**.

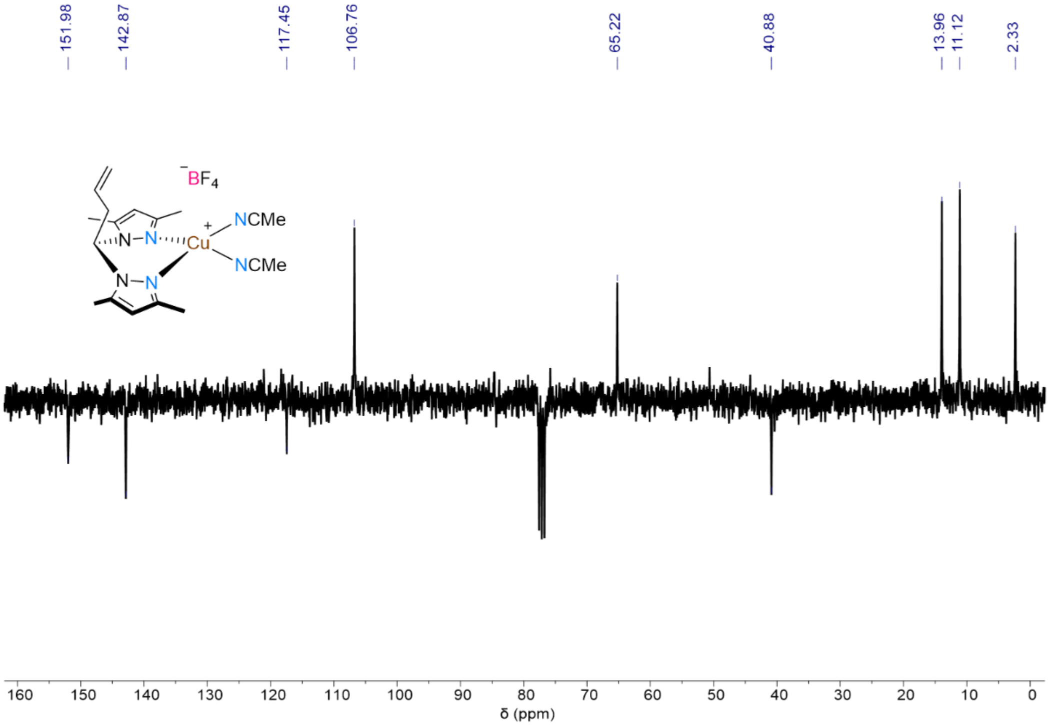

Figure S9 ^{13}C APT NMR spectrum (75 MHz, $CDCl_3$) of complex **2a**.

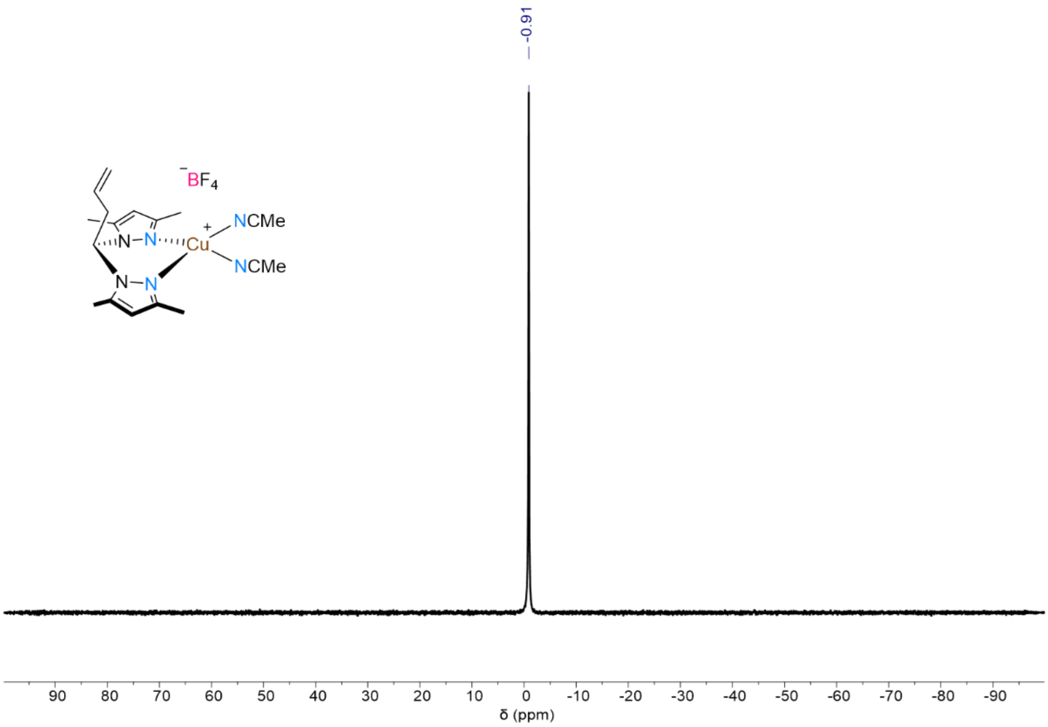

Figure S10 ^{11}B NMR spectrum (96 MHz, $CDCl_3$) of complex **2a**.

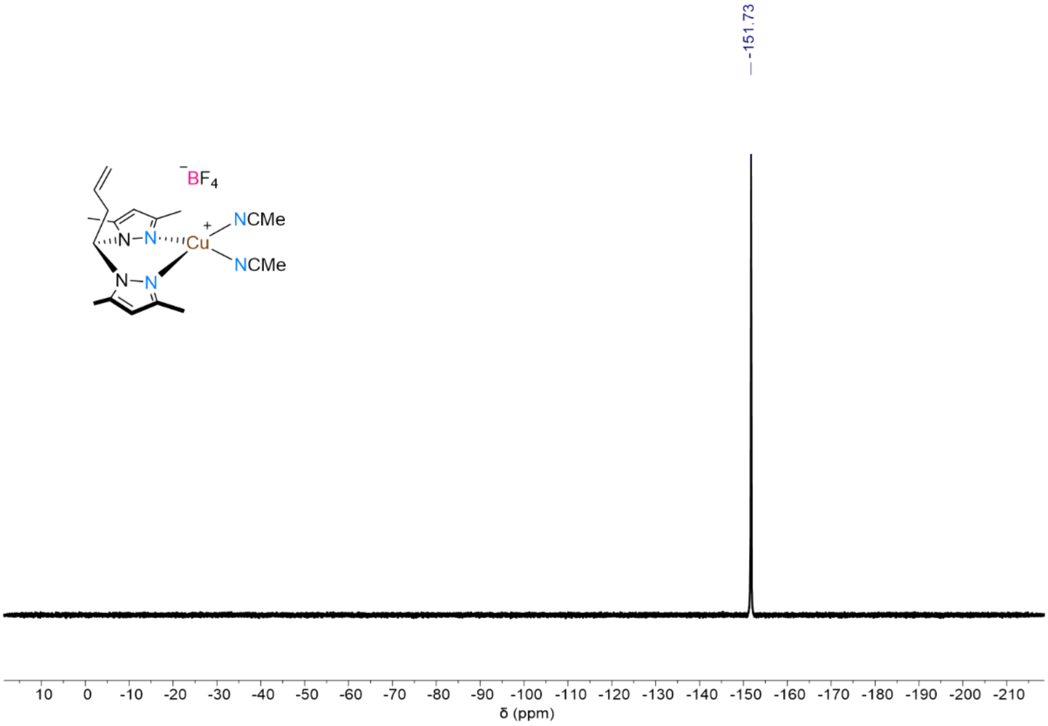

Figure S11 $^{19}F\{^{1}H\}$ NMR spectrum (282 MHz, $CDCl_3$) of complex **2a**.

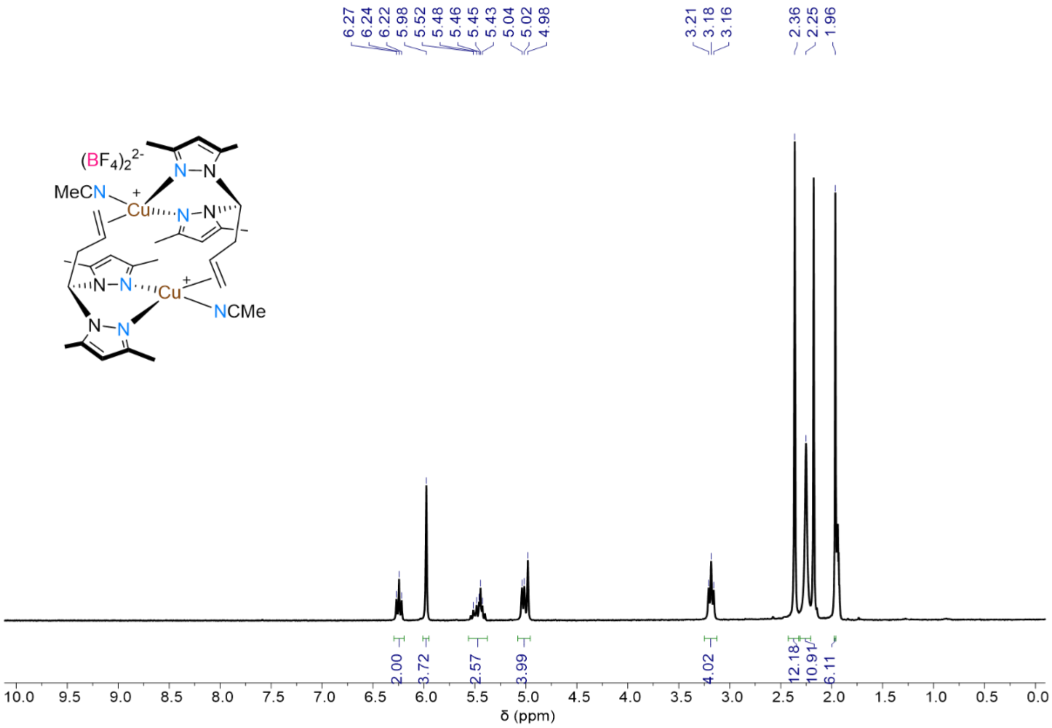

Figure S12 ^{1}H NMR spectrum (300 MHz, CD_3CN) of complex **2a$_2$**.

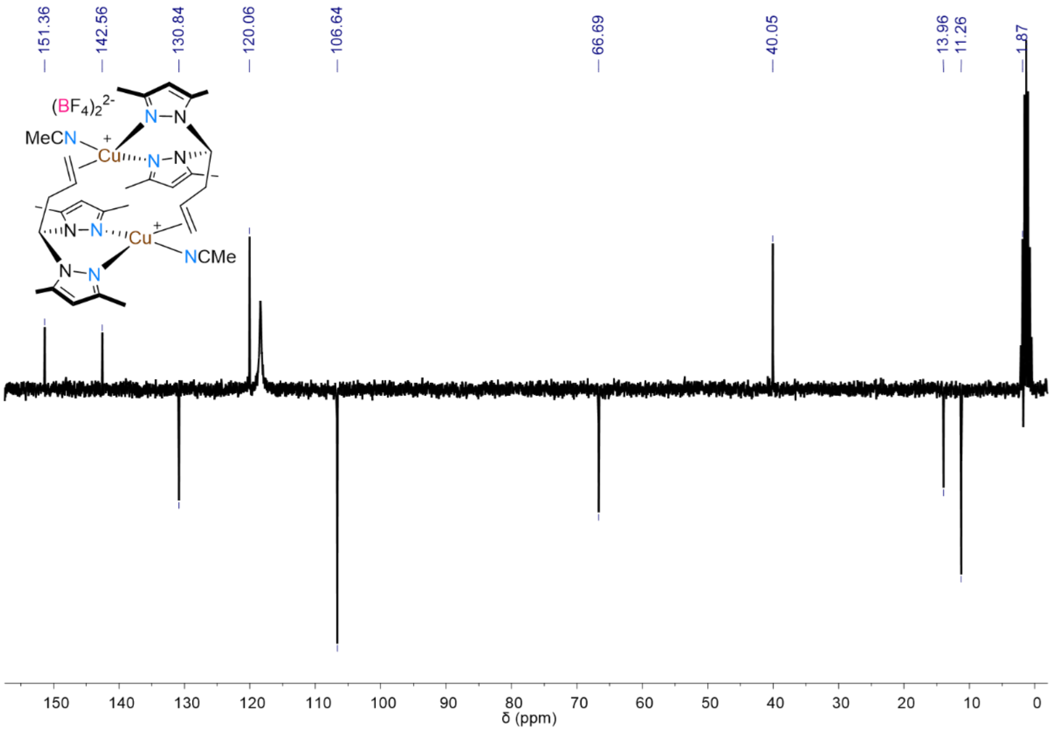

Figure S13 ^{13}C APT NMR spectrum (75 MHz, CD_3CN) of complex **2a$_2$**.

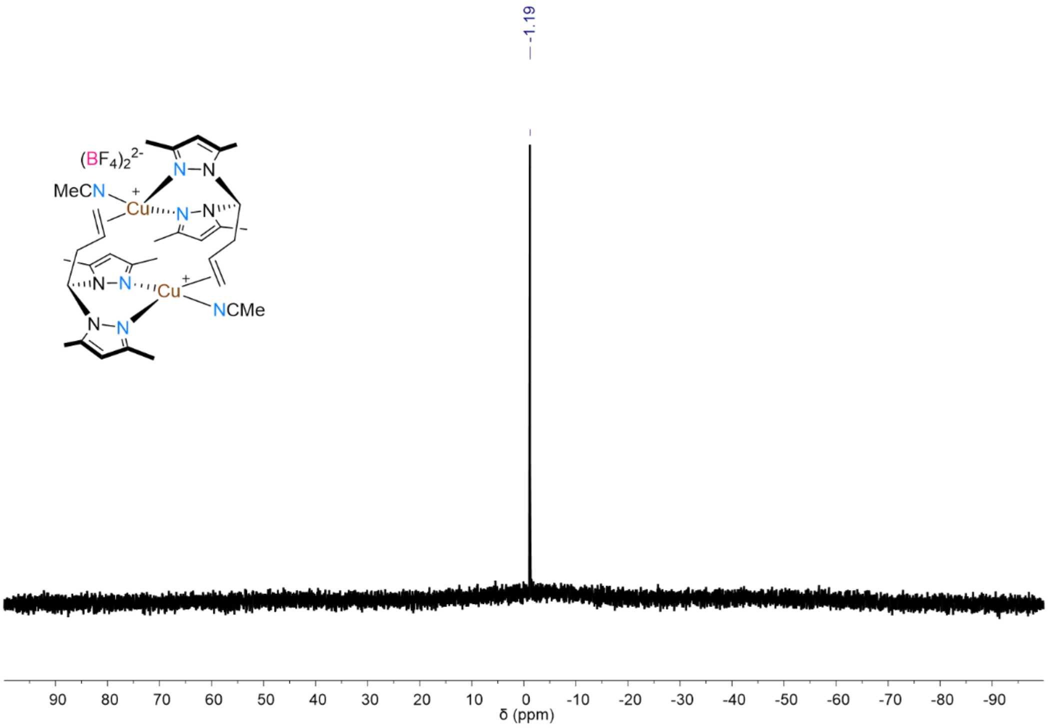

Figure S14 ^{11}B NMR spectrum (96 MHz, CD_3CN) of complex **2a$_2$**.

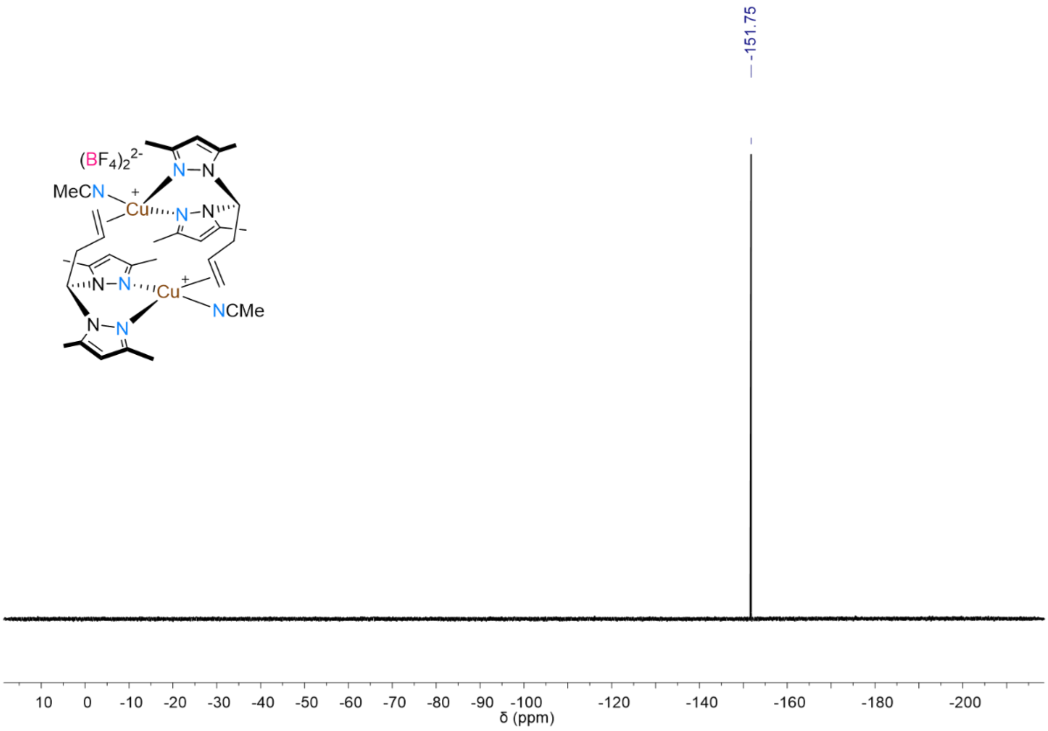

Figure S15 ^{19}F{^{1}H} NMR spectrum (282 MHz, CD_3CN) of complex **2a$_2$**.

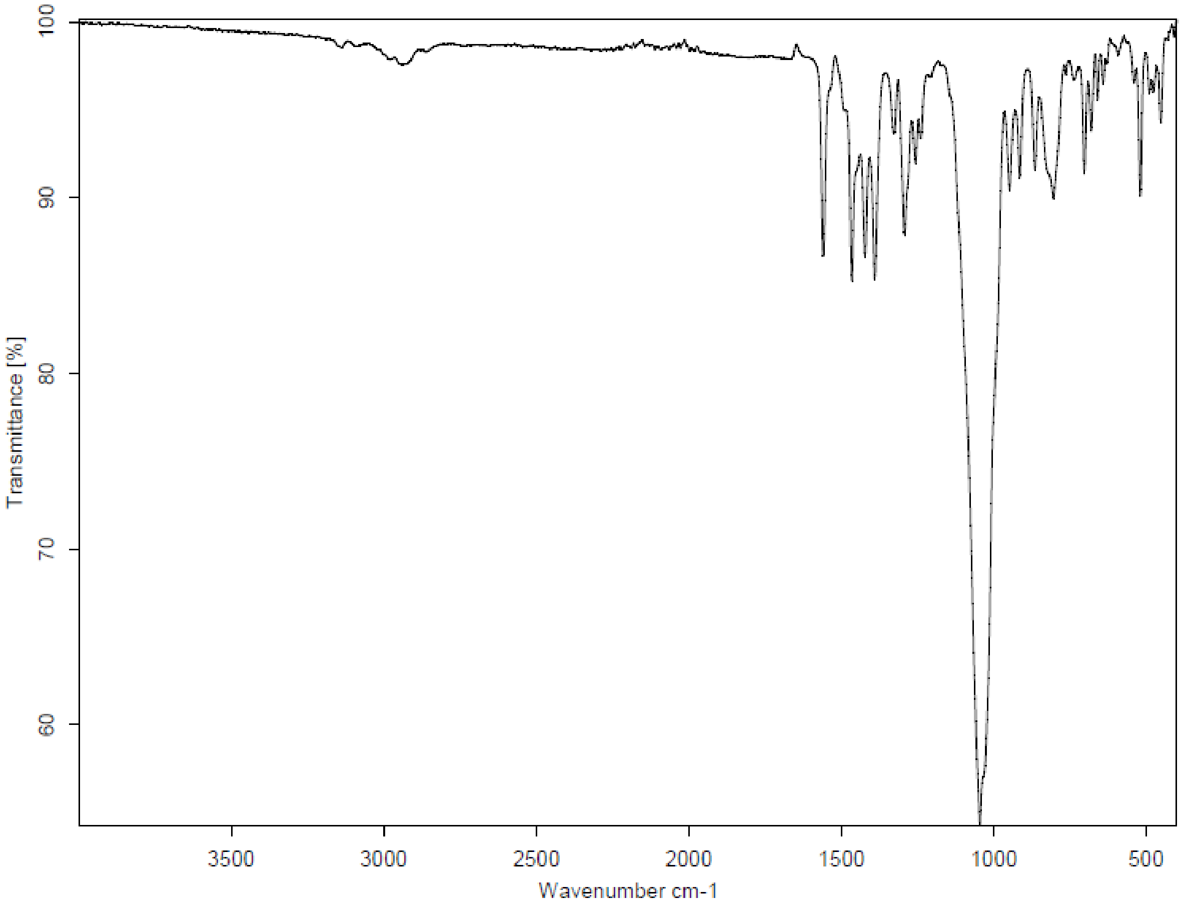

Figure S16 ATR-FTIR spectrum of complex **2a2**.

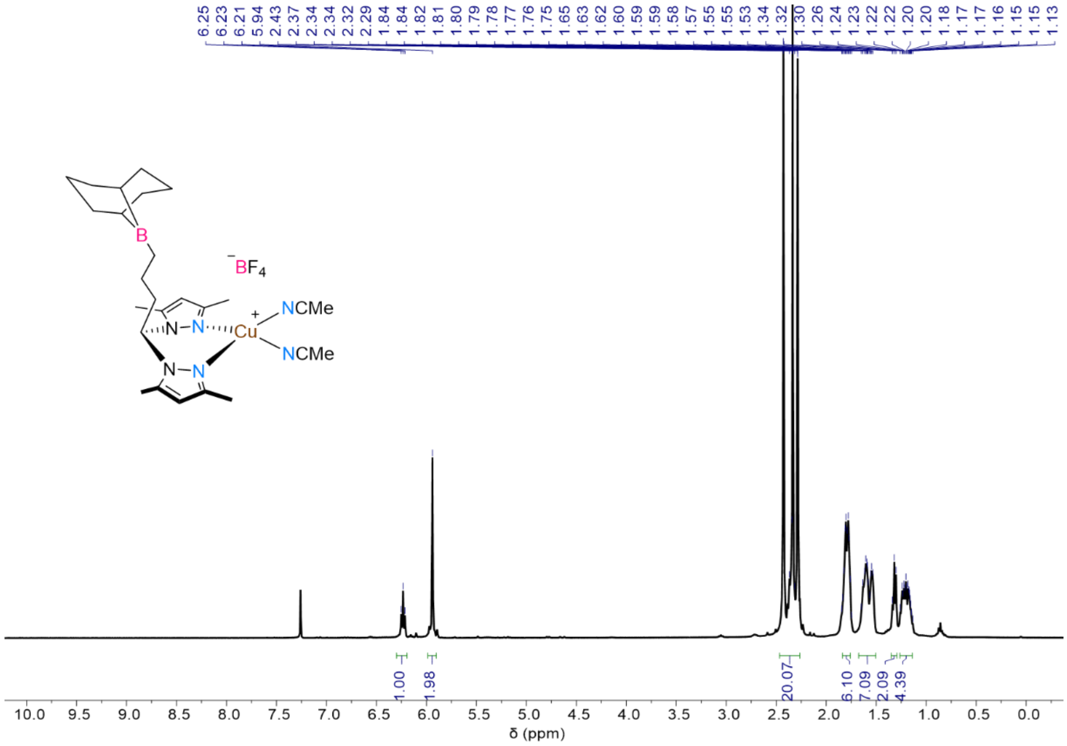

Figure S17 ^{1}H NMR spectrum (300 MHz, $CDCl_3$) of complex **2b**.

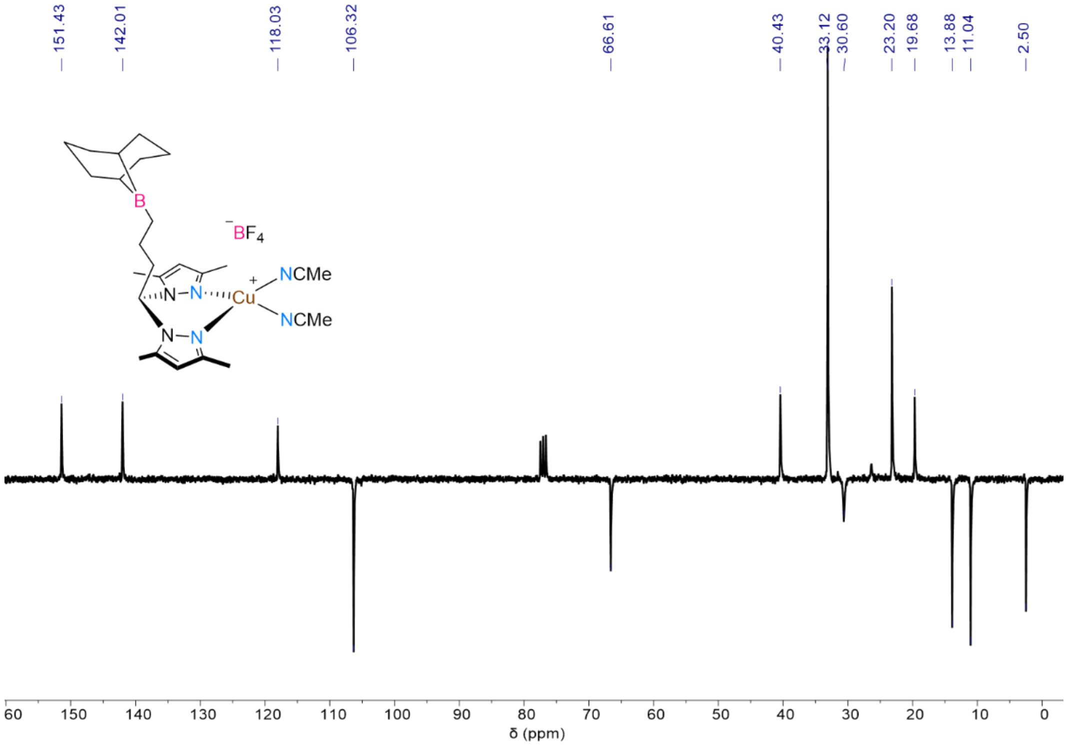

Figure S18 ^{13}C APT NMR spectrum (75 MHz, $CDCl_3$) of complex **2b**.

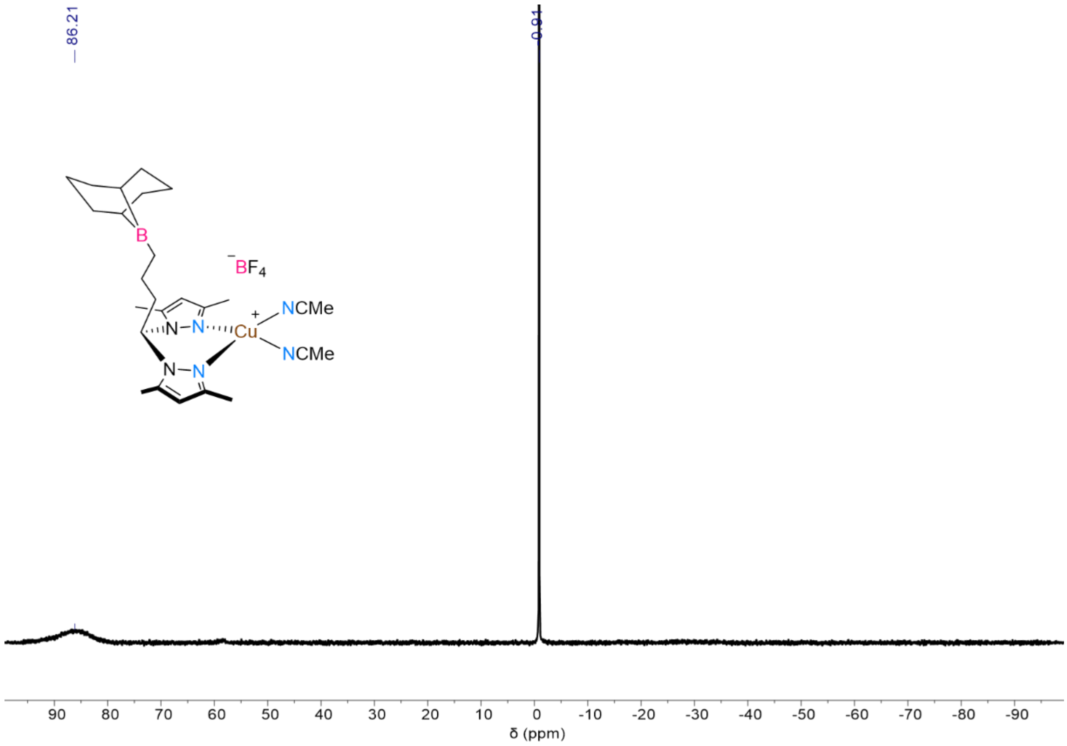

Figure S19 ^{11}B NMR spectrum (96 MHz, $CDCl_3$) of complex **2b**.

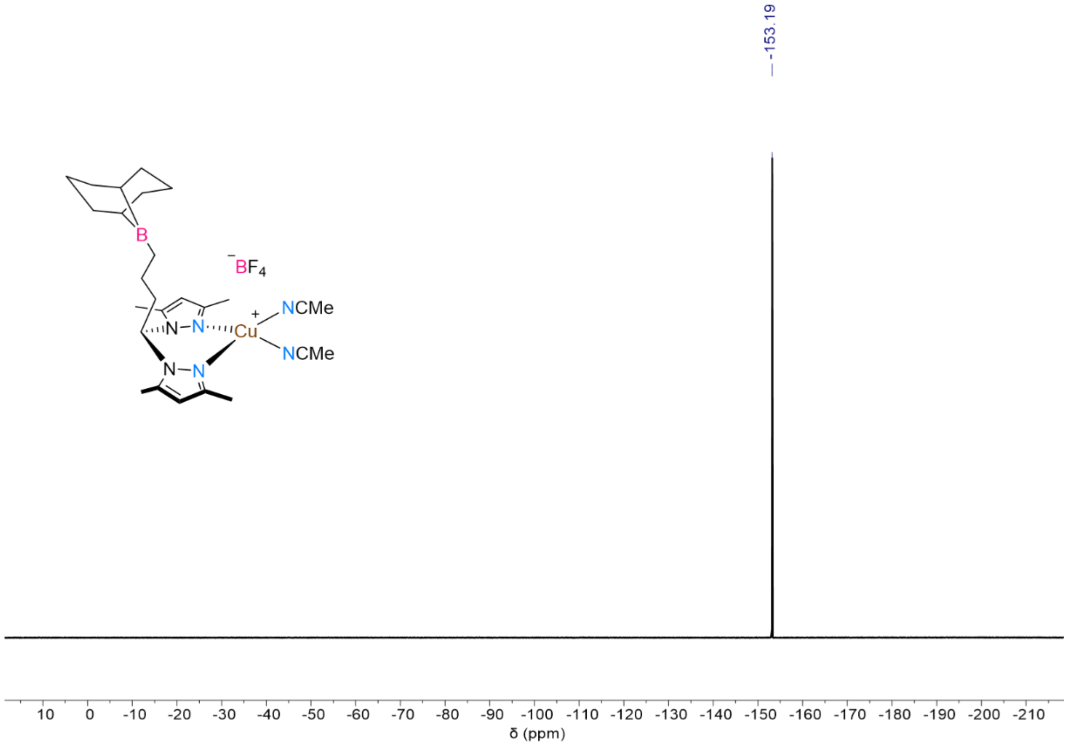

Figure S20 $^{19}F\{^1H\}$ NMR spectrum (282 MHz, $CDCl_3$) of complex **2b**.

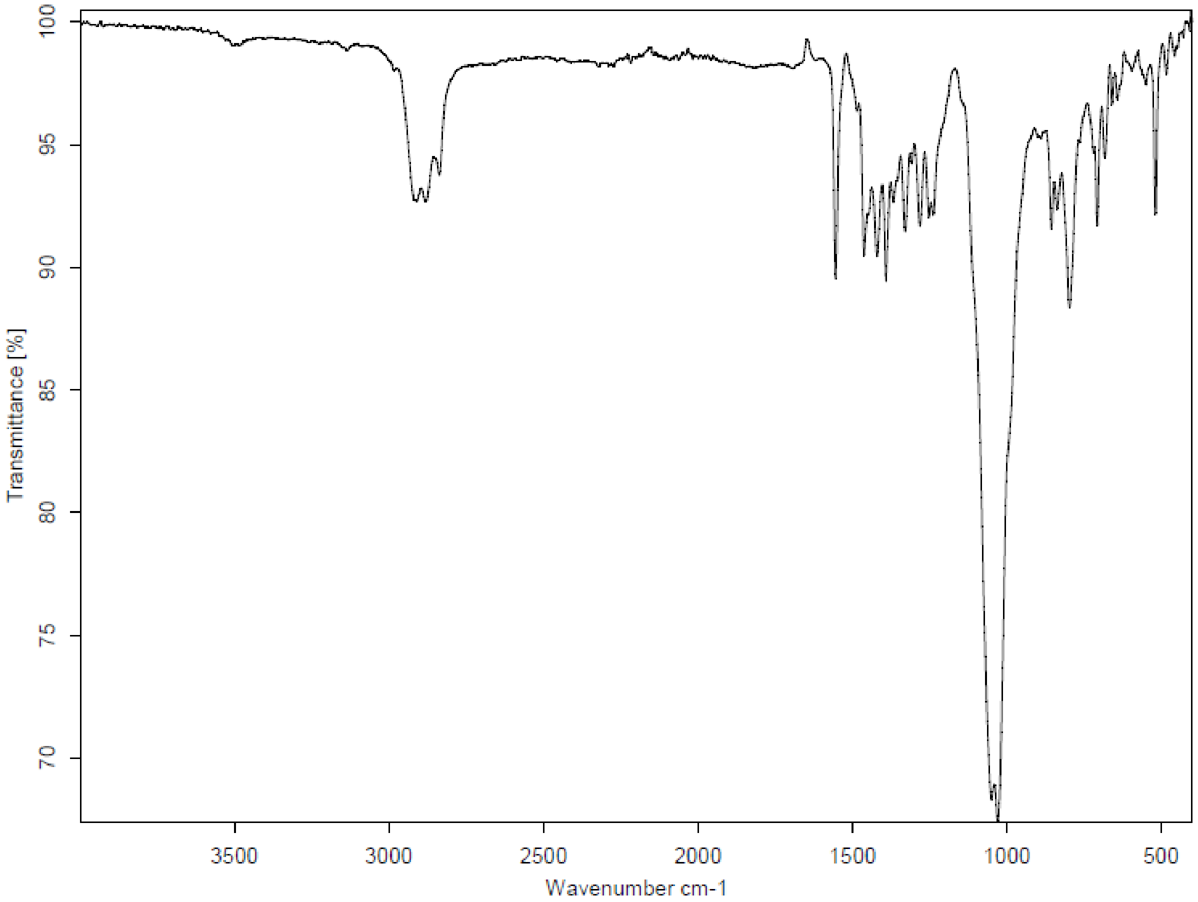

Figure S21 ATR-FTIR spectrum of complex **2b**.

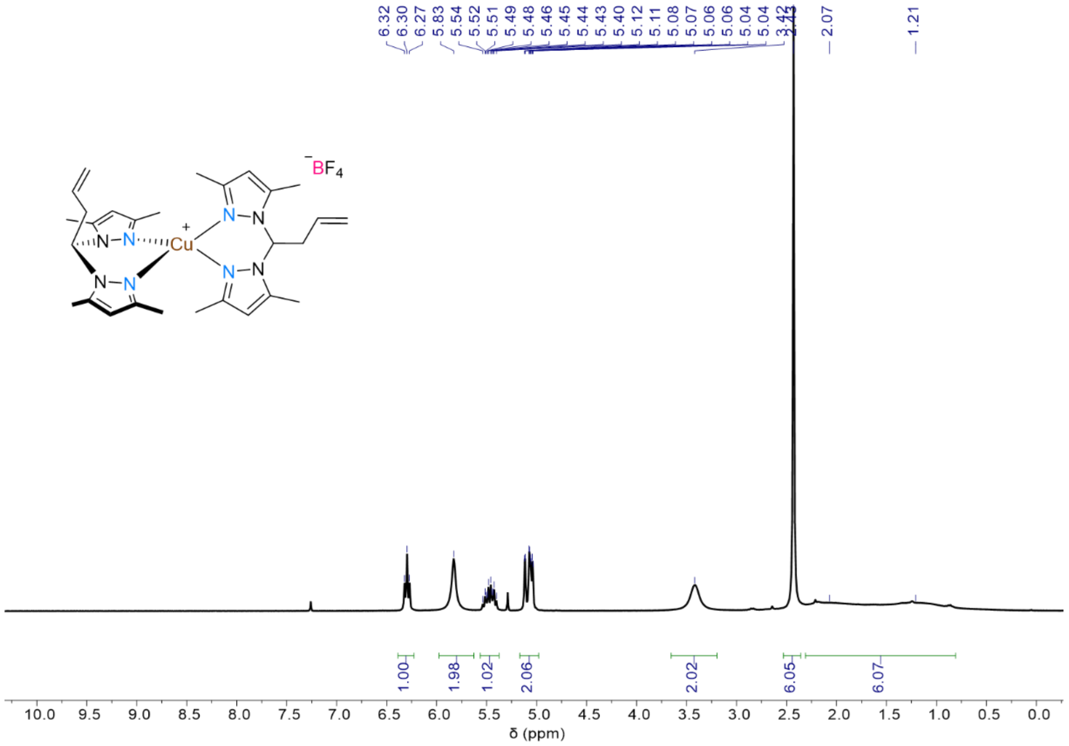

Figure S22 ^{1}H NMR spectrum (300 MHz, $CDCl_3$) of complex **3a**.

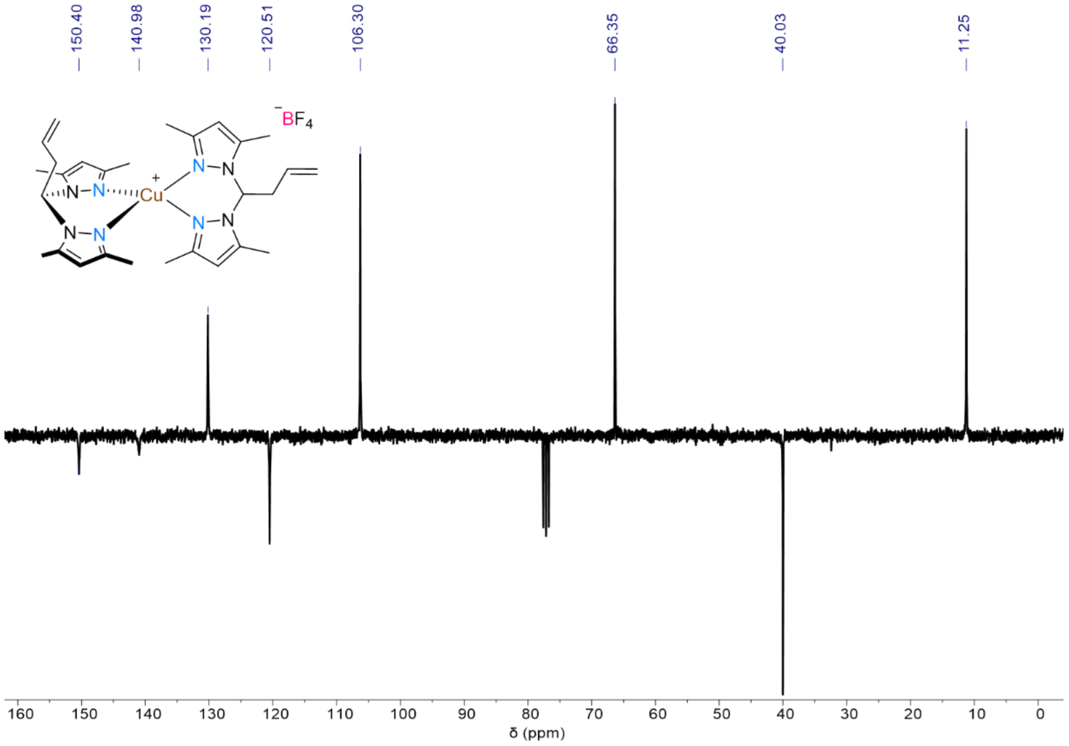

Figure S23 ^{13}C APT NMR spectrum (75 MHz, $CDCl_3$) of complex **3a**.

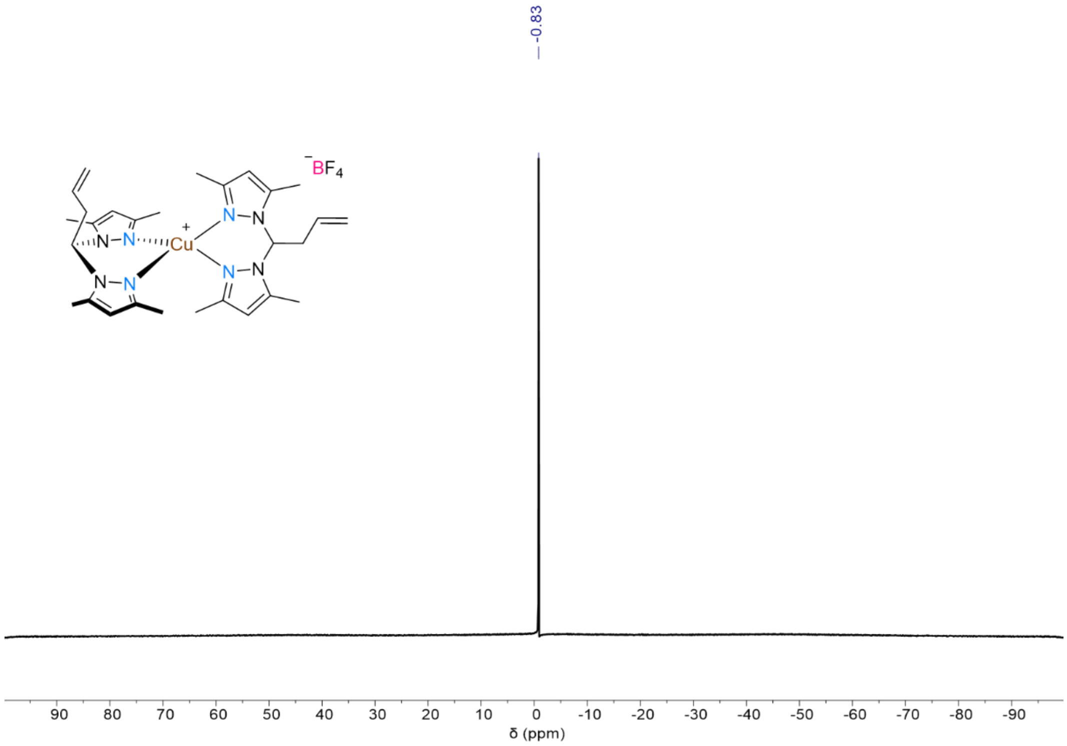

Figure S24 ^{11}B NMR spectrum (96 MHz, $CDCl_3$) of complex **3a**.

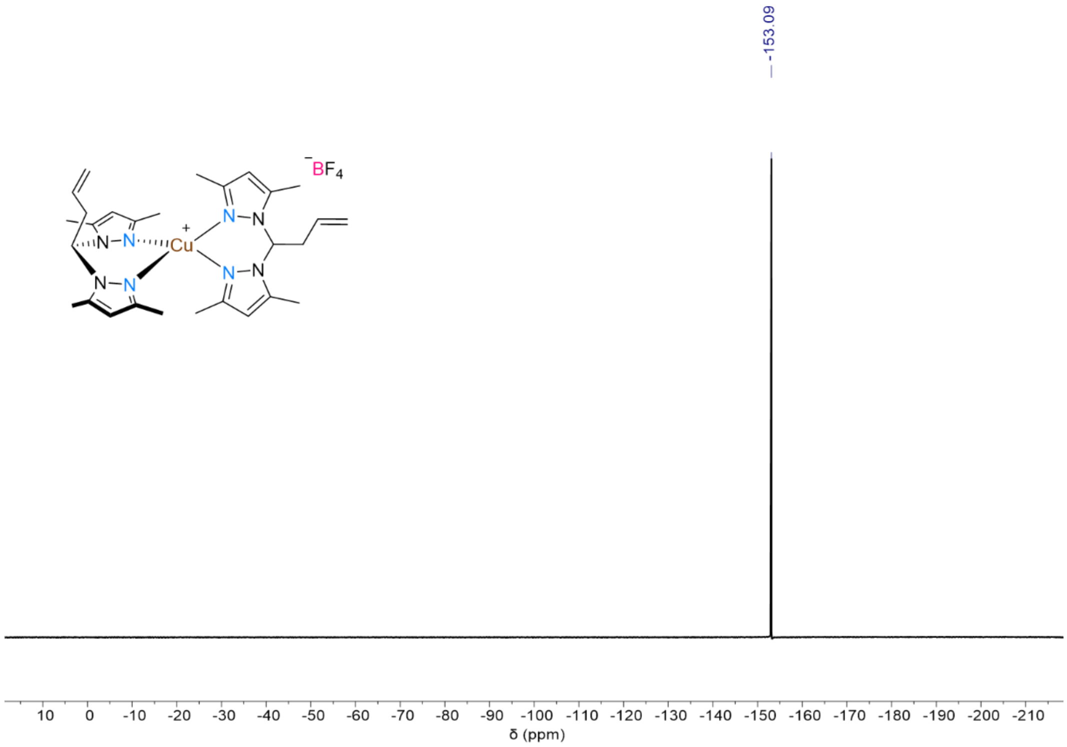

Figure S25 $^{19}F\{^1H\}$ NMR spectrum (282 MHz, $CDCl_3$) of complex **3a**.

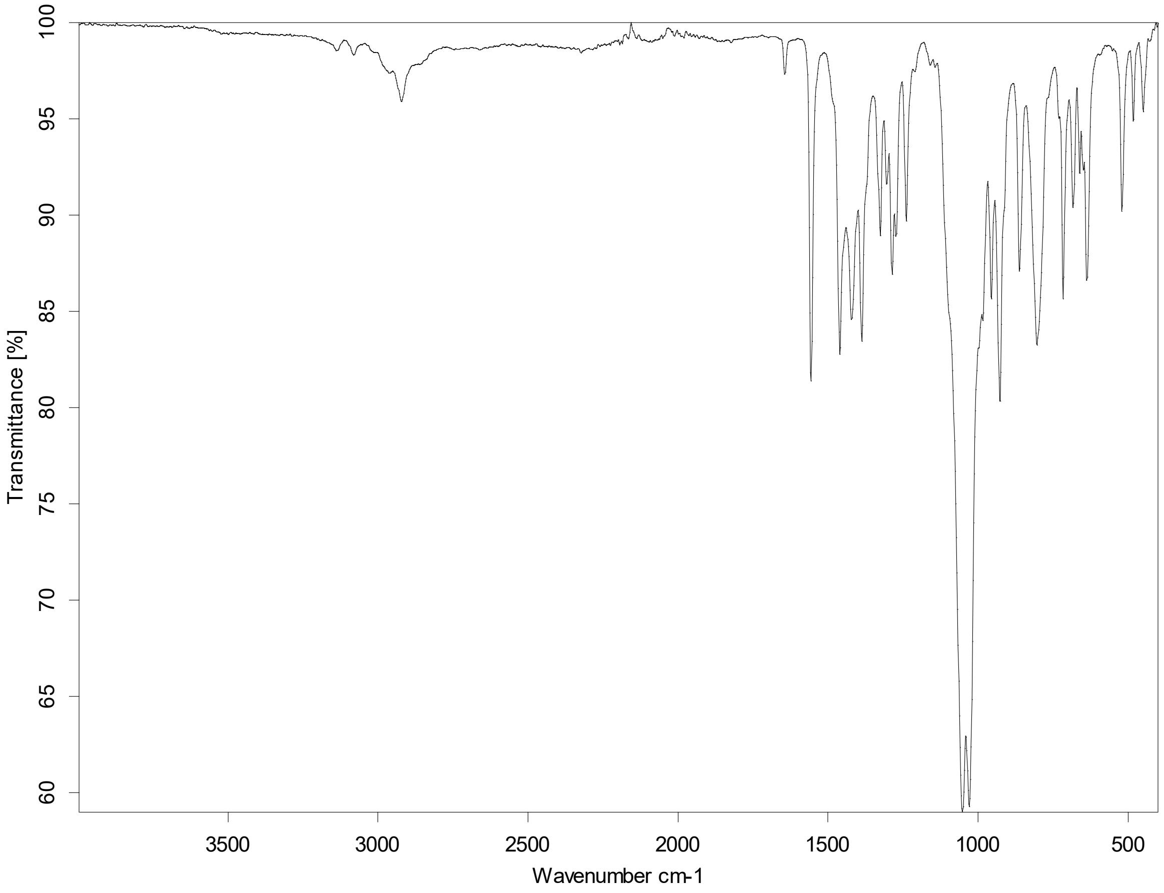

Figure S26 ATR-FTIR spectrum of complex **3a**.

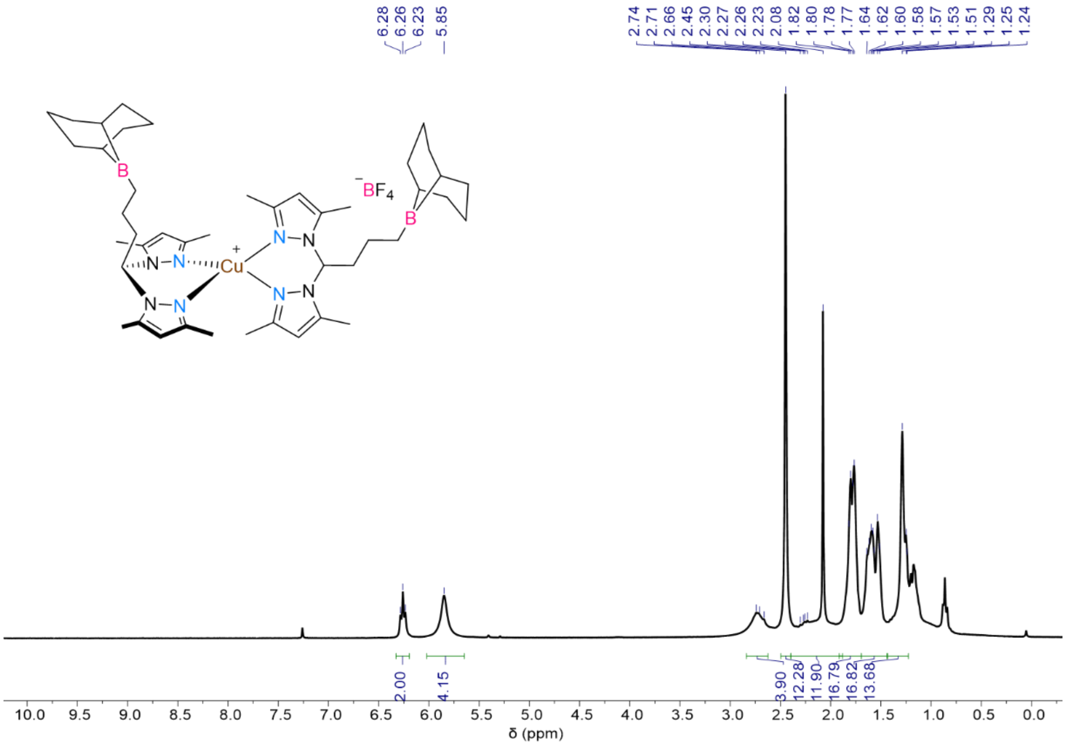

Figure S27 ^{1}H NMR spectrum (300 MHz, $CDCl_3$) of complex **3b**.

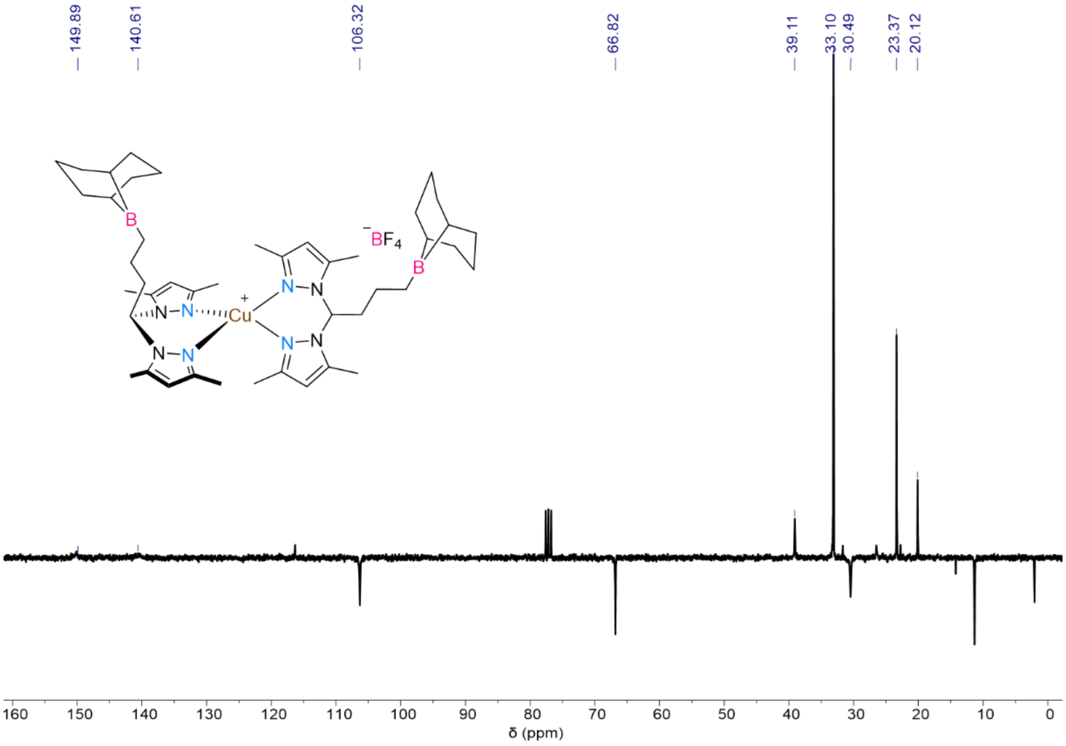

Figure S28 ^{13}C APT NMR spectrum (75 MHz, $CDCl_3$) of complex **3b**.

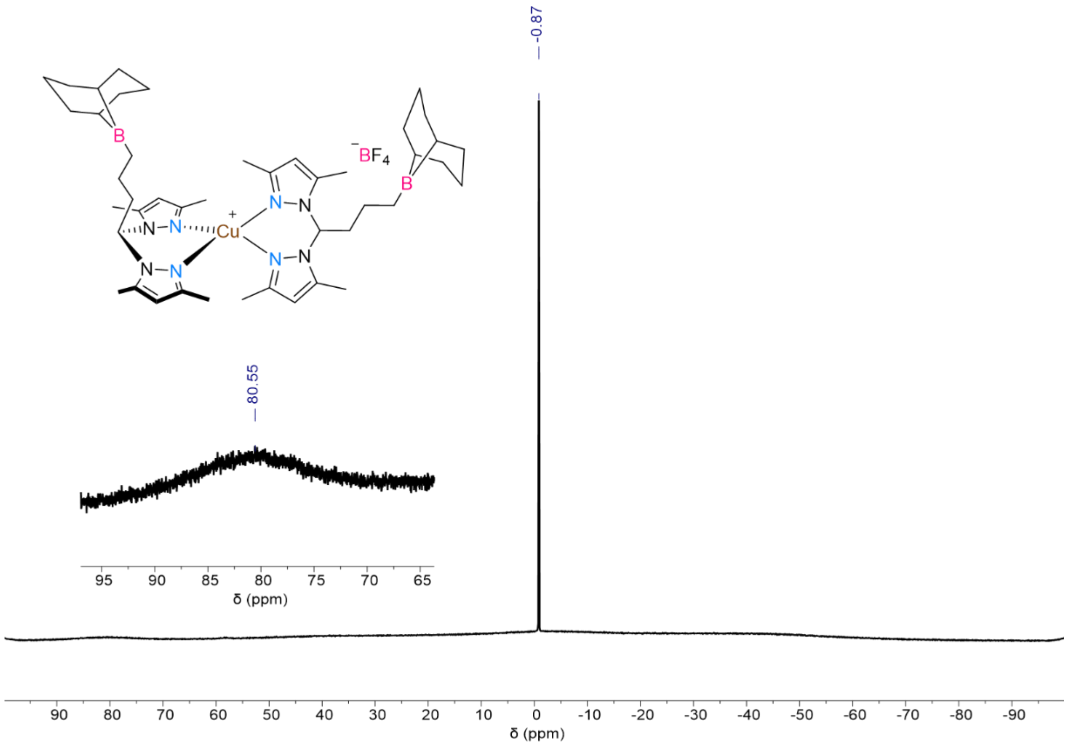

Figure S29 ^{11}B NMR spectrum (96 MHz, $CDCl_3$) of complex **3b**.

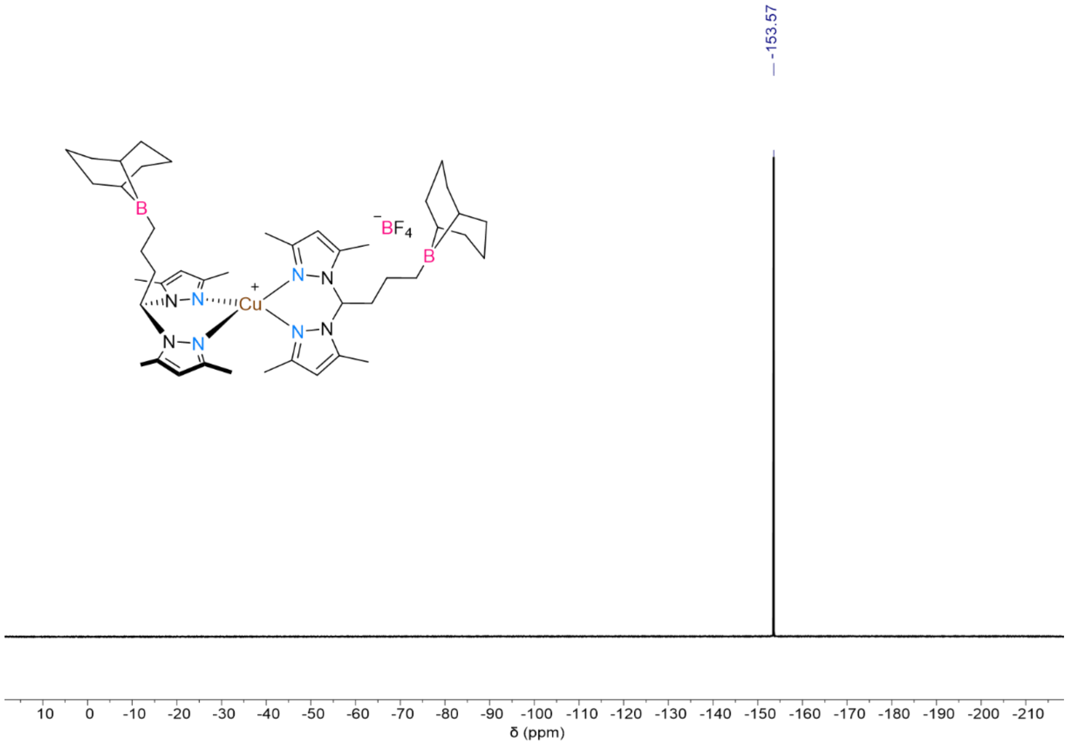

Figure S30 $^{19}F\{^1H\}$ NMR spectrum (282 MHz, $CDCl_3$) of complex **3b**.

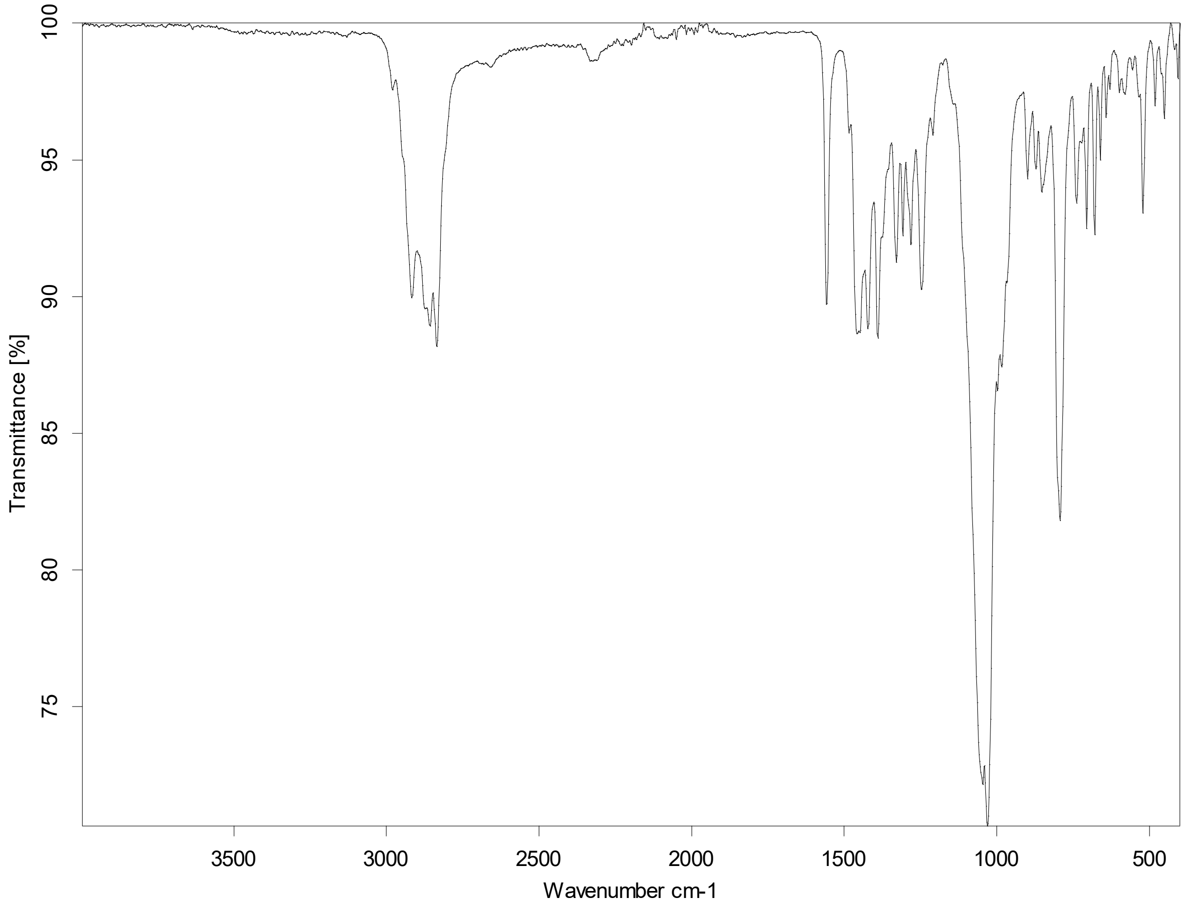

Figure S31 ATR-FTIR spectrum of complex **3b**.

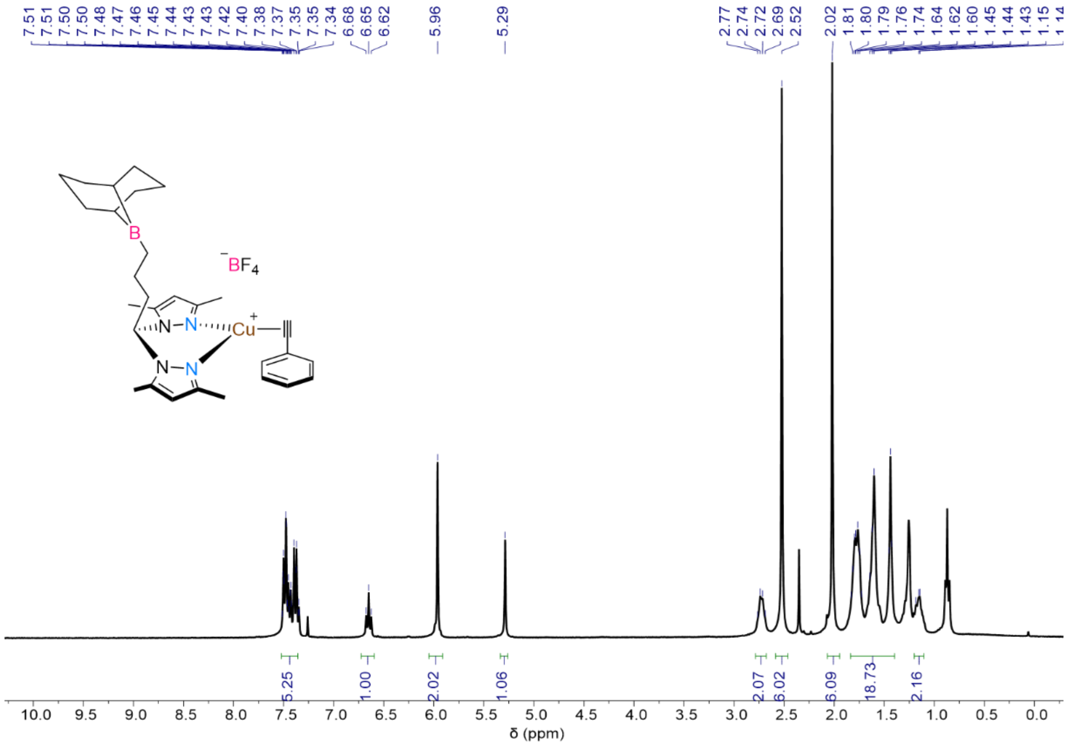

Figure S32 ^{1}H NMR spectrum (300 MHz, $CDCl_3$) of complex **4**.

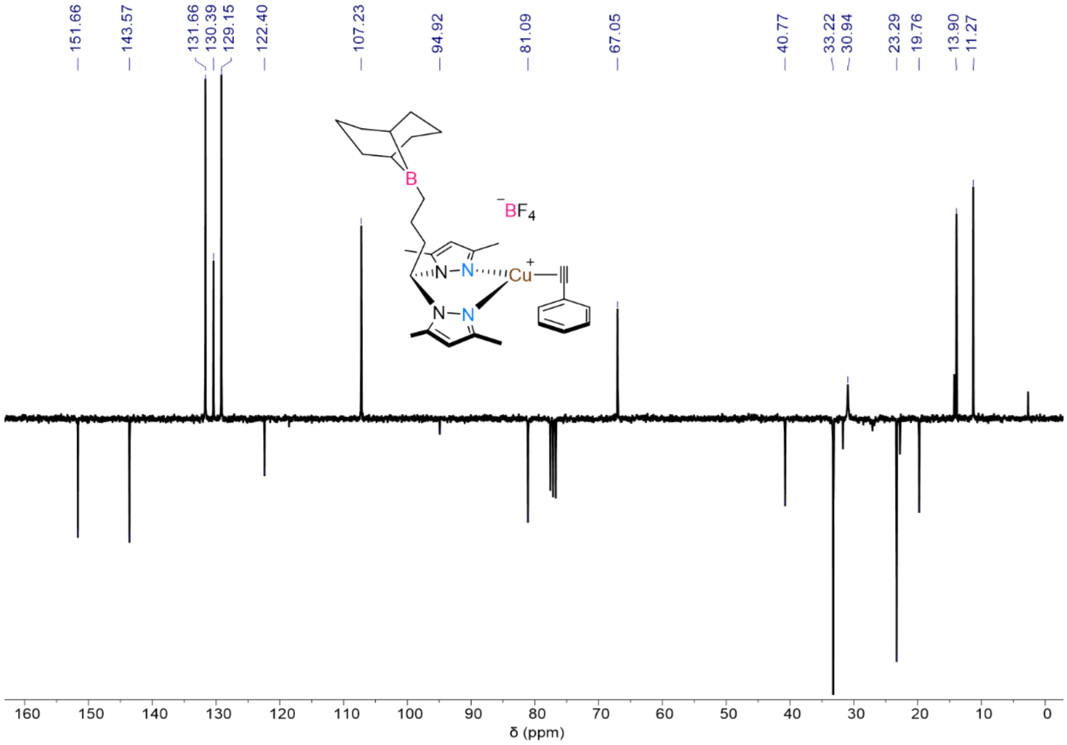

Figure S33 ^{13}C APT NMR spectrum (75 MHz, $CDCl_3$) of complex **4**.

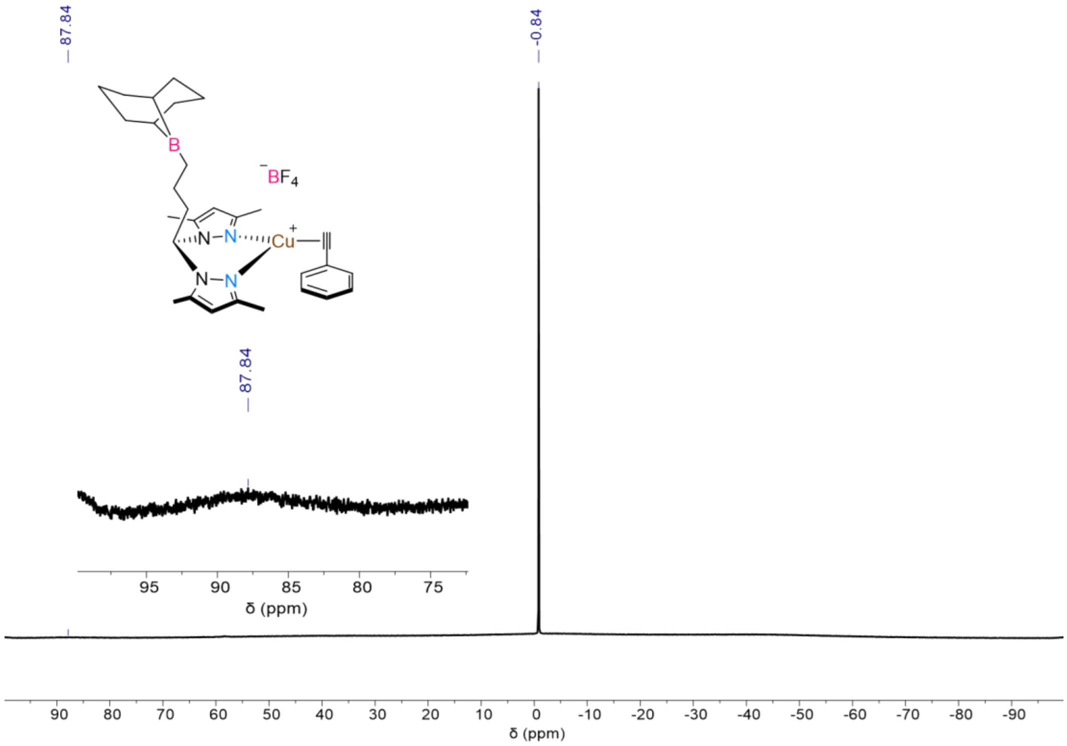

Figure S34 ^{11}B NMR spectrum (96 MHz, $CDCl_3$) of complex **4**. The inset corresponds to a magnification of the zone of the spectrum between 70 and 100 ppm.

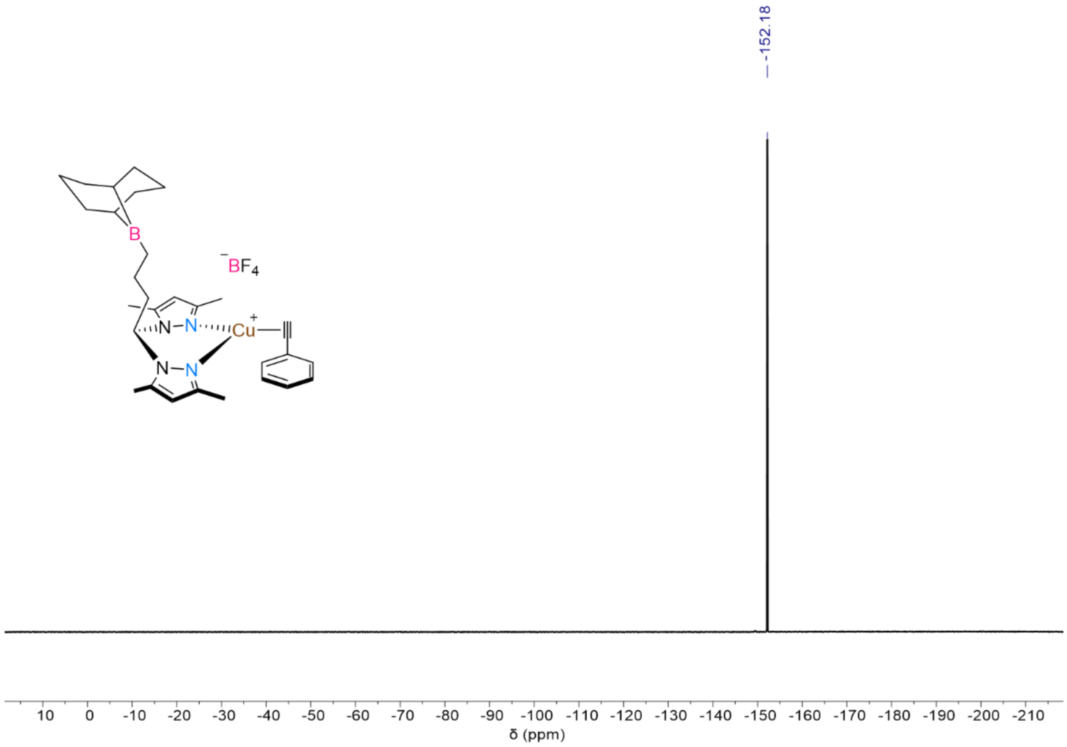

Figure S35 $^{19}F\{^{1}H\}$ NMR spectrum (282 MHz, $CDCl_3$) of complex **4**.

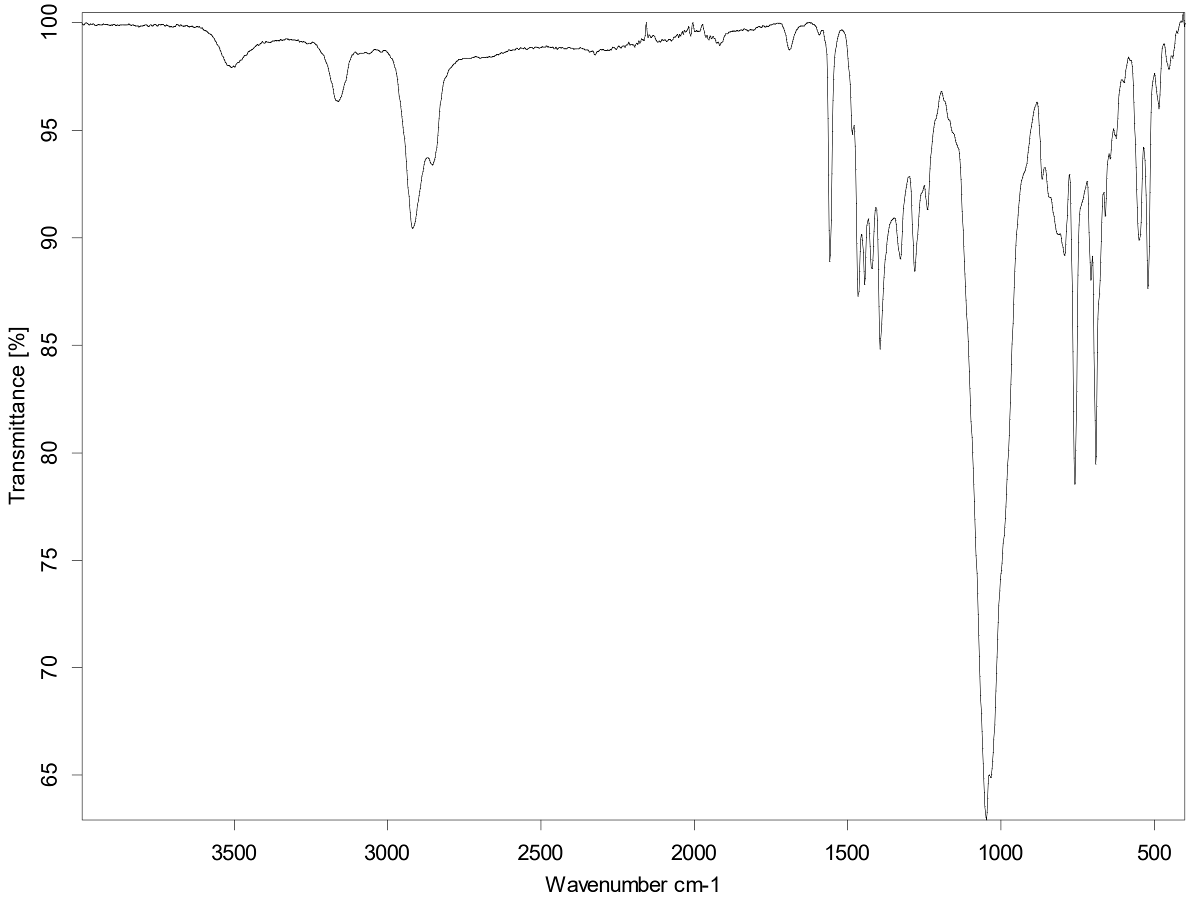

Figure S36 ATR-FTIR spectrum of complex **4**.

Supplementary single-crystal X-ray diffraction data

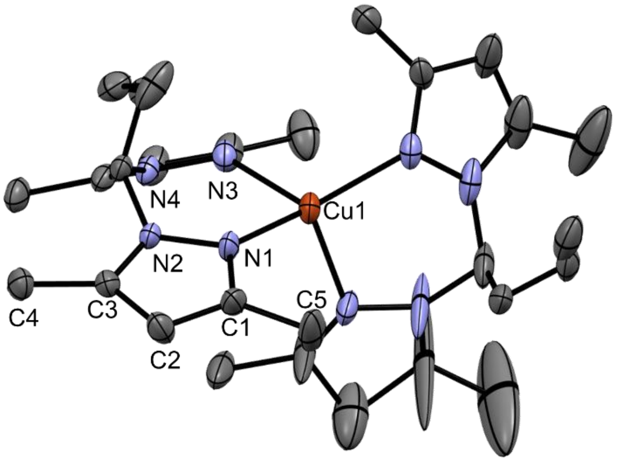

Figure S37 Proof of the molecular connectivity of the cation of complex **3a** determined by single-crystal X-ray diffraction. All hydrogen atoms and tetrafluoroborate anion were omitted for clarity.

Table S1 Selected bond distances (Å) and bond angles (º) for complexes **1a**, **$2a_2$** and **3a**.

**1a**		**$2a_2$**		**3a**	
Lengths (Å)		*Lengths (Å)*		*Lengths (Å)*	
Cu1–N3	2.010(4)	Cu1–N1	2.021(3)	Cu1–N3	1.995(6)
Cu1–N1	2.032(4)	Cu1–N3	2.047(3)	Cu1–N1	2.162(6)
Cu1–Cl1	2.1577(15)	Cu1–N5	2.116(4)	N3–C6	1.328(10)
N4–N3	1.375(5)	Cu1–C14	2.118(4)	N3–N4	1.367(7)
N1–C1	1.333(6)	Cu1–C13	2.169(4)	N2–C3	1.353(8)
N1–N2	1.378(5)	N1–C1	1.331(5)	N2–N1	1.375(6)
N2–C3	1.352(6)	N1–N2	1.372(4)	N1–C1	1.323(9)
C1–C2	1.411(7)	N2–C3	1.358(5)	C1–C2	1.378(9)
C1–C5	1.519(6)	C3–C2	1.364(6)	C1–C5	1.511(9)
C2–C3	1.387(7)	C3–C4	1.500(6)	C2–C3	1.380(9)
C4–C3	1.492(6)	C1–C2	1.393(6)	C3–C4	1.492(9)
		C1–C5	1.492(7)		
Angles (º)				*Angles (º)*	
N3–Cu1–N1	93.06(16)	*Angles (º)*		N3–Cu1–N7	146.1(2)
N3–Cu1–Cl1	137.02(12)	N1–Cu1–N3	91.47(13)	N3–Cu1–N5	108.2(2)
N1–Cu1–Cl1	129.91(12)	N1–Cu1–N5	111.60(14)	N7–Cu1–N5	94.2(3)
C1–N1–N2	104.3(4)	N3–Cu1–N5	106.90(17)	N3–Cu1–N1	93.6(2)
N1–C1–C2	111.7(4)	N1–Cu1–C14	107.70(15)	N7–Cu1–N1	106.2(3)
N1–C1–C5	119.5(5)	N3–Cu1–C14	134.46(14)	N5–Cu1–N1	102.6(3)
C2–C1–C5	128.8(5)	N5–Cu1–C14	103.65(18)	C3–N2–N1	111.8(5)
C3–C2–C1	105.2(5)	N1–Cu1–C13	142.38(14)	C1–N1–N2	104.3(5)
N4–C11–N2	110.9(4)	N3–Cu1–C13	109.46(14)	N1–C1–C2	112.1(6)
N2–C3–C2	106.5(4)	N5–Cu1–C13	92.31(15)	N1–C1–C5	120.3(6)
N2–C3–C4	124.4(4)	C14–Cu1–C13	36.07(16)	C2–C1–C5	127.6(7)
C2–C3–C4	129.1(5)	C1–N1–N2	105.5(3)	C3–C2–C1	106.0(6)
		C3–N2–N1	111.2(3)	C2–C3–N2	105.9(5)
		N4–C11–N2	109.9(3)	C2–C3–C4	130.4(6)
		N2–C3–C2	106.1(4)	N2–C3–C4	123.7(6)
		N2–C3–C4	122.3(4)		
		C2–C3–C4	131.6(4)		
		N1–C1–C2	110.1(4)		
		N1–C1–C5	121.3(4)		
		C2–C1–C5	128.5(4)		
		C3–C2–C1	107.1(4)		

Table S2 Crystallographic data for complexes **1a**, **2a$_2$** and **3a**.

	**1a**	**2a$_2$**	**3a**
Formula	$C_{14}H_{20}ClCuN_4$	$C_{16}H_{23}BCuF_4N_5$	$C_{28}H_{40}BCuF_4N_8$
M	343.33	435.74	639.03
λ (Å)	0.71073	0.71073	0.71073
T (K)	150	150	150
Crystal system	Monoclinic	Monoclinic	Triclinic
Space group	Cc	P 2_1/n	P -1
a (Å)	9.393(3)	13.002(4)	8.226(11)
b (Å)	25.453(9)	8.038(2)	13.824(17)
c (Å)	7.699(2)	18.973(7)	14.502(18)
α (Å)	90	90	100.60(3)
β (Å)	119.745(8)	92.813(10)	96.12(4)
γ (Å)	90	90	97.88(3)
V (Å^3)	1598.2	1980.4(11)	1591(3)
Z	4	4	2
ρ_{calc} (g.cm^{-3})	1.427	1.461	1.334
μ (mm^{-1})	1.529	1.149	0.741
Crystal size (mm)	0.40×0.10×0.04	0.30×0.15×0.15	0.02×0.02×0.10
θ_{max} (º)	26.444	27.534	26.768
Total data	4537	48292	12173
Unique data	2764	4287	6633
R_{int}	0.0340	0.2049	0.0482
R [$I > 2\sigma(I)$]	0.0352	0.0579	0.1131
R_w	0.0512	0.1007	0.1566
Goodness of fit	0.889	0.955	1.272
ρ_{min}	-0.304	-0.615	-0.924
ρ_{max}	0.278	0.877	1.897

Supplementary cyclic voltammetry data

Table S3 Electrochemical data for all complexes.

Complex	E_p^{an}	E_p^{cat}	$E_{1/2}^{an}$	I_p^{an}/I_p^{cat}
**1a**	0.53	0.36	0.44	-[a]
**1b**	0.78	0.19	0.28	-[a]
**2a₂**	0.78	0.36	0.57	1.3
**2b**	0.60	-0.01	0.30	4
**3a**	0.36	0.24	0.30	1
**4**	0.92	-	-	-[b]

Anodic and corresponding cathodic counterparts potentials and current intensity ratio for the oxidation of the various complexes. Potentials measured versus ferrocene/ferrocenium couple in $N(n\text{-}Bu)_4]BF_4/CH_2Cl_2$ at 200 mV s^{-1}. [a] Cathodic counterpart intensities not measured. [b] Irreversible.

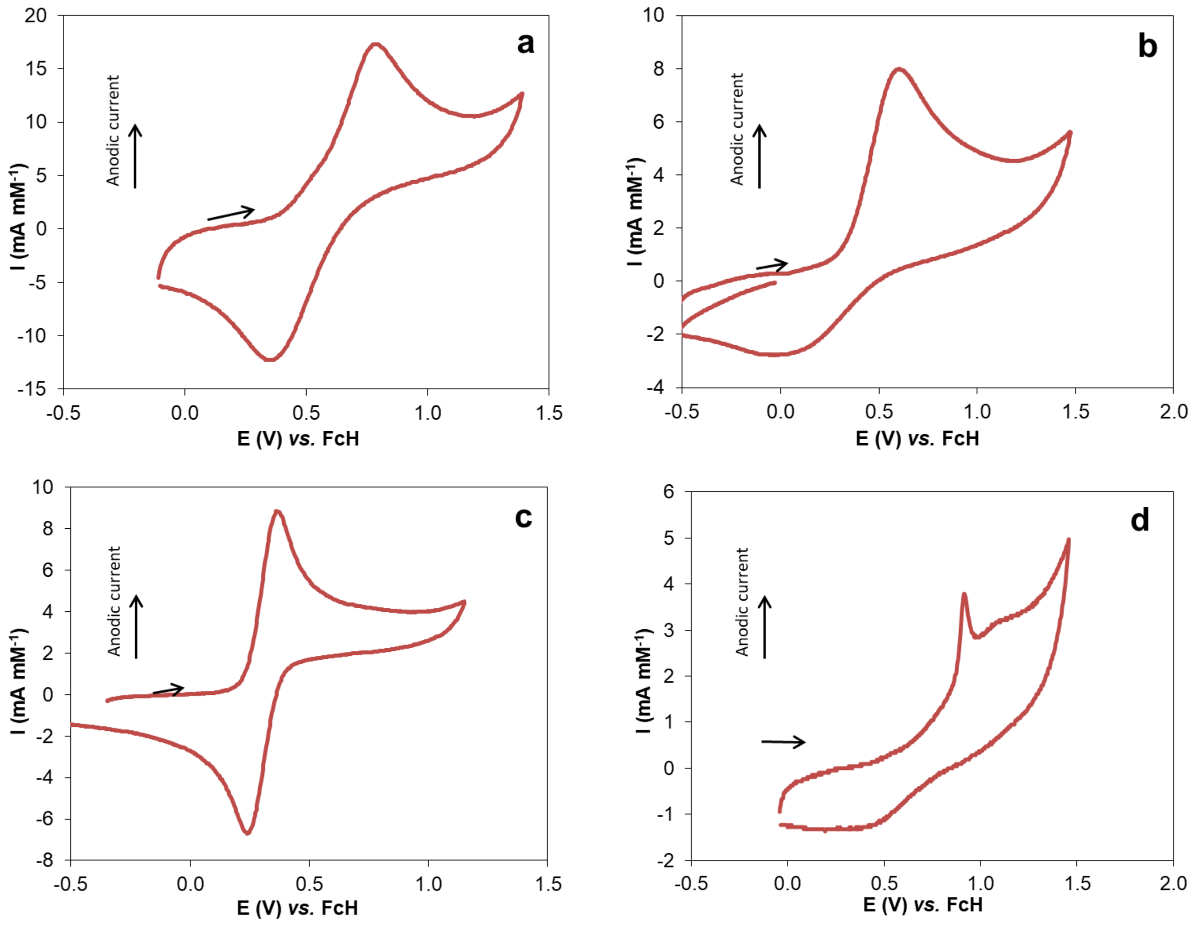

Figure S38 Cyclic voltammograms of complexes **2a₂** (a), **2b** (b), **3a** (c) and **4** (d) in $[N(n\text{-}Bu)_4]BF_4/CH_2Cl_2$ at 200 mV s^{-1}.

Results of the catalytic cycloaddition reactions

Table S4 Cycloaddition of benzyl azide and phenylacetylene catalyzed by complex **1a**.

Cat. (mol%)	T (ºC)	Time (h)	Eq. DIPEA	TON	TOF (h^{-1})	Conversion (%)[a]
1	40	2.5	20	99	40	99
1	RT	2.5	20	98	39	98
0.33	40	1	20	21	21	7
0.33	RT	1	20	9	9	3
0.33	RT	2.5	20	33	13	11
0.33	40	1	40	164	137	54
0.33	40	2.5	40	264	106	87

$[\text{phenylacetylene}]_{\text{solvent}}$ = 1.24 M (0.495 mmol in 0.4 mL of CH_2Cl_2), ratio azide/alkyne = 1:1.5, benzyl azide was formed *in situ* by reaction of phenyl bromide and NaN_3 in a 1:1 ratio.

[a] Determined by ^1H NMR spectroscopy.

Table S5 Cycloaddition of benzyl azide and phenylacetylene catalyzed by the complex **1b**.

Cat. (mol%)	T (ºC)	Time (h)	Eq. DIPEA	TON	TOF (h^{-1})	Conversion (%)[a]
0.33	40	1	40	270	270	89
0.33	40	2.5	40	297	119	98
0.33	RT	1	40	42	42	14
0.33	RT	2.5	40	194	78	64

$[\text{phenylacetylene}]_{\text{solvent}}$ = 1.24 M (0.495 mmol in 0.4 mL of CH_2Cl_2), ratio azide/alkyne = 1:1.5, benzyl azide was formed *in situ* by reaction of phenyl bromide and NaN_3 in a 1:1 ratio.

[a] Determined by ^1H NMR spectroscopy.

Table S6 Cycloaddition of benzyl azide and phenylacetylene catalyzed by complex **2a$_2$**.

Cat. (mol% per Cu center)	Azide/alkyne ratio	T (ºC)	Time (h)	Eq. DIPEA	TON	TOF (h^{-1})	Conversion (%)[a]
1	1:1	40	2.5	-	10	4	10
1	1:1.5	40	2.5	-	55	22	55
1	1:1.5	40	2.5	20	98	39	98
1	1:1.5	RT	2.5	20	9	4	9
1	1:1.5	RT	2.5	40	94	38	94
0.33	1:1.5	40	2.5	20	273	109	90
0.33	1:1.5	40	1	40	46	46	15
0.33	1:1.5	40	2.5	40	294	118	97
0.33	1:1.5	RT	2.5	40	276	110	91

[phenylacetylene]$_{solvent}$ = 0.83 M (0.33 mmol in 0.4 mL of CH_2Cl_2), 1.03 M (0.4125 mmol in 0.4 mL of CH_2Cl_2), 1.24 M (0.495 mmol in 0.4 mL of CH_2Cl_2), benzyl azide was formed *in situ* by reaction of phenyl bromide and NaN_3 in a 1:1 ratio.

[a] Determined by ^{1}H NMR spectroscopy.

Table S7 Cycloaddition of benzyl azide and phenylacetylene catalyzed by complex **2b**.

Cat. (mol%)	Atmosphere	T (ºC)	Time (h)	Eq. DIPEA	TON	TOF (h^{-1})	Conversion (%)[a]
1	N_2	40	2.5	20	98	39	98
1	N_2	RT	2.5	20	91	36.4	91
0.33	N_2	40	1	20	109	109	36
0.33	N_2	RT	1	20	15	15	5
0.33	N_2	RT	2.5	20	30	12	10
0.33	N_2	40	1	40	294	294	97
0.33	N_2	40	2.5	40	297	119	98
0.33	Air	40	1	40	276	276	91

[phenylacetylene]$_{solvent}$ = 1.24 M (0.495 mmol in 0.4 mL of CH_2Cl_2, ratio azide/alkyne = 1:1.5, benzyl azide was formed *in situ* by reaction of phenyl bromide and NaN_3 in a 1:1 ratio.

[a] Determined by ^{1}H NMR spectroscopy.

Table S8 Monitoring of the cycloaddition of benzyl azide and phenylacetylene catalyzed by **2b** with time.

Time (h)	TON	TOF (h^{-1})	Conversion (%)[a]
0.08	18	218	6
0.17	124	746	41
0.25	200	800	66
0.50	264	527	87
0.75	288	384	95
1.0	297	297	98

Conditions: 0.33 mol% **2b**, 40 ºC, 40 eq. DIPEA (0.132 mmol), $[\text{phenylacetylene}]_{\text{solvent}}$ = 1.24 M (0.495 mmol in 0.4 mL of CH_2Cl_2), ratio azide/alkyne = 1:1.5, benzyl azide was formed *in situ* by reaction of phenyl bromide and NaN_3 in a 1:1 ratio.

[a] Determined by ^{1}H NMR spectroscopy.

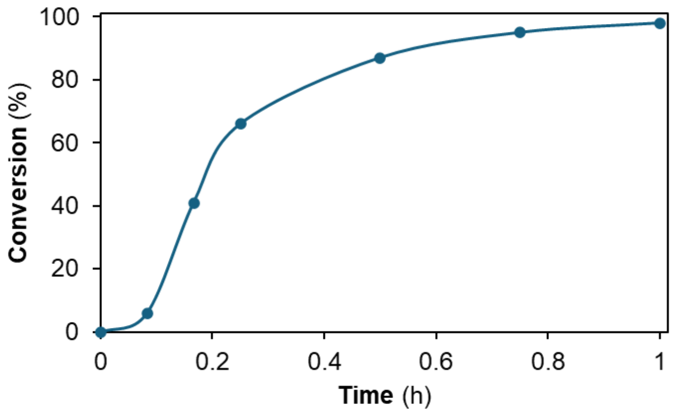

Figure S39 Graphical representation of the monitoring of the cycloaddition of benzyl azide and phenylacetylene catalyzed by **2b** with time.

Supplementary catalytic information

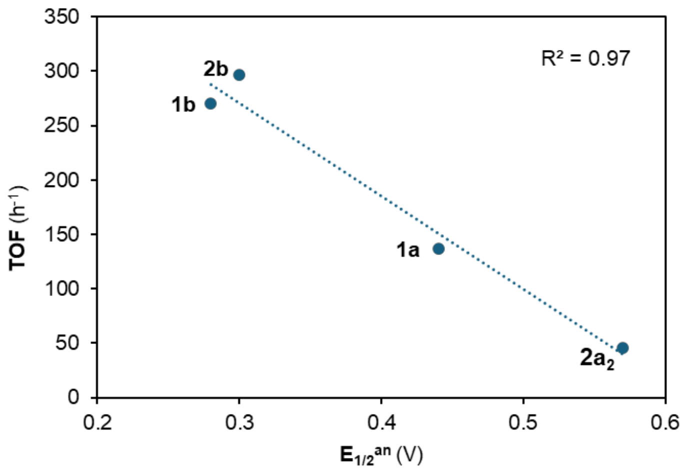

Figure S40 Turnover frequency (TOF) as a function of half-wave oxidation potentials ($E_{1/2}^{an}$) for complexes **1a**, **1b**, **2a$_2$** and **2b**.

Table S9 Selection of CuAAC catalyst systems and respective catalytic results for the cycloaddition of benzylazide and phenylacetylene.

Reference	Copper catalyst system	Temperature (ºC)	TOF (h^{-1})
Angew. Chem. Int. Ed., 2002, **41**, 2596	$CuSO_4$/sodium ascorbate	25	27
J. Am. Chem. Soc., 2002, **124**, 14397	CuI/DIPEA	25	5
Chem. Commun., 2008, 4747	*N*-heterocyclic carbene complexes	25	950
Dalton Trans., 2019, **48**, 8931	Mesoionic carbene complexes	20-22	99
Appl. Organomet. Chem., 2021, **35**, e6401	Isocyanide complexes	25	98
Organometallics, 2022, **41**, 3706	Silylene complexes	25	28
Organometallics, 2011, **30**, 6225	Phosphinite/phosphonite complexes	25	313
Dalton Trans., 2012, **41**, 5144	Diimine complexes	25	1920
**This work**	***Borane-tethered heteroscorpionate complexes***	**40**	**800**

NMR data of the products of the catalytic reactions

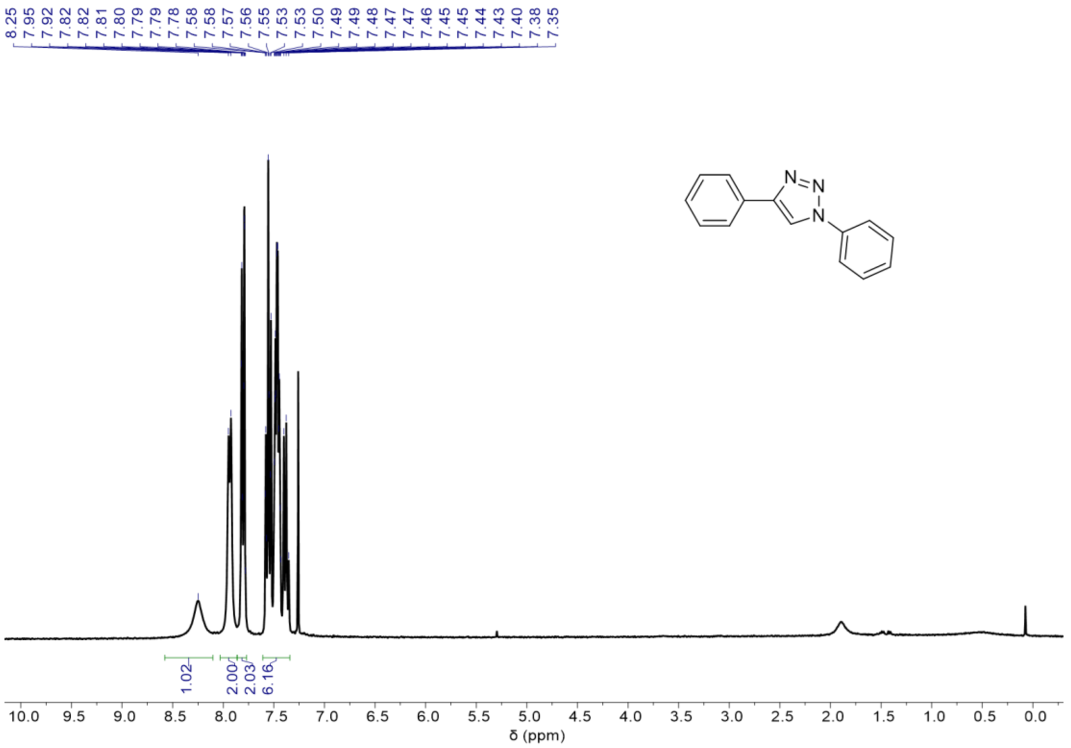

Figure S41 ^{1}H NMR spectrum (300 MHz, $CDCl_3$) of the cycloaddition product of phenylacetylene and azidobenzene, 1,4-diphenyl-1*H*-1,2,3-triazole.

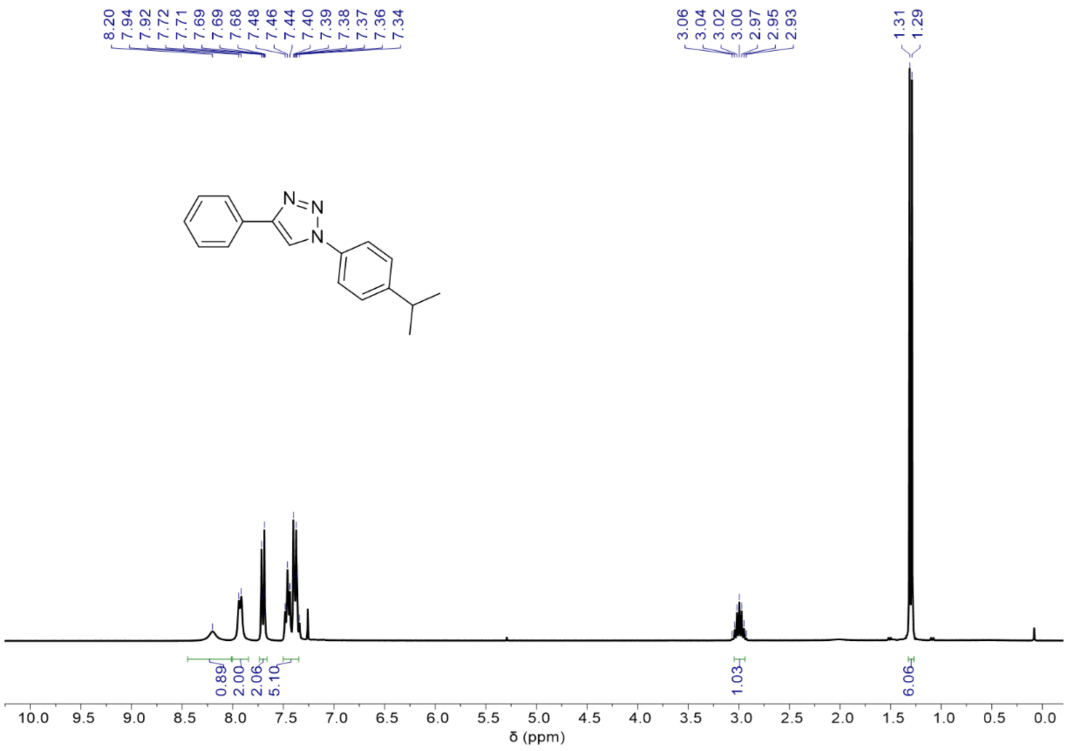

Figure S42 ^{1}H NMR spectrum (300 MHz, $CDCl_3$) of the cycloaddition product of phenylacetylene and 1-azido-4-isopropylbenzene, 1-(4-isopropylphenyl)-4-phenyl-1*H*-1,2,3-triazole.

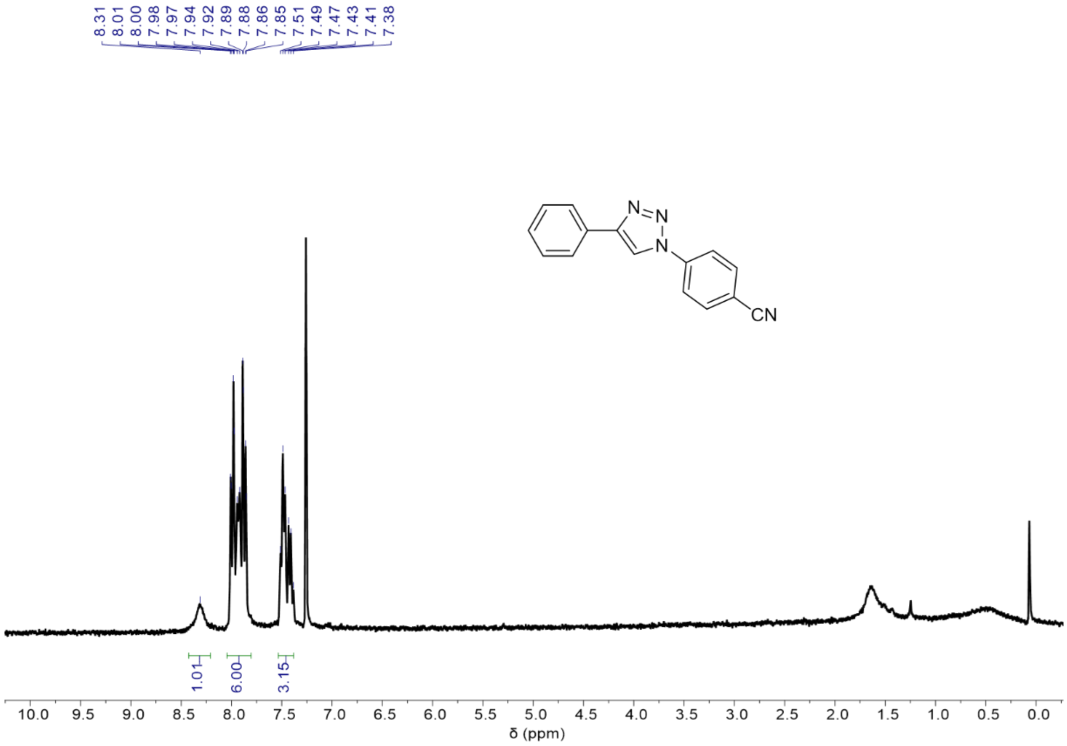

Figure S43 ^{1}H NMR spectrum (300 MHz, $CDCl_3$) of the cycloaddition product of phenylacetylene and 4-azidobenzonitrile, 4-(4-phenyl-1*H*-1,2,3-triazol-1-yl)benzonitrile.

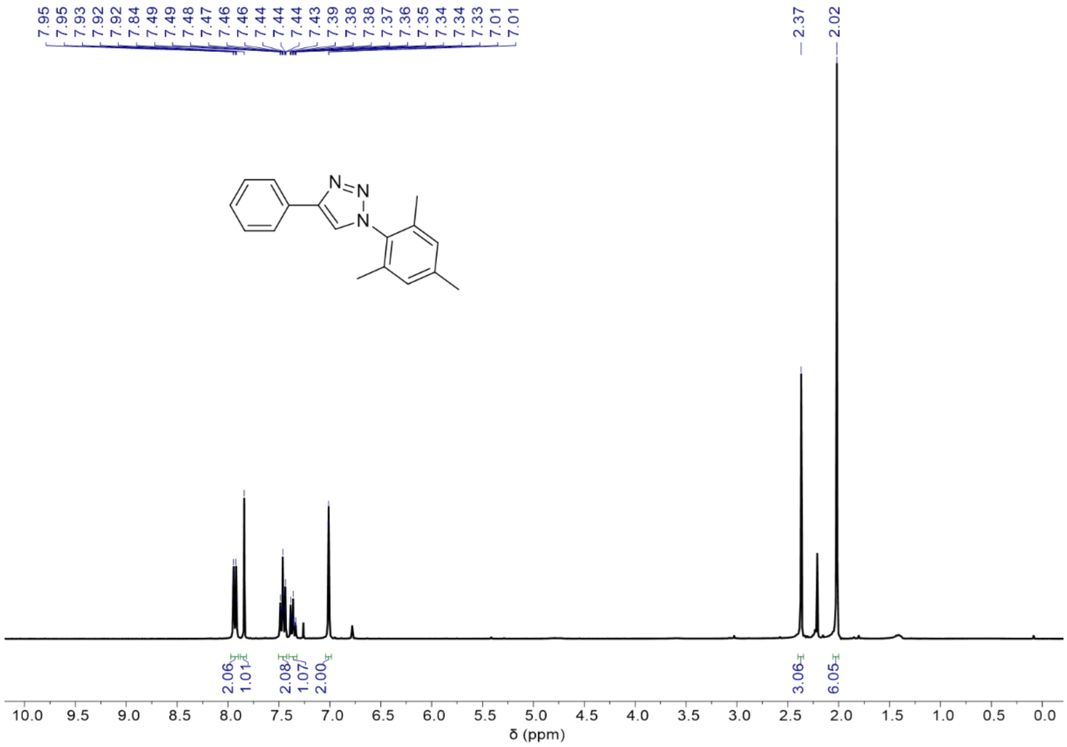

Figure S44 ^{1}H NMR spectrum (300 MHz, $CDCl_3$) of the cycloaddition product of phenylacetylene and 2-azido-1,3,5-trimethylbenzene, 1-mesityl-4-phenyl-1*H*-1,2,3-triazole.

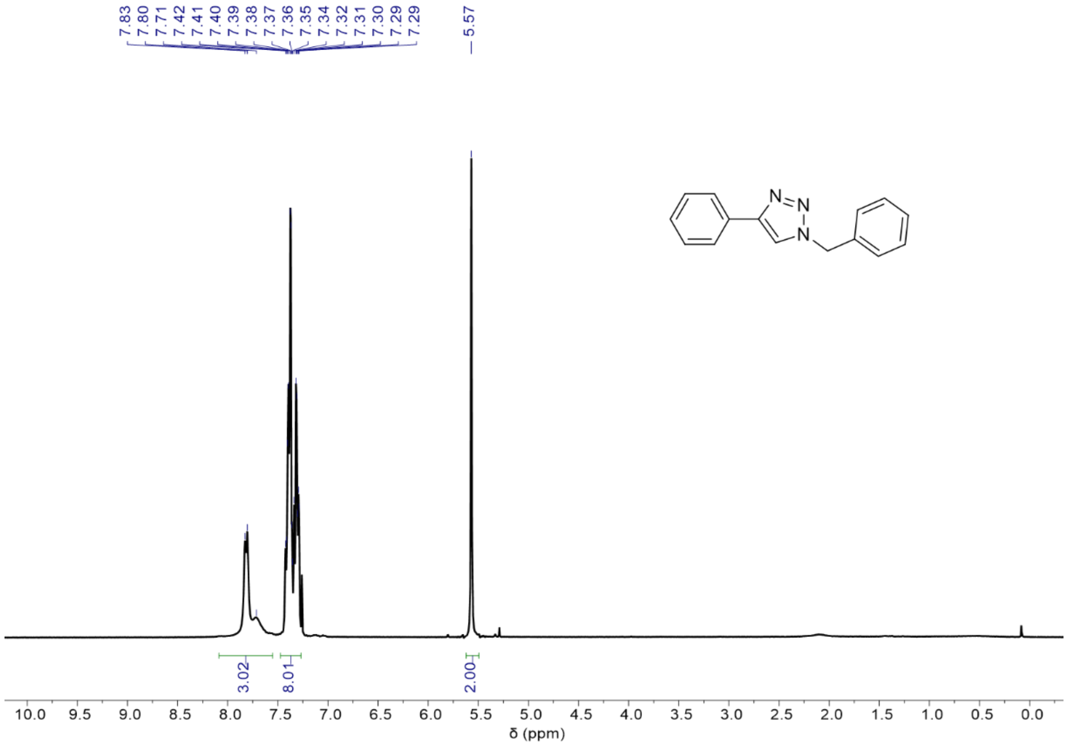

Figure S45 ^{1}H NMR spectrum (300 MHz, $CDCl_3$) of the cycloaddition product of phenylacetylene and (azidomethyl)benzene, 1-benzyl-4-phenyl-1*H*-1,2,3-triazole.

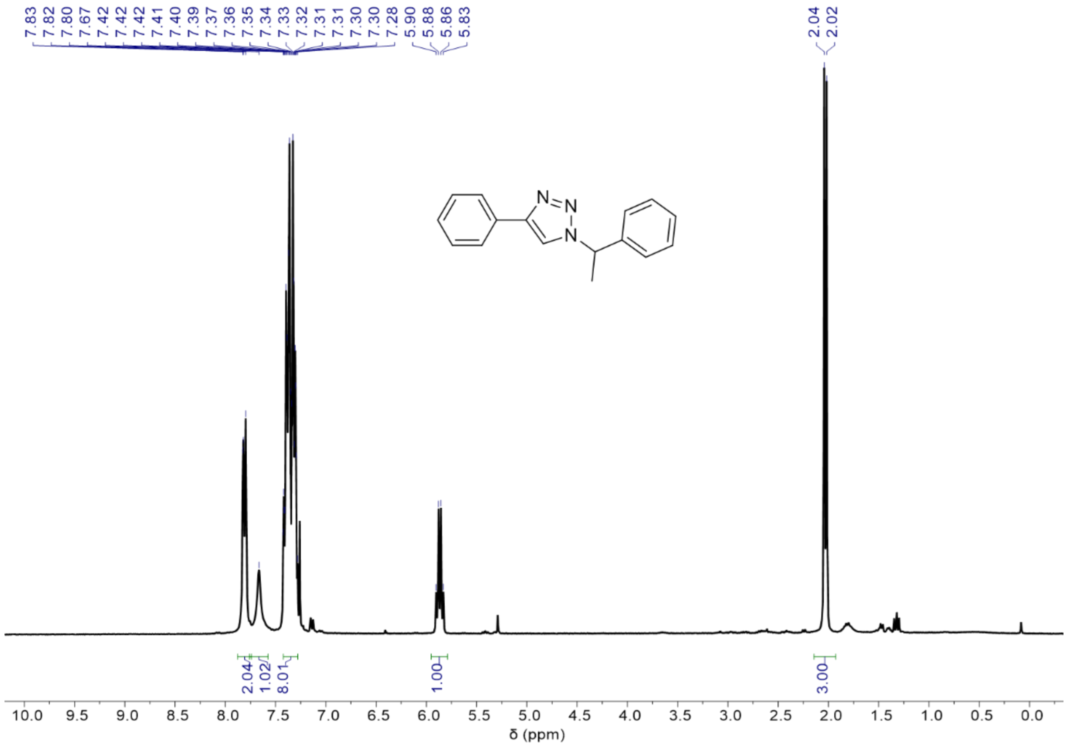

Figure S46 ^{1}H NMR spectrum (300 MHz, $CDCl_3$) of the cycloaddition product of phenylacetylene and (1-azidoethyl)benzene, 4-phenyl-1-(1-phenylethyl)-1*H*-1,2,3-triazole.

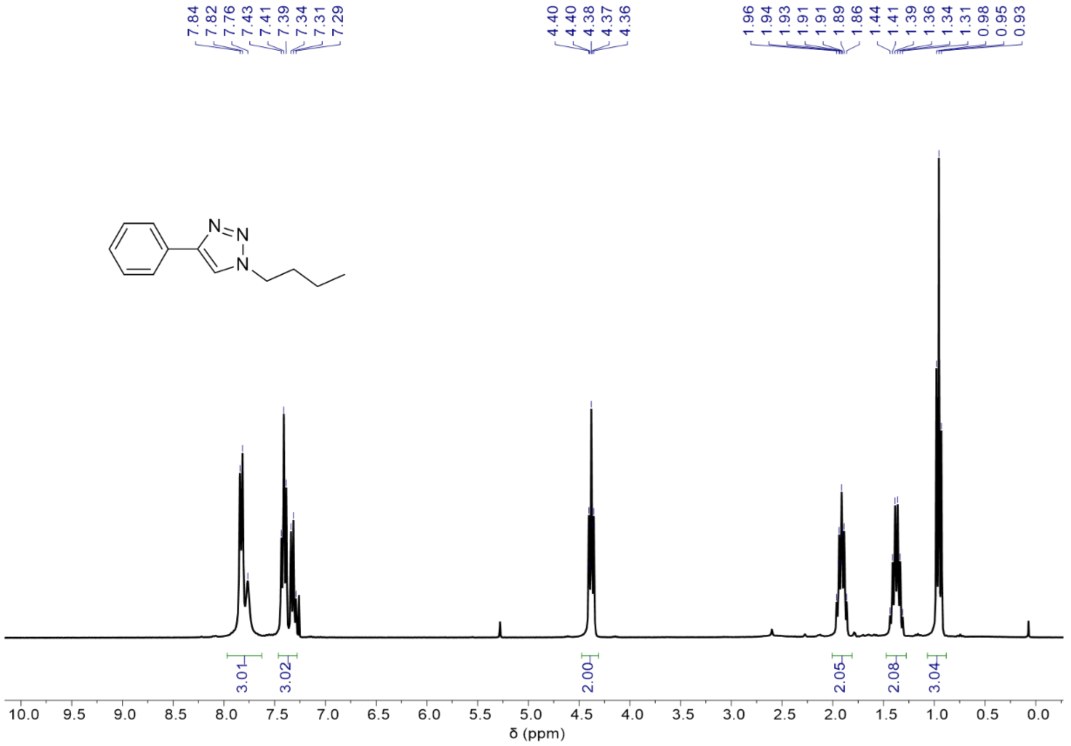

Figure S47 ^{1}H NMR spectrum (300 MHz, $CDCl_3$) of the cycloaddition product of phenylacetylene and 1-azidobutane, 1-butyl-4-phenyl-1*H*-1,2,3-triazole.

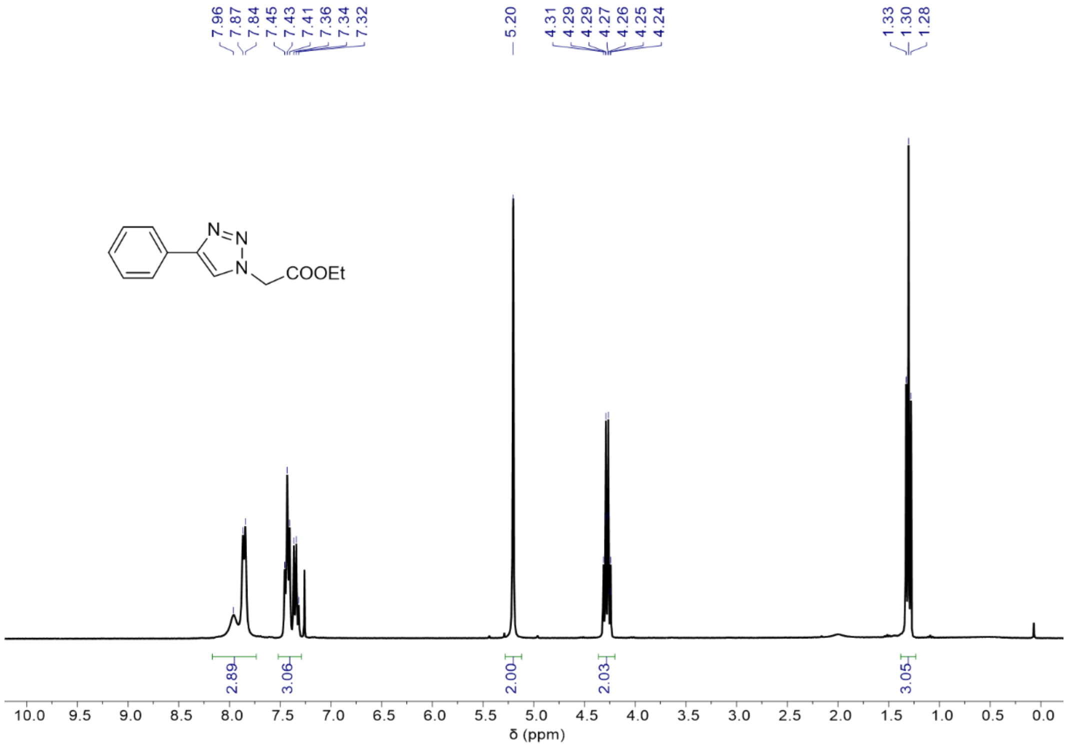

Figure S48 ^{1}H NMR spectrum (300 MHz, $CDCl_3$) of the cycloaddition product of phenylacetylene and ethyl 2-azidoacetate, ethyl 2-(4-phenyl-1*H*-1,2,3-triazol-1-yl)acetate.

Stoichiometric experiments between complexes 1a, 1b, 2a$_2$ and 2b and selected substrates

General procedure for the stoichiometric experiments between complexes 1a,b, 2a$_2$ and 2b with selected substrates: The desired complex (typically 25 mg) and one equivalent of mesitylazide or mesitylazide, phenylacetylene and DIPEA were dissolved in dichloromethane and stirred for 1 h at room temperature. All volatile materials were evaporated under vacuum. The resulting residues were redissolved in *ca.* 0.4 mL of $CDCl_3$, filtered and the solutions analyzed by ^1H and ^{11}B NMR spectroscopy.

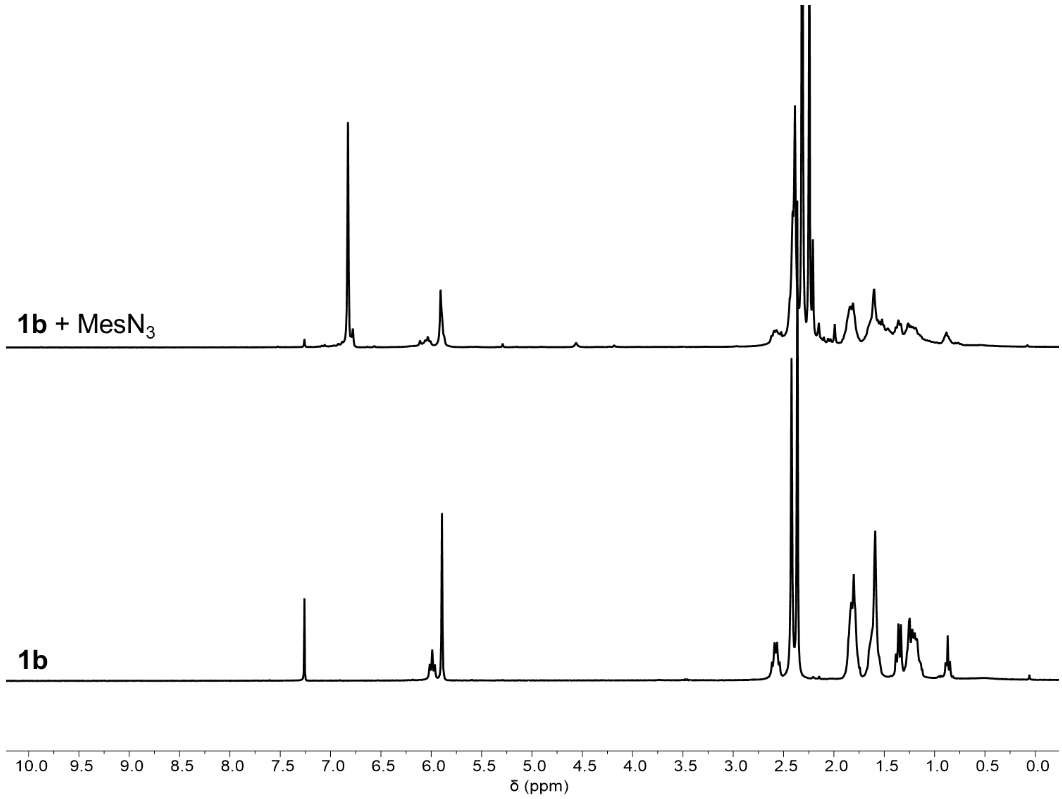

Figure S49 Stacking of the ^1H NMR spectra (300 MHz, $CDCl_3$) of the reaction of complex **1b** with $MesN_3$ after 1 h (top spectrum) and of complex **1b** (bottom spectrum).

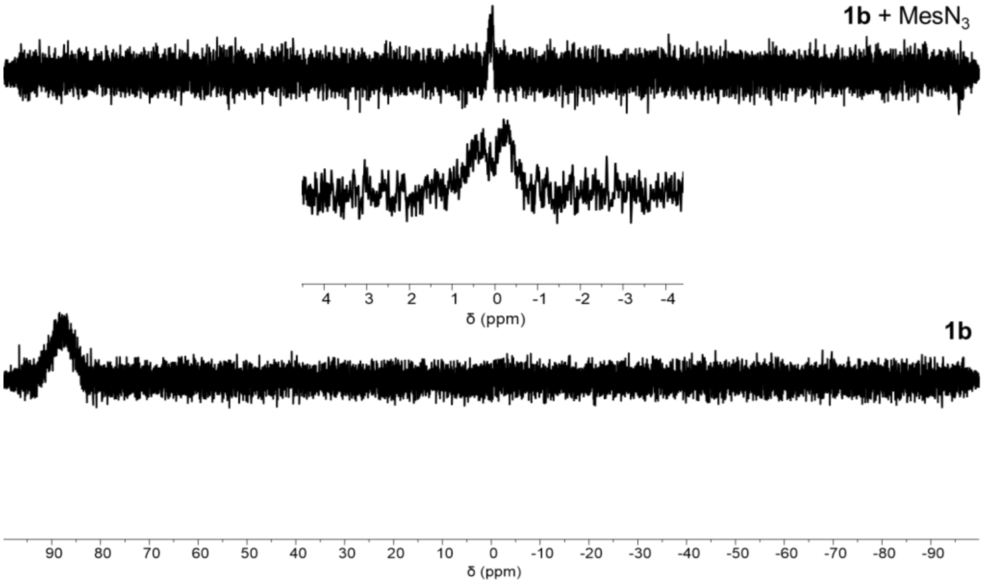

Figure S50 Stacking of the ^{11}B NMR spectra (96 MHz, $CDCl_3$) of the reaction of complex **1b** with $MesN_3$ after 1 h (top spectrum) and of complex **1b** (bottom spectrum). Inset shows a magnification of the zone of the spectrum close to 0 ppm.

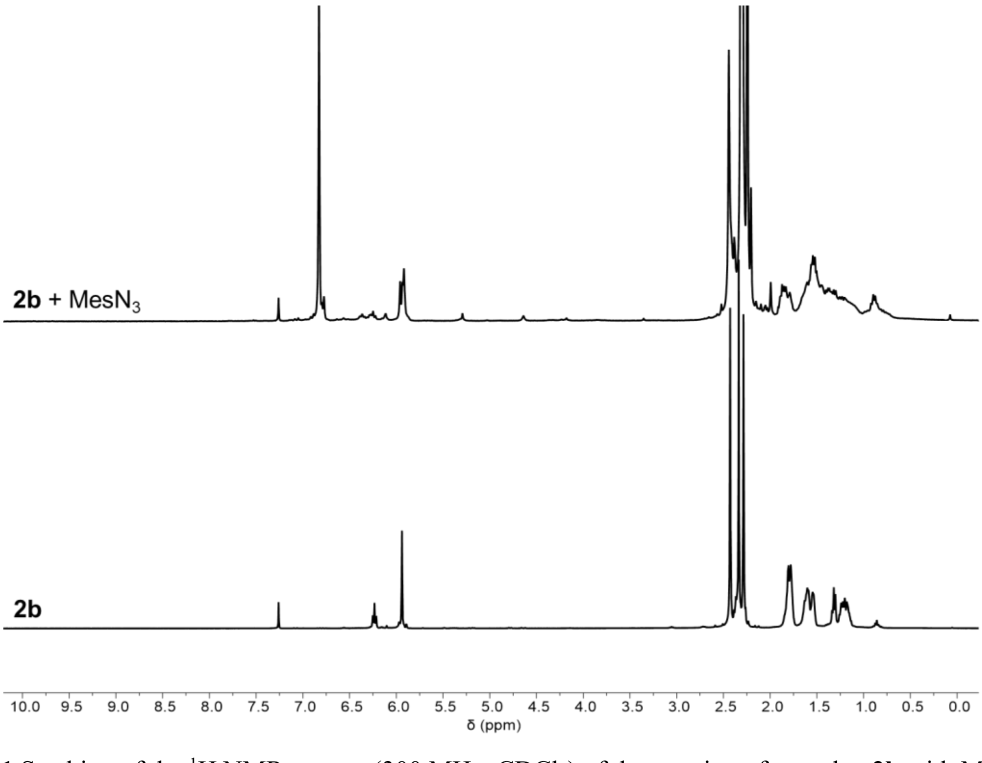

Figure S51 Stacking of the ^{1}H NMR spectra (300 MHz, $CDCl_3$) of the reaction of complex **2b** with $MesN_3$ after 1 h (top spectrum) and of complex **2b** (bottom spectrum).

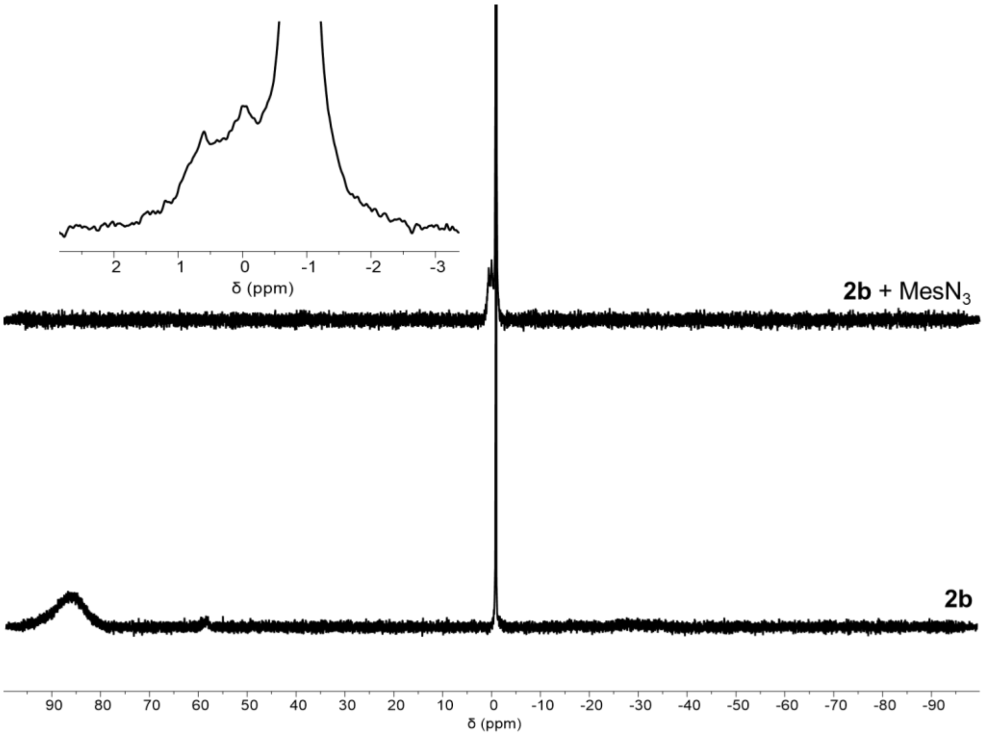

Figure S52 Stacking of the ^{11}B NMR spectra (96 MHz, $CDCl_3$) of the reaction of complex **2b** with $MesN_3$ after 1 h (top spectrum) and of complex **2b** (bottom spectrum). Inset shows a magnification of the zone of the spectrum close to 0 ppm.

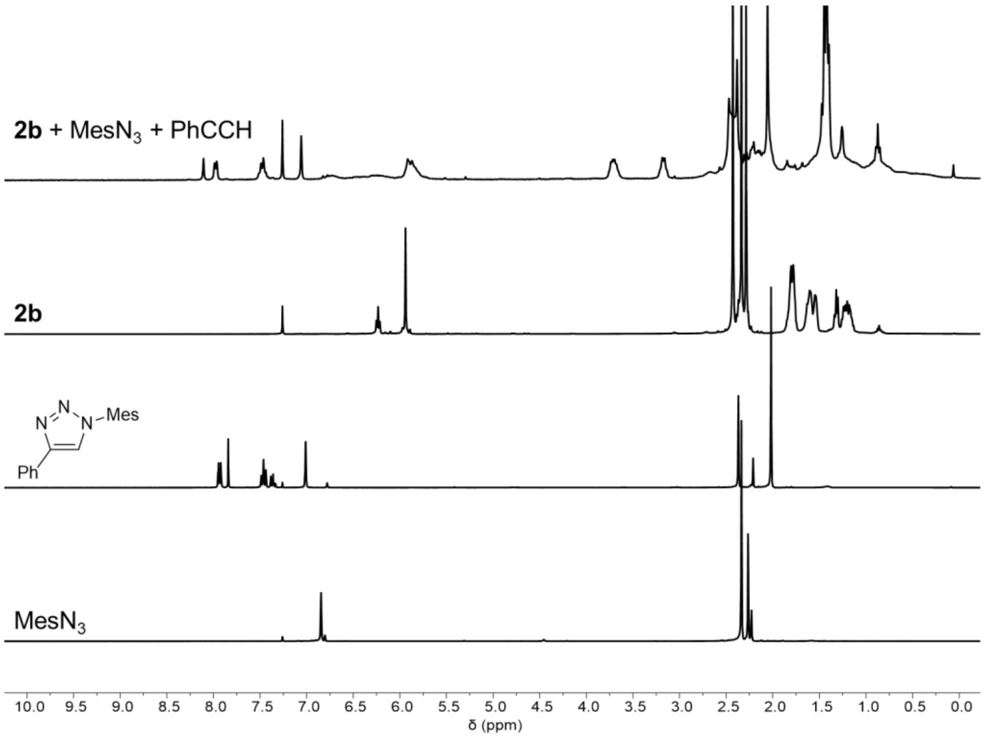

Figure S53 Stacking of the ^{1}H NMR spectra (300 MHz, $CDCl_3$) of the reaction of complex **2b** with $MesN_3$, phenylacetylene and DIPEA after 1 h, of complex **2b**, of 1-mesityl-4-phenyl-1*H*-1,2,3-triazole and of $MesN_3$, from top to bottom.

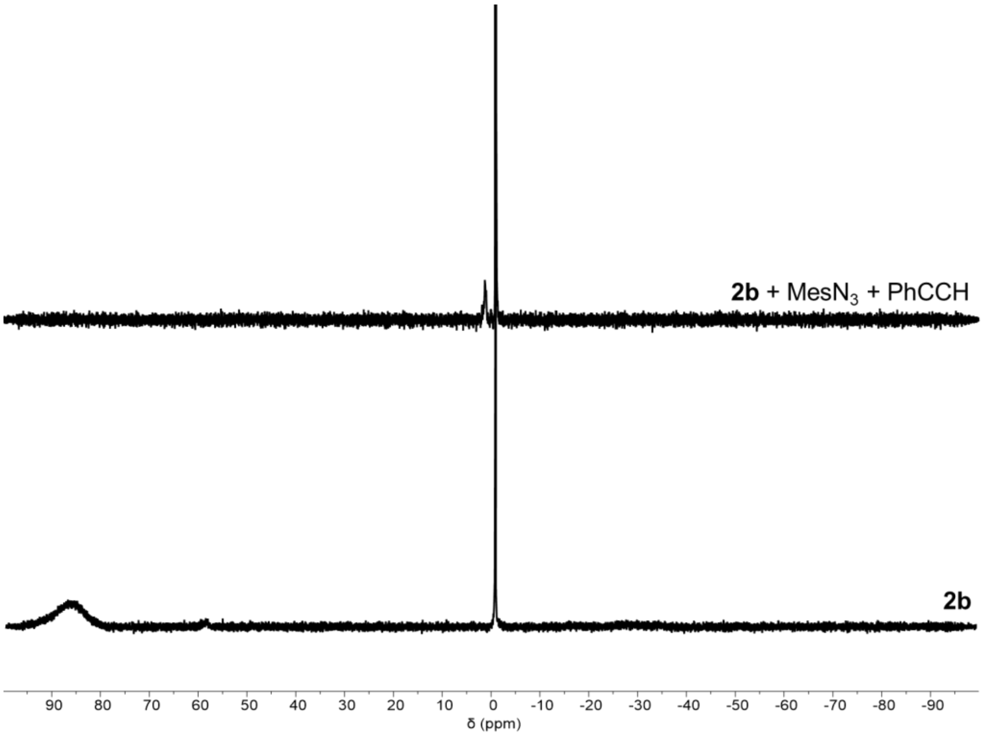

Figure S54 Stacking of the ^{11}B NMR spectra (96 MHz, $CDCl_3$) of the reaction of complex **2b** with $MesN_3$, phenylacetylene and DIPEA after 1 h (top spectrum) and of complex **2b** (bottom spectrum).

Atomic coordinates of the optimized geometries

1a

Cu	1.447610000	15.310755000	2.406404000
Cl	-0.319016000	15.283761000	1.123083000
N	3.160924000	14.072414000	4.578306000
N	3.210148000	16.319277000	2.649609000
N	2.043835000	13.863330000	3.848918000
C	2.566945000	18.716863000	5.469318000
H	1.897893000	18.751613000	4.612872000
H	2.932834000	19.665727000	5.850077000
N	4.053705000	16.105970000	3.680947000
C	2.925827000	11.899664000	4.480659000
H	3.072639000	10.836808000	4.607778000
C	4.964619000	12.826781000	5.809347000
H	5.825343000	13.278013000	5.302810000
H	5.208108000	11.780068000	5.999299000
H	4.847430000	13.318397000	6.781932000
C	1.888110000	12.544327000	3.773524000
C	3.881855000	17.039937000	1.754163000
C	2.913204000	17.560211000	6.029837000
H	3.583507000	17.562098000	6.891243000
C	2.444314000	16.221033000	5.550428000
H	1.618118000	16.327219000	4.840088000
H	2.079581000	15.610868000	6.385381000
C	3.248105000	17.433567000	0.465640000
H	3.791725000	17.005930000	-0.382737000
H	3.250877000	18.521480000	0.345657000
H	2.214596000	17.075446000	0.427971000
C	5.178459000	17.307333000	2.237260000
H	5.959171000	17.870870000	1.747443000
C	6.370405000	16.676976000	4.463987000
H	6.098429000	17.187328000	5.394578000
H	7.237293000	17.191190000	4.045629000
H	6.681522000	15.656260000	4.713086000
C	3.571209000	15.427829000	4.870836000
H	4.426707000	15.355285000	5.545884000
C	5.257162000	16.703994000	3.475609000
C	0.744509000	11.954924000	3.023171000
H	0.210972000	12.734560000	2.470760000
H	0.042679000	11.464435000	3.705900000
H	1.094766000	11.200682000	2.312142000
C	3.727363000	12.902515000	4.984028000

1b

Cu	4.287615000	6.872551000	4.712856000
N	2.817461000	5.447201000	4.532724000
C	2.906720000	9.586849000	4.066707000
N	1.510647000	5.715028000	4.736001000
N	1.525576000	8.108903000	4.783526000
C	1.130088000	6.902743000	5.481654000
H	0.038061000	6.908687000	5.510498000
C	0.715197000	4.717189000	4.265814000
C	-0.770143000	4.725211000	4.375838000
H	-1.107808000	4.741033000	5.418357000
H	-1.170492000	3.820881000	3.914487000
H	-1.218475000	5.583020000	3.862203000
C	1.606226000	10.041986000	3.761662000
H	1.331495000	10.959319000	3.261270000
C	1.676424000	6.864406000	6.908193000
H	1.345085000	5.919578000	7.355903000
H	2.771189000	6.827666000	6.861083000
C	0.741870000	9.075344000	4.232209000
C	2.870501000	4.272942000	3.908085000
C	1.224922000	8.047577000	7.755892000
H	0.126634000	8.108998000	7.746631000
H	1.593889000	8.972390000	7.296405000
C	-0.745846000	9.015714000	4.201100000
H	-1.111916000	8.144961000	3.645606000
H	-1.131982000	9.907440000	3.704342000
H	-1.180399000	8.983845000	5.206639000
B	1.101885000	6.842946000	10.145729000
N	2.837052000	8.415263000	4.693658000
C	1.720420000	7.934740000	9.197644000
H	1.536151000	8.885545000	9.728653000
H	2.818051000	7.851079000	9.215931000
C	4.178228000	3.683975000	3.507925000
H	4.328445000	2.708303000	3.980876000
H	4.996716000	4.348564000	3.801240000
H	4.224112000	3.537064000	2.424315000
C	4.224391000	10.221408000	3.784654000
H	4.342911000	11.145139000	4.360706000
H	4.311581000	10.479903000	2.725033000
H	5.038115000	9.537360000	4.044796000
C	1.564882000	3.774388000	3.723860000
H	1.276122000	2.845582000	3.253678000
C	0.580370000	6.988241000	12.603374000
H	0.490155000	8.083676000	12.566176000
H	0.910198000	6.753859000	13.625253000
C	1.672766000	6.572132000	11.588976000
H	2.567168000	7.172169000	11.806101000
C	-0.173968000	5.956526000	9.870578000
H	-0.602423000	6.108018000	8.869133000
C	-0.805971000	6.376903000	12.362314000
H	-1.544557000	6.922878000	12.962690000
H	-0.830710000	5.351320000	12.739616000
C	-1.250420000	6.397659000	10.894248000
H	-2.150495000	5.777570000	10.776289000
H	-1.559552000	7.421345000	10.636595000
C	2.086371000	5.083243000	11.657782000
H	2.444641000	4.841369000	12.668135000
H	2.953508000	4.944726000	10.996039000
C	0.996842000	4.083278000	11.251461000
H	1.456464000	3.097911000	11.104753000
H	0.289707000	3.950563000	12.074636000
C	0.239955000	4.469116000	9.973864000
H	-0.645582000	3.826633000	9.865635000
H	0.881163000	4.237773000	9.110219000
Cl	6.465644000	7.036634000	4.638045000

2a

Cu	4.307512000	6.767789000	4.689457000

N	2.782782000	5.323165000	4.595143000
C	2.997081000	9.563281000	4.118535000
N	1.497405000	5.696732000	4.785198000
N	1.608039000	8.096268000	4.840549000
C	1.186747000	6.895360000	5.536771000
H	0.098376000	6.947975000	5.588901000
C	0.626486000	4.763562000	4.311220000
C	-0.854098000	4.891788000	4.403252000
H	-1.202762000	4.935319000	5.440982000
H	-1.320530000	4.022263000	3.937769000
H	-1.223495000	5.781556000	3.881993000
C	1.707133000	10.112255000	3.991644000
H	1.441518000	11.085150000	3.604698000
C	1.743327000	6.835077000	6.966393000
H	1.341754000	5.931484000	7.437433000
H	2.831921000	6.720703000	6.906521000
C	0.836621000	9.154007000	4.468133000
C	2.738386000	4.144699000	3.977110000
C	1.377653000	8.055136000	7.755758000
H	1.855683000	8.986097000	7.452007000
N	5.543430000	6.682032000	6.317032000
C	-0.644720000	9.186621000	4.613017000
H	-1.133359000	8.391389000	4.039581000
H	-1.023080000	10.139390000	4.239642000
H	-0.951346000	9.093652000	5.660685000
N	2.923053000	8.343298000	4.647358000
C	0.521654000	8.057909000	8.776092000
H	0.290059000	8.967862000	9.321038000
H	0.032594000	7.147042000	9.114865000
C	3.985433000	3.430490000	3.587731000
H	4.004986000	2.421631000	4.010410000
H	4.863005000	3.974979000	3.943850000
H	4.057387000	3.331645000	2.499933000
C	4.307535000	10.162199000	3.743434000
H	4.441287000	11.138558000	4.218351000
H	4.374290000	10.312412000	2.661158000
H	5.126867000	9.508528000	4.051771000
C	1.400575000	3.752492000	3.781951000
H	1.042036000	2.846081000	3.316458000
N	5.524106000	6.728115000	3.030241000
C	6.178255000	6.705658000	2.076301000
C	6.239166000	6.631096000	7.240251000
C	6.998402000	6.678210000	0.880256000
H	7.797891000	7.419543000	0.959025000
H	7.443642000	5.687580000	0.755988000
H	6.386298000	6.905746000	0.003610000
C	7.110289000	6.567313000	8.398128000
H	6.556298000	6.851990000	9.296494000
H	7.491483000	5.550351000	8.522271000
H	7.953224000	7.251113000	8.268566000

2a$_2$

Cu	6.422712000	0.319001000	2.502593000
N	8.036948000	-0.899453000	2.946431000
N	8.905426000	1.365066000	1.182783000
N	9.209856000	-0.741241000	2.286806000
C	8.542559000	-0.199182000	-1.421262000
H	9.175945000	-0.788185000	-2.084042000
C	9.249134000	-0.032533000	1.024550000
H	10.289017000	-0.054177000	0.694641000
N	7.643036000	1.721874000	1.524067000
C	8.152135000	1.035844000	-1.845334000
H	7.641823000	1.737812000	-1.189928000
H	8.483889000	1.417966000	-2.807216000
C	8.360735000	-0.732911000	-0.022311000
H	7.322406000	-0.646386000	0.308136000
H	8.606067000	-1.798333000	0.007628000
C	7.625895000	3.056079000	1.530570000
N	5.792827000	1.234482000	4.345933000
C	10.223942000	-1.416180000	2.897583000
C	9.691820000	2.453174000	0.956410000
C	8.305162000	-1.669677000	4.000733000
C	6.396955000	3.814116000	1.893192000
H	6.109341000	4.502109000	1.092602000
H	6.563043000	4.415227000	2.792326000
H	5.567675000	3.130877000	2.090350000
C	9.667777000	-2.019653000	4.005123000
H	10.184124000	-2.633589000	4.728517000
C	8.890533000	3.555806000	1.171195000
H	9.187338000	4.590951000	1.085423000
C	7.248513000	-2.057073000	4.974858000
H	7.562977000	-1.832494000	5.997980000
H	7.047994000	-3.132011000	4.925265000
H	6.320065000	-1.518699000	4.773283000
C	5.559616000	1.700107000	5.379521000
C	11.122032000	2.372861000	0.552263000
H	11.734580000	1.872063000	1.309464000
H	11.519025000	3.381293000	0.427789000
H	11.252446000	1.847070000	-0.399662000
C	11.624143000	-1.442512000	2.392667000
H	11.698622000	-1.888530000	1.394781000
H	12.237181000	-2.044931000	3.064448000
H	12.067900000	-0.441878000	2.355560000
C	5.270053000	2.281934000	6.675945000
H	4.599251000	3.137453000	6.560769000
H	6.197535000	2.616871000	7.148057000
H	4.790967000	1.539008000	7.319269000
Cu	6.579315000	-0.318972000	-2.502595000
N	4.965072000	0.899476000	-2.946437000
N	4.096615000	-1.365054000	-1.182775000
N	3.792164000	0.741249000	-2.286815000
C	4.459468000	0.199214000	1.421251000
H	3.826076000	0.788221000	2.084022000
C	3.752896000	0.032545000	-1.024557000
H	2.713015000	0.054183000	-0.694640000
N	5.359005000	-1.721852000	-1.524070000
C	4.849893000	-1.035807000	1.845337000
H	5.360214000	-1.737779000	1.189940000
H	4.518130000	-1.417923000	2.807219000
C	4.641293000	0.732934000	0.022297000
H	5.679623000	0.646414000	-0.308150000
H	4.395954000	1.798354000	-0.007649000
C	5.376146000	-3.056057000	-1.530592000
N	7.209209000	-1.234432000	-4.345948000
C	2.778070000	1.416175000	-2.897595000
C	3.310244000	-2.453166000	-0.956344000
C	4.696847000	1.669682000	-4.000748000
C	6.605075000	-3.814087000	-1.893267000
H	6.892778000	-4.502012000	-1.092650000

H	6.438927000	-4.415271000	-2.792341000
H	7.434319000	-3.130839000	-2.090548000
C	3.334229000	2.019650000	-4.005136000
H	2.817875000	2.633577000	-4.728533000
C	4.111523000	-3.555793000	-1.171179000
H	3.814723000	-4.590939000	-1.085409000
C	5.753492000	2.057084000	-4.974874000
H	5.438994000	1.832581000	-5.998002000
H	5.954064000	3.132010000	-4.925221000
H	6.681919000	1.518655000	-4.773350000
C	7.442328000	-1.700144000	-5.379518000
C	1.880049000	-2.372861000	-0.552135000
H	1.267468000	-1.872063000	-1.309308000
H	1.483066000	-3.381296000	-0.427648000
H	1.749673000	-1.847075000	0.399798000
C	1.377868000	1.442494000	-2.392679000
H	1.303385000	1.888512000	-1.394793000
H	0.764825000	2.044908000	-3.064460000
H	0.934120000	0.441856000	-2.355572000
C	7.731692000	-2.282048000	-6.675952000
H	8.403023000	-3.137166000	-6.560888000
H	6.804213000	-2.617594000	-7.147638000
H	8.210101000	-1.538966000	-7.319599000

2b

Cu	4.265440000	6.825005000	4.685857000
N	2.798860000	5.332789000	4.529732000
C	2.860690000	9.557903000	4.075333000
N	1.498360000	5.633593000	4.738900000
N	1.520590000	8.033750000	4.771384000
C	1.133694000	6.828023000	5.479911000
H	0.042322000	6.839566000	5.510768000
C	0.675917000	4.643798000	4.297275000
C	-0.807391000	4.683226000	4.422278000
H	-1.133096000	4.727260000	5.467507000
H	-1.229780000	3.776979000	3.985629000
H	-1.244117000	5.537065000	3.892968000
C	1.550938000	10.015804000	3.839824000
H	1.251494000	10.953537000	3.395031000
C	1.671572000	6.797320000	6.909809000
H	1.316452000	5.864806000	7.365550000
H	2.765198000	6.738596000	6.875623000
C	0.712300000	9.020853000	4.298024000
C	2.813320000	4.144229000	3.929472000
C	1.233559000	7.996765000	7.742457000
H	0.137142000	8.073391000	7.727976000
H	1.617137000	8.913229000	7.277381000
N	5.503233000	6.726404000	6.308572000
C	-0.775172000	8.958816000	4.323035000
H	-1.160861000	8.106081000	3.753983000
H	-1.180751000	9.864965000	3.870473000
H	-1.169461000	8.895605000	5.343144000
B	1.065930000	6.811874000	10.139826000
N	2.827396000	8.356901000	4.649825000
C	1.716415000	7.886733000	9.188413000
H	1.531493000	8.841725000	9.711405000
H	2.813105000	7.793980000	9.217531000
C	4.093612000	3.498911000	3.527404000
H	4.196634000	2.513035000	3.990656000
H	4.942359000	4.116370000	3.830743000
H	4.138228000	3.357496000	2.442936000
C	4.153520000	10.227876000	3.763988000
H	4.188726000	11.229784000	4.201732000
H	4.288778000	10.337659000	2.683164000
H	4.989191000	9.645124000	4.158363000
C	1.498015000	3.671085000	3.766919000
H	1.184429000	2.738384000	3.321333000
C	0.605486000	7.019573000	12.595903000
H	0.604925000	8.118106000	12.549106000
H	0.930979000	6.767707000	13.614476000
C	1.647473000	6.506229000	11.570032000
H	2.588401000	7.038871000	11.768394000
C	-0.267769000	6.013083000	9.886378000
H	-0.698499000	6.181430000	8.888205000
C	-0.829363000	6.521814000	12.378935000
H	-1.511439000	7.134850000	12.980376000
H	-0.935753000	5.507192000	12.770113000
C	-1.290226000	6.562386000	10.916597000
H	-2.240476000	6.020009000	10.816532000
H	-1.514043000	7.604960000	10.647110000
C	1.943066000	4.990162000	11.652881000
H	2.304748000	4.734292000	12.658178000
H	2.780419000	4.771320000	10.972888000
C	0.766990000	4.076881000	11.288299000
H	1.138104000	3.052921000	11.157769000
H	0.068942000	4.024183000	12.126957000
C	0.019998000	4.498369000	10.016339000
H	-0.919442000	3.933414000	9.940619000
H	0.620034000	4.192627000	9.145494000
N	5.496673000	6.909539000	3.032505000
C	6.156352000	6.960407000	2.083480000
C	6.198325000	6.663450000	7.231519000
C	6.983253000	7.023870000	0.893423000
H	8.035578000	7.115885000	1.174477000
H	6.851572000	6.115799000	0.299280000
H	6.699486000	7.888525000	0.287767000
C	7.068486000	6.584334000	8.389312000
H	6.469494000	6.520323000	9.301560000
H	7.703931000	5.697706000	8.318303000
H	7.701892000	7.473729000	8.441320000

$[(La)Cu(MeCN)]^+$

Cu	3.857216000	6.828179000	3.699263000
N	2.457125000	5.378636000	3.993655000
C	2.487500000	9.594168000	3.562986000
N	1.285952000	5.706601000	4.588352000
N	1.380835000	8.102672000	4.648461000
C	1.198330000	6.888687000	5.421880000
H	0.174172000	6.924127000	5.795665000
C	0.345458000	4.741448000	4.404661000
C	-1.031952000	4.813976000	4.964462000
H	-1.032871000	4.835461000	6.059737000
H	-1.594006000	3.932097000	4.653947000
H	-1.575307000	5.694091000	4.604148000
C	1.225903000	10.139678000	3.857459000
H	0.857340000	11.119924000	3.593230000
C	2.155141000	6.820368000	6.621460000
H	1.926377000	5.902349000	7.173028000

H	3.181629000	6.724758000	6.245671000
C	0.540926000	9.168122000	4.559497000
C	2.259847000	4.198639000	3.403707000
C	2.024070000	8.022476000	7.506799000
H	2.394780000	8.963750000	7.102435000
C	-0.820780000	9.194256000	5.159922000
H	-1.466282000	8.410275000	4.749457000
H	-1.292740000	10.153942000	4.944879000
H	-0.782784000	9.076334000	6.248308000
N	2.574128000	8.359877000	4.062436000
C	1.491539000	7.994130000	8.727031000
H	1.420282000	8.889966000	9.335950000
H	1.125199000	7.070887000	9.170775000
C	3.336414000	3.536301000	2.618176000
H	3.484368000	2.506105000	2.953883000
H	4.279679000	4.076866000	2.728750000
H	3.080718000	3.502300000	1.554450000
C	3.614486000	10.203052000	2.805527000
H	3.850585000	11.197911000	3.193074000
H	3.359590000	10.314293000	1.746834000
H	4.508564000	9.579002000	2.880466000
C	0.945468000	3.760386000	3.641708000
H	0.487653000	2.843663000	3.300283000
N	5.711921000	6.747540000	3.209625000
C	6.836861000	6.691321000	2.943680000
C	8.244854000	6.621060000	2.611090000
H	8.799548000	7.368517000	3.184781000
H	8.634352000	5.626965000	2.846699000
H	8.382303000	6.815537000	1.543928000

4

Cu	3.504078000	5.891648000	3.752779000
N	1.897517000	4.653377000	3.989947000
C	2.303403000	8.579744000	2.782771000
N	0.784819000	5.161442000	4.568262000
N	1.122346000	7.502323000	4.225478000
C	0.828232000	6.468556000	5.200392000
H	-0.185966000	6.669418000	5.550812000
C	-0.251050000	4.284050000	4.512662000
C	-1.596267000	4.558221000	5.088135000
H	-1.554989000	4.723686000	6.170289000
H	-2.245425000	3.699422000	4.911824000
H	-2.072445000	5.429636000	4.625866000
C	1.073952000	9.248204000	2.905092000
H	0.758488000	10.136954000	2.378728000
C	1.774122000	6.493983000	6.400003000
H	1.436997000	5.705727000	7.083756000
H	2.786595000	6.209479000	6.083281000
C	0.336955000	8.538185000	3.831625000
C	1.569064000	3.440547000	3.542446000
C	1.810235000	7.839891000	7.116507000
H	0.781991000	8.179157000	7.305521000
H	2.263607000	8.587063000	6.452685000
C	-1.034427000	8.783512000	4.355782000
H	-1.715713000	7.956490000	4.128392000
H	-1.442838000	9.680818000	3.888776000
H	-1.038392000	8.943861000	5.439057000
B	1.896666000	6.970382000	9.641403000
N	2.322830000	7.523444000	3.599203000
C	2.565220000	7.750274000	8.443054000
H	2.736612000	8.765093000	8.840395000
H	3.585079000	7.367312000	8.281982000
C	2.550053000	2.574384000	2.832272000
H	2.949234000	1.803864000	3.500549000
H	3.384516000	3.164492000	2.446468000
H	2.074962000	2.063928000	1.991027000
C	3.457303000	8.923170000	1.907613000
H	4.099551000	9.674431000	2.378850000
H	3.105463000	9.330710000	0.957073000
H	4.070788000	8.043792000	1.701135000
C	0.226742000	3.167725000	3.855484000
H	-0.328560000	2.269535000	3.628774000
C	1.933160000	7.574497000	12.068143000
H	2.142321000	8.630655000	11.844819000
H	2.381016000	7.387172000	13.053327000
C	2.650493000	6.713595000	10.996296000
H	3.699773000	7.038070000	10.966828000
C	0.406480000	6.464135000	9.712244000
H	-0.155265000	6.605363000	8.776234000
C	0.413235000	7.384066000	12.150787000
H	-0.014969000	8.202936000	12.741326000
H	0.180720000	6.475331000	12.711165000
C	-0.285876000	7.345278000	10.786271000
H	-1.326682000	7.017829000	10.915717000
H	-0.344185000	8.371071000	10.393452000
C	2.640703000	5.192542000	11.279527000
H	3.105842000	4.993042000	12.254238000
H	3.292273000	4.707588000	10.537702000
C	1.260637000	4.527641000	11.228417000
H	1.391167000	3.438884000	11.203751000
H	0.716704000	4.729108000	12.154415000
C	0.407539000	4.949252000	10.025756000
H	-0.623754000	4.596864000	10.167392000
H	0.781142000	4.419836000	9.135056000
C	5.282975000	4.992844000	3.848418000
C	5.590943000	6.192100000	3.771505000
H	5.414063000	3.937616000	3.992844000
C	6.193686000	7.487065000	3.653030000
C	5.944756000	8.485423000	4.606996000
C	7.053967000	7.748084000	2.574780000
C	6.553681000	9.726911000	4.481706000
C	7.653258000	8.995564000	2.457693000
C	7.404249000	9.985044000	3.407100000
H	5.282111000	8.277682000	5.441671000
H	7.247793000	6.970523000	1.842713000
H	6.367980000	10.495492000	5.225547000
H	8.320553000	9.194600000	1.625019000
H	7.877763000	10.957487000	3.312708000

I^+

Cu	4.116328000	6.256287000	4.540391000
N	2.558229000	5.053265000	3.994167000
C	3.104249000	9.122574000	3.842110000
N	1.314190000	5.439562000	4.358892000
N	1.637478000	7.780670000	4.668919000
C	1.143639000	6.556196000	5.273662000
H	0.067181000	6.694217000	5.390991000
C	0.361042000	4.670829000	3.770125000

C	-1.099121000	4.849940000	3.997265000
H	-1.368077000	4.747004000	5.053939000
H	-1.646869000	4.086830000	3.442588000
H	-1.452345000	5.826082000	3.647595000
C	1.865309000	9.785466000	3.818718000
H	1.663784000	10.782073000	3.454469000
C	1.759833000	6.277087000	6.645479000
H	1.284064000	5.362679000	7.017567000
H	2.826604000	6.043080000	6.522907000
C	0.945010000	8.905577000	4.352094000
C	2.404290000	4.033865000	3.148424000
C	1.590773000	7.425160000	7.633209000
H	0.518601000	7.593161000	7.806146000
H	1.974811000	8.342561000	7.166256000
C	-0.515737000	9.070502000	4.585759000
H	-1.108536000	8.340438000	4.024123000
H	-0.823305000	10.063838000	4.256027000
H	-0.773426000	8.978947000	5.646245000
B	1.720747000	6.058062000	9.992467000
N	2.952238000	7.907263000	4.371817000
C	2.294361000	7.192948000	8.975669000
H	2.242326000	8.137399000	9.531896000
H	3.372618000	7.047769000	8.793060000
C	3.571771000	3.377066000	2.499043000
H	3.540376000	2.293279000	2.640699000
H	4.508785000	3.755897000	2.913339000
H	3.573376000	3.568101000	1.421317000
C	4.425809000	9.614990000	3.366600000
H	4.861293000	10.324163000	4.077919000
H	4.319635000	10.126492000	2.406527000
H	5.129929000	8.789722000	3.243899000
C	1.035704000	3.754334000	2.988220000
H	0.591615000	2.983089000	2.376206000
C	1.615088000	7.434525000	12.136550000
H	2.083151000	8.304209000	11.656102000
H	1.887060000	7.509216000	13.198725000
C	2.229915000	6.154585000	11.534128000
H	3.323384000	6.275924000	11.587953000
C	0.113964000	5.835121000	10.060369000
H	-0.326020000	5.711455000	9.056675000
C	0.089573000	7.557720000	12.006218000
H	-0.203141000	8.595543000	12.208099000
H	-0.394788000	6.968317000	12.789049000
C	-0.468966000	7.140946000	10.638187000
H	-1.562806000	7.059139000	10.711673000
H	-0.279357000	7.952561000	9.920735000
C	1.872327000	4.877277000	12.313377000
H	2.168384000	4.981074000	13.366587000
H	2.481788000	4.052064000	11.916648000
C	0.393997000	4.465794000	12.254720000
H	0.296771000	3.432295000	12.609595000
H	-0.177503000	5.065032000	12.968835000
C	-0.249213000	4.574892000	10.863805000
H	-1.340350000	4.495926000	10.971964000
H	0.052577000	3.699253000	10.270657000
C	7.330339000	2.211210000	6.840916000
C	7.118192000	3.146641000	7.854620000
C	6.211043000	1.686053000	6.189204000
H	7.975745000	3.567838000	8.373991000
H	6.349131000	0.945519000	5.404648000
C	5.844891000	3.569006000	8.236231000
C	4.914583000	2.063296000	6.531075000
C	4.746203000	3.014699000	7.554141000
N	3.396149000	3.369272000	7.773553000
N	2.970321000	4.047485000	8.686309000
N	2.327770000	4.601992000	9.457972000
C	3.724814000	1.461559000	5.841758000
H	4.042617000	0.769012000	5.058844000
H	3.100115000	0.906751000	6.549410000
H	3.086684000	2.230251000	5.393813000
C	5.694869000	4.589526000	9.327420000
H	5.149690000	4.198176000	10.193105000
H	6.677028000	4.907697000	9.682480000
H	5.168028000	5.487363000	8.983450000
C	8.716266000	1.754383000	6.488410000
H	8.997384000	0.878689000	7.084960000
H	8.789521000	1.466573000	5.436180000
H	9.458101000	2.533053000	6.684226000
C	6.048270000	6.551381000	5.303743000
C	5.815703000	5.350220000	5.097562000
H	5.948811000	4.283221000	5.145515000
C	6.567398000	7.855489000	5.598123000
C	7.714674000	8.308231000	4.927799000
C	5.949721000	8.672827000	6.557170000
C	8.232380000	9.564192000	5.217466000
C	6.479667000	9.924429000	6.841737000
C	7.617144000	10.373094000	6.171279000
H	8.190430000	7.670431000	4.189535000
H	5.061972000	8.319841000	7.073571000
H	9.120891000	9.911525000	4.699522000
H	6.005765000	10.551949000	7.590222000
H	8.027089000	11.353035000	6.396003000

II

29	4.368898000	6.800009000	3.954970000
7	2.852648000	5.328740000	3.590118000
6	2.937104000	9.459354000	3.393443000
7	1.549314000	5.570439000	3.834105000
7	1.577827000	7.944279000	4.074373000
6	1.198790000	6.688215000	4.691657000
1	0.109169000	6.700878000	4.764383000
6	0.749741000	4.592147000	3.331816000
6	-0.732636000	4.584621000	3.475949000
1	-1.046576000	4.525997000	4.524357000
1	-1.141728000	3.713409000	2.961486000
1	-1.194092000	5.475048000	3.034369000
6	1.633558000	9.982618000	3.279066000
1	1.346174000	10.959772000	2.919034000
6	1.807422000	6.510952000	6.082543000
1	1.424840000	5.555163000	6.457687000
1	2.892543000	6.398419000	5.962534000
6	0.783874000	8.991322000	3.726204000
6	2.902407000	4.191222000	2.904278000
6	1.501657000	7.640524000	7.056434000
1	0.413241000	7.706941000	7.202149000
1	1.810668000	8.588745000	6.596589000
6	-0.699403000	8.979627000	3.857450000
1	-1.159748000	8.178791000	3.267959000
1	-1.101549000	9.927255000	3.495195000

1	-1.017804000	8.860797000	4.899088000
5	1.766704000	6.243720000	9.381956000
7	2.884333000	8.223711000	3.885901000
6	2.199353000	7.475858000	8.413372000
1	2.012936000	8.394257000	8.986009000
1	3.288041000	7.476308000	8.250842000
6	4.208724000	3.637093000	2.451410000
1	4.349841000	2.617286000	2.822349000
1	5.025478000	4.259827000	2.827602000
1	4.264094000	3.605713000	1.358313000
6	4.242779000	10.083112000	3.042907000
1	4.357313000	11.053034000	3.536423000
1	4.320316000	10.249035000	1.963465000
1	5.062636000	9.427444000	3.350017000
6	1.596014000	3.688722000	2.720098000
1	1.304136000	2.786564000	2.201933000
6	1.431924000	7.519685000	11.571484000
1	1.793253000	8.456396000	11.126775000
1	1.666319000	7.594627000	12.643381000
6	2.214471000	6.351919000	10.940691000
1	3.282390000	6.604359000	11.031647000
6	0.194690000	5.815607000	9.399153000
1	-0.200921000	5.678332000	8.379784000
6	-0.093206000	7.451854000	11.402207000
1	-0.523194000	8.436082000	11.629159000
1	-0.517391000	6.776395000	12.150769000
6	-0.558160000	7.018655000	10.004678000
1	-1.636452000	6.802420000	10.041380000
1	-0.447912000	7.871254000	9.320331000
6	2.002254000	5.010723000	11.665287000
1	2.257683000	5.107945000	12.730670000
1	2.719992000	4.286709000	11.254555000
6	0.589842000	4.416286000	11.552815000
1	0.618215000	3.366019000	11.871207000
1	-0.069815000	4.912181000	12.270962000
6	-0.032758000	4.494087000	10.150082000
1	-1.109003000	4.279120000	10.228876000
1	0.384699000	3.681197000	9.538166000
6	7.124572000	2.204800000	5.779064000
6	7.021173000	3.412393000	6.470326000
6	5.960053000	1.468363000	5.560509000
1	7.911237000	4.019870000	6.613261000
1	6.015823000	0.528519000	5.015479000
6	5.808329000	3.896240000	6.953564000
6	4.718401000	1.903941000	6.015180000
6	4.663327000	3.114125000	6.724987000
7	3.355686000	3.483750000	7.124197000
7	3.057315000	4.262388000	8.001479000
7	2.542406000	4.888272000	8.812311000
6	3.471857000	1.108583000	5.754215000
1	3.706454000	0.198029000	5.197332000
1	2.975960000	0.819394000	6.686764000
1	2.745119000	1.689018000	5.174834000
6	5.753295000	5.227866000	7.642711000
1	5.465470000	5.146955000	8.697307000
1	6.731905000	5.708348000	7.597856000
1	5.053479000	5.905471000	7.141680000
6	8.459157000	1.723571000	5.286542000
1	9.053620000	1.306567000	6.108242000
1	8.348385000	0.941997000	4.529932000
1	9.036834000	2.545906000	4.853157000
6	7.424216000	6.365999000	4.358016000
6	6.221906000	6.566010000	4.174736000
6	8.801696000	6.101045000	4.593635000
6	9.562120000	5.335597000	3.687533000
6	9.446350000	6.578871000	5.751704000
6	10.902005000	5.059972000	3.933430000
6	10.785459000	6.296020000	5.993829000
6	11.522247000	5.534984000	5.088357000
1	9.079835000	4.967126000	2.787142000
1	8.876788000	7.183386000	6.451737000
1	11.468506000	4.471127000	3.216367000
1	11.259564000	6.677059000	6.894733000
1	12.569464000	5.317623000	5.278090000

III

29	4.289680000	6.713869000	4.786574000
7	2.743023000	5.357706000	4.615231000
6	2.897107000	9.417851000	3.979452000
7	1.450902000	5.644077000	4.877870000
7	1.512958000	8.029804000	4.849537000
6	1.119123000	6.858426000	5.602973000
1	0.029170000	6.887517000	5.669758000
6	0.620740000	4.650972000	4.462875000
6	-0.856867000	4.681587000	4.646625000
1	-1.140573000	4.731962000	5.703959000
1	-1.290837000	3.769973000	4.232510000
1	-1.320300000	5.530376000	4.131316000
6	1.597010000	9.897173000	3.714059000
1	1.324954000	10.794105000	3.176679000
6	1.711741000	6.849603000	7.010666000
1	1.353141000	5.937410000	7.503619000
1	2.802660000	6.760596000	6.938975000
6	0.730124000	8.986359000	4.281193000
6	2.749030000	4.172596000	4.007167000
6	1.338139000	8.081258000	7.826690000
1	0.246808000	8.217796000	7.811014000
1	1.769979000	8.967089000	7.345749000
6	-0.758294000	8.973767000	4.329691000
1	-1.179494000	8.087526000	3.841896000
1	-1.143405000	9.850151000	3.805643000
1	-1.138576000	9.009091000	5.356715000
5	1.095555000	6.964520000	10.245087000
7	2.826761000	8.286893000	4.678482000
6	1.820899000	7.964256000	9.272058000
1	1.734952000	8.943708000	9.775059000
1	2.903818000	7.765056000	9.293434000
6	4.023973000	3.556354000	3.546858000
1	4.153486000	2.558019000	3.976066000
1	4.869862000	4.183568000	3.842616000
1	4.038586000	3.454059000	2.457144000
6	4.210781000	9.985934000	3.568546000
1	4.260209000	11.056325000	3.788839000
1	4.372180000	9.855071000	2.493614000
1	5.024733000	9.471404000	4.085220000
6	1.430529000	3.688453000	3.895046000
1	1.107890000	2.756011000	3.455167000
6	0.584127000	7.212603000	12.702967000
1	0.603035000	8.310843000	12.650667000

1	0.885282000	6.960173000	13.729424000
6	1.632612000	6.677872000	11.698393000
1	2.578891000	7.197393000	11.905542000
6	-0.259229000	6.197861000	9.986955000
1	-0.667471000	6.362129000	8.978922000
6	-0.855727000	6.740013000	12.462506000
1	-1.538657000	7.368391000	13.047921000
1	-0.986646000	5.728727000	12.855864000
6	-1.286855000	6.779154000	10.991143000
1	-2.249122000	6.259670000	10.878654000
1	-1.479826000	7.824865000	10.709431000
6	1.907091000	5.158172000	11.793742000
1	2.256893000	4.904272000	12.804244000
1	2.746382000	4.925597000	11.122467000
6	0.720854000	4.259358000	11.426001000
1	1.078574000	3.228824000	11.308669000
1	0.012080000	4.225459000	12.257346000
6	-0.004272000	4.679323000	10.140273000
1	-0.954389000	4.131847000	10.060671000
1	0.600095000	4.351143000	9.281555000
6	5.648188000	7.093819000	1.723155000
1	4.818718000	6.768878000	2.349228000
6	5.448655000	7.295450000	0.361412000
1	4.461741000	7.134868000	-0.066369000
6	6.506032000	7.692456000	-0.454282000
1	6.351904000	7.848348000	-1.518448000
6	7.765076000	7.881834000	0.113214000
1	8.600747000	8.188028000	-0.511131000
6	7.964748000	7.685132000	1.474048000
1	8.943051000	7.832113000	1.920728000
6	6.907578000	7.290920000	2.308843000
6	7.135300000	7.100401000	3.742375000
6	6.210179000	6.860263000	4.765971000
7	7.040904000	6.793662000	5.844519000
7	8.353277000	6.974447000	5.529559000
7	8.400470000	7.162792000	4.256207000
6	5.900759000	8.932827000	7.386709000
1	6.781653000	9.286470000	6.842121000
1	5.073822000	8.905365000	6.667566000
1	5.657268000	9.658301000	8.167615000
6	6.148114000	7.574811000	7.976683000
6	5.853555000	7.305622000	9.314862000
1	5.446892000	8.104619000	9.932782000
6	5.833320000	5.810304000	11.346587000
1	6.729708000	6.008567000	11.946664000
1	5.038098000	6.458656000	11.726615000
1	5.542938000	4.771881000	11.533195000
6	6.098218000	6.056477000	9.886970000
6	6.641458000	5.054691000	9.081509000
1	6.839887000	4.073987000	9.509726000
6	6.952412000	5.277863000	7.740614000
6	6.695635000	6.545372000	7.201629000
6	7.556494000	4.200465000	6.888570000
1	7.619282000	3.256181000	7.435306000
1	8.559990000	4.486994000	6.559441000
1	6.963661000	4.042733000	5.981198000

IV⁺

29	4.094138000	7.113207000	3.739095000
7	2.936629000	5.450663000	3.490032000
6	2.084553000	9.473099000	3.722468000
7	1.816971000	5.348468000	4.241288000
7	1.405585000	7.662336000	4.670226000
6	1.532469000	6.344838000	5.263984000
1	0.545353000	6.100620000	5.660184000
6	1.105953000	4.236013000	3.928501000
6	-0.150291000	3.842648000	4.623058000
1	0.007346000	3.705085000	5.698282000
1	-0.504196000	2.893125000	4.219468000
1	-0.946988000	4.581262000	4.482708000
6	0.728687000	9.648128000	4.043965000
1	0.119422000	10.519681000	3.855446000
6	2.532955000	6.299111000	6.416607000
1	2.437517000	5.304026000	6.860073000
1	3.559634000	6.378642000	6.031022000
6	0.319788000	8.477017000	4.651272000
6	2.935544000	4.402290000	2.665596000
6	2.267829000	7.374731000	7.464463000
1	1.184962000	7.438790000	7.634912000
1	2.559056000	8.344804000	7.037109000
6	-1.007867000	8.099986000	5.208433000
1	-1.419882000	7.209696000	4.721234000
1	-1.711249000	8.917883000	5.045909000
1	-0.961829000	7.912031000	6.286534000
5	2.264554000	6.112518000	9.897335000
7	2.485677000	8.263360000	4.119338000
6	2.974309000	7.126969000	8.805962000
1	3.062694000	8.099826000	9.303526000
1	4.016383000	6.838391000	8.595214000
6	4.018235000	4.198906000	1.663477000
1	4.645991000	3.340724000	1.925356000
1	4.653440000	5.085122000	1.597066000
1	3.597915000	4.006438000	0.672643000
6	3.007703000	10.427878000	3.050149000
1	3.396488000	11.163798000	3.761688000
1	2.486600000	10.970256000	2.257580000
1	3.860774000	9.906717000	2.610505000
6	1.802905000	3.608331000	2.912661000
1	1.518741000	2.695368000	2.410342000
6	2.244037000	7.807983000	11.840287000
1	2.863898000	8.528677000	11.290580000
1	2.466887000	7.988558000	12.901876000
6	2.694292000	6.384093000	11.450128000
1	3.782109000	6.371960000	11.600557000
6	0.633468000	6.157057000	9.905811000
1	0.205608000	5.939909000	8.911843000
6	0.765814000	8.137031000	11.585467000
1	0.630851000	9.226131000	11.614173000
1	0.156851000	7.756196000	12.409617000
6	0.213429000	7.600229000	10.258159000
1	-0.884057000	7.680337000	10.276472000
1	0.544492000	8.270254000	9.451974000
6	2.107632000	5.309420000	12.384451000
1	2.380631000	5.524069000	13.427876000
1	2.584739000	4.345140000	12.157195000
6	0.586935000	5.130899000	12.299646000
1	0.304449000	4.196098000	12.802034000
1	0.095492000	5.919458000	12.875616000
6	0.033425000	5.114290000	10.867231000

1	-1.061399000	5.218772000	10.909043000
1	0.217034000	4.119602000	10.440608000
6	5.969112000	8.015700000	3.981430000
6	6.064613000	6.852337000	3.559089000
1	6.536597000	5.928680000	3.284916000
6	6.126519000	9.369090000	4.427255000
6	6.876966000	10.264979000	3.649364000
6	5.553299000	9.798983000	5.633848000
6	7.048024000	11.574384000	4.079589000
6	5.735271000	11.109396000	6.055154000
6	6.478313000	11.998274000	5.278927000
1	7.320913000	9.925776000	2.718815000
1	4.973953000	9.102843000	6.232937000
1	7.630644000	12.265455000	3.478514000
1	5.297599000	11.438522000	6.992481000
1	6.616815000	13.022453000	5.611690000
6	0.741028000	-0.155358000	5.942380000
6	1.886500000	0.541879000	5.556287000
6	0.186100000	0.119194000	7.194003000
1	2.332075000	0.339799000	4.585067000
1	-0.705099000	-0.417202000	7.510970000
6	2.488030000	1.490038000	6.383968000
6	0.743855000	1.055574000	8.063072000
6	1.893634000	1.728397000	7.629218000
7	2.497918000	2.696867000	8.496835000
7	1.951536000	3.890417000	8.665069000
7	2.736486000	4.561329000	9.467429000
6	0.124043000	1.324931000	9.402784000
1	-0.569703000	0.525851000	9.671642000
1	0.877118000	1.400855000	10.192934000
1	-0.431581000	2.268295000	9.401234000
6	3.732503000	2.208002000	5.941851000
1	4.627945000	1.791085000	6.416593000
1	3.858831000	2.114849000	4.861154000
1	3.702500000	3.273867000	6.186889000
6	0.134854000	-1.200695000	5.050388000
1	0.432391000	-1.063208000	4.007691000
1	0.459481000	-2.202386000	5.354283000
1	-0.957431000	-1.186460000	5.101073000
6	3.648142000	2.584782000	9.194092000
6	3.820463000	3.798026000	9.822596000
6	4.968082000	4.154855000	10.673493000
6	5.293227000	3.321929000	11.749766000
6	5.794533000	5.243192000	10.376631000
6	6.415932000	3.584938000	12.527694000
6	6.916107000	5.501762000	11.155327000
6	7.228400000	4.675725000	12.232535000
1	4.649494000	2.480169000	11.989711000
1	5.556949000	5.886058000	9.536356000
1	6.651700000	2.939390000	13.368123000
1	7.549611000	6.351474000	10.919571000
1	8.103617000	4.882353000	12.841032000
1	4.253056000	1.691784000	9.189299000

MeCN

C	5.680020000	-2.407502000	2.376609000
N	5.679914000	-2.407500000	1.217845000
C	5.679966000	-2.407497000	3.830529000
H	6.705708000	-2.407508000	4.208014000
H	5.167084000	-3.295832000	4.207983000
H	5.167076000	-1.519170000	4.207978000

PhCCH

C	-1.108916000	0.988999000	3.802700000
C	-1.108916000	0.988999000	5.012321000
H	-1.108916000	0.988999000	2.736444000
C	-1.108916000	0.988999000	6.439585000
C	-2.318999000	0.988999000	7.149798000
C	0.101168000	0.988999000	7.149798000
C	-2.314278000	0.988999000	8.538690000
C	0.096447000	0.988999000	8.538690000
C	-1.108916000	0.988999000	9.236689000
H	-3.254811000	0.988999000	6.600345000
H	1.036980000	0.988999000	6.600345000
H	-3.256277000	0.988999000	9.079198000
H	1.038446000	0.988999000	9.079198000
H	-1.108916000	0.988999000	10.322714000

$MesN_3$

C	7.190107000	1.248602000	6.968349000
C	7.566284000	2.252143000	7.858453000
C	5.831231000	1.098506000	6.687994000
H	8.617134000	2.368827000	8.115544000
H	5.509512000	0.310259000	6.010553000
C	6.640155000	3.108258000	8.455582000
C	4.865999000	1.929985000	7.248127000
C	5.287252000	2.949437000	8.116490000
N	4.244823000	3.731935000	8.683736000
N	4.393753000	4.937698000	8.860712000
N	4.376683000	6.054614000	9.077436000
C	3.409558000	1.755569000	6.931645000
H	3.255085000	0.894440000	6.277144000
H	2.819969000	1.609268000	7.842283000
H	3.001791000	2.642297000	6.434986000
C	7.095269000	4.128241000	9.461367000
H	6.476842000	4.101474000	10.365030000
H	8.127603000	3.931058000	9.759092000
H	7.057101000	5.152337000	9.073434000
C	8.215090000	0.360362000	6.321376000
H	9.135426000	0.316526000	6.910207000
H	7.840881000	-0.660617000	6.201254000
H	8.482219000	0.727561000	5.323209000

1-Mes-4-Ph-triazole

C	7.159080000	1.361801000	6.763723000
C	7.300566000	1.799455000	8.081043000
C	5.959801000	1.623440000	6.098975000
H	8.231839000	1.608798000	8.609768000
H	5.836293000	1.292665000	5.070084000
C	6.281673000	2.484822000	8.741797000
C	4.910941000	2.303463000	6.715547000
C	5.094028000	2.715281000	8.040154000
N	4.030562000	3.406942000	8.694480000
N	3.871867000	4.735638000	8.550342000
N	2.835471000	5.080215000	9.242422000
C	3.630130000	2.595838000	5.989271000

H	3.676018000	2.235203000	4.959364000
H	2.773936000	2.119328000	6.479126000
H	3.428631000	3.671383000	5.972969000
C	6.452689000	2.970478000	10.152335000
H	5.777759000	2.453041000	10.842916000
H	7.476097000	2.806859000	10.497069000
H	6.228295000	4.039015000	10.228048000
C	8.285868000	0.655761000	6.063344000
H	8.927971000	0.125299000	6.771808000
H	7.912651000	-0.068109000	5.333398000
H	8.916642000	1.370070000	5.521050000
C	3.070009000	2.895853000	9.497695000
C	2.297741000	3.984128000	9.851277000
C	1.110235000	4.071949000	10.702187000
C	0.577854000	2.940909000	11.332225000
C	0.485290000	5.309958000	10.895642000
C	-0.551783000	3.044347000	12.135332000
C	-0.643720000	5.409925000	11.699367000
C	-1.167888000	4.279447000	12.322435000
H	1.048867000	1.970875000	11.195899000
H	0.901588000	6.184230000	10.405565000
H	-0.952326000	2.156861000	12.617140000
H	-1.117840000	6.377241000	11.840128000
H	-2.050697000	4.359847000	12.950086000
H	3.024330000	1.843960000	9.732140000

References

1 F. Kloss, U. Köhn, B. O. Jahn, M. D. Hager, H. Görls and U. S. Schubert, *Chem. Asian J.*, 2011, **6**, 2816–2824.

2 G. J. Kubas, *Inorganic Syntheses: Reagents for Transition Metal Complex and Organometallic Syntheses*, vol. 28, Wiley, 1990, pp. 90–91.

3 T. F. C. Cruz, V. Loupy and L. F. Veiros, *Inorg. Chem.*, 2024, **63**, 8244–8256.

4 G. R. Fulmer, A. J. M. Miller, N. H. Sherden, H. E. Gottlieb, A. Nudelman, B. M. Stoltz, J. E. Bercaw and K. I. Goldberg, *Organometallics*, 2010, **29**, 2176–2179.

5 SMART Software for the CCD Detector System Version 5.625, Bruker AXS Inc., Madison, WI, USA, 2001.

6 SAINT Software for the CCD Detector System, Version 7.03, Bruker AXS Inc., Madison, WI, USA, 2004.

7 G. M. Sheldrick, SADABS, *Program for Empirical Absorption Correction*, University of Göttingen, Göttingen, 1996.

8 M. C. Burla, R. Caliandro, B. Carrozzini, G. L. Cascarano, C. Cuocci, C. Giacovazzo, M. Mallamo, A. Mazzone and G. Polidori, *J. Appl. Cryst.*, 2015, **48**, 306–309.

9 (a) G. M. Sheldrick, *Acta Cryst., Sect. C: Struct. Chem.*, 2015, **71**, 3–8. (b) C. B. Hübschle, G. M. Sheldrick and B. Dittrich, *J.Appl. Cryst.*, 2011, **44**, 1281–1284.

10 (a) L. J. Farrugia, *J. Appl. Cryst.* 1999, **32**, 837–838. (b) L. J. Farrugia, *J. Appl. Cryst.*, 2012, **45**, 849–854.

11 C. F. Macrae, I. Sovago, S. J. Cottrell, P. T. A. Galek, P. McCabe, E. Pidcock, M. Platings, G. P. Shields, J. S. Stevens, M. Towler and P. A. Wood, *J. Appl. Cryst.*, 2020, **53**, 226–235.

12 Gaussian 09, Revision A.01, M. J. Frisch, G. W. Trucks, H. B. Schlegel, G. E. Scuseria, M. A. Robb, J. R. Cheeseman, G. Scalmani, V. Barone, B. Mennucci, G. A. Petersson, H. Nakatsuji, M. Caricato, X. Li, H. P. Hratchian, A. F. Izmaylov, J. Bloino, G. Zheng, J. L. Sonnenberg, M. Hada, M. Ehara, K. Toyota, R. Fukuda, J. Hasegawa, M. Ishida, T. Nakajima, Y. Honda, O. Kitao, H. Nakai, T. Vreven, J. A. Montgomery Jr., J. E. Peralta, F. Ogliaro, M. Bearpark, J. J. Heyd, E. Brothers, K. N. Kudin, V. N. Staroverov, R. Kobayashi, J Normand, K. Raghavachari, A. Rendell, J. C. Burant, S. S. Iyengar, J. Tomasi, M. Cossi, N. Rega, J. M. Millam, M. Klene, J. E. Knox, J. B. Cross, V. Bakken, C. Adamo, J. Jaramillo, R. Gomperts, R. E. Stratmann, O. Yazyev, A. J. Austin, R. Cammi, C. Pomelli, J. W. Ochterski, R. L. Martin, K. Morokuma, V. G. Zakrzewski, G.

A. Voth, P. Salvador, J. J. Dannenberg, S. Dapprich, A. D. Daniels, Ö Farkas, J. B. Foresman, J. V. Ortiz, J. Cioslowski and D. J. Fox, Gaussian, Inc., Wallingford CT, 2009.

13 W. J. Hehre, L. Radom, P. v.R. Schleyer and J. A. Pople, *Ab Initio Molecular Orbital Theory*, John Wiley & Sons, New York, 1986.

14 (a) J. P. Perdew, K. Burke and M. Ernzerhof, "Generalized Gradient Approximation Made Simple," *Phys. Rev. Lett.*, 1996, **77**, 3865–3868. (b) J. P. Perdew, *Phys. Rev. B* 1986, **33**, 8822–8824.

15 (a) R. Ditchfield, W. J. Hehre and J. A. Pople, *J. Chem. Phys.*, 1971, **54**, 724–728. (b) W. J. Hehre, R. Ditchfield and J. A. Pople, *J. Chem. Phys.*, 1972, **56**, 2257–2261. (c) P. C. Hariharan and J. A. Pople, *Mol. Phys.* 1974, **27**, 209–214. (d) M. S. Gordon, *Chem. Phys. Lett.*, 1980, **76**, 163–168. (e) P. C. Hariharan and J. A. Pople, *Theor. Chim. Acta*, 1973, **28**, 213–222.

16 (a) U. Haeusermann, M. Dolg, H. Stoll, H. Preuss, P. Schwerdtfeger and R. M. Pitzer, *Mol. Phys.*, 1993, **78**, 1211–1224. (b) W. Kuechle, M. Dolg, H. Stoll and H. Preuss, *J. Chem. Phys.*, 1994, **100**, 7535–7542. (c) T. Leininger, A. Nicklass, H. Stoll, M. Dolg and P. Schwerdtfeger, *J. Chem. Phys.*, 1996, **105**, 1052–1059.

17 A. W. Ehlers, M. Böhme, S. Dapprich, A. Gobbi, A. Höllwarth, V. Jonas, K. F. Köhler, R. Stegmann, A. Veldkamp and G. Frenking, *Chem. Phys. Lett.*, 1993, **208**, 111–114.

18 L. S. Campbell-Verduyn, L. Mirfeizi, R. A. Dierckx, P. H. Elsinga and B. L. Feringa, *Chem. Commun.*, 2009, 2139–2141.
